# Synthesis of N,O-bidentate organic difluoroboron complexes and their photophysical studies

**DOI:** 10.1186/s13065-023-00974-7

**Published:** 2023-06-12

**Authors:** Jin Guang, Weibin Fan, Zhiqi Liu, Deguang Huang

**Affiliations:** 1 State Key Laboratory of Structural Chemistry, Fujian Institute of Research on the Structure of Matter, Chinese Academy of Sciences, Fuzhou, 350002 Fujian, China; 2 University of Chinese Academy of Sciences, Beijing, 100049 China

**Keywords:** Fluorescent dyes, Synthesis, Photophysical studies, Large Stokes shifts

## Abstract

We disclose a novel boron trifluoride induced C–H activation and difluoroboronation at room temperature, thus providing a straightforward gateway to a series of N,O-bidentate organic BF_2_ complexes. The scope of the method is demonstrated with 24 examples. All the synthesized compounds exhibit fluorescence and some of them have large Stokes shifts.

**Graphical Abstract:**

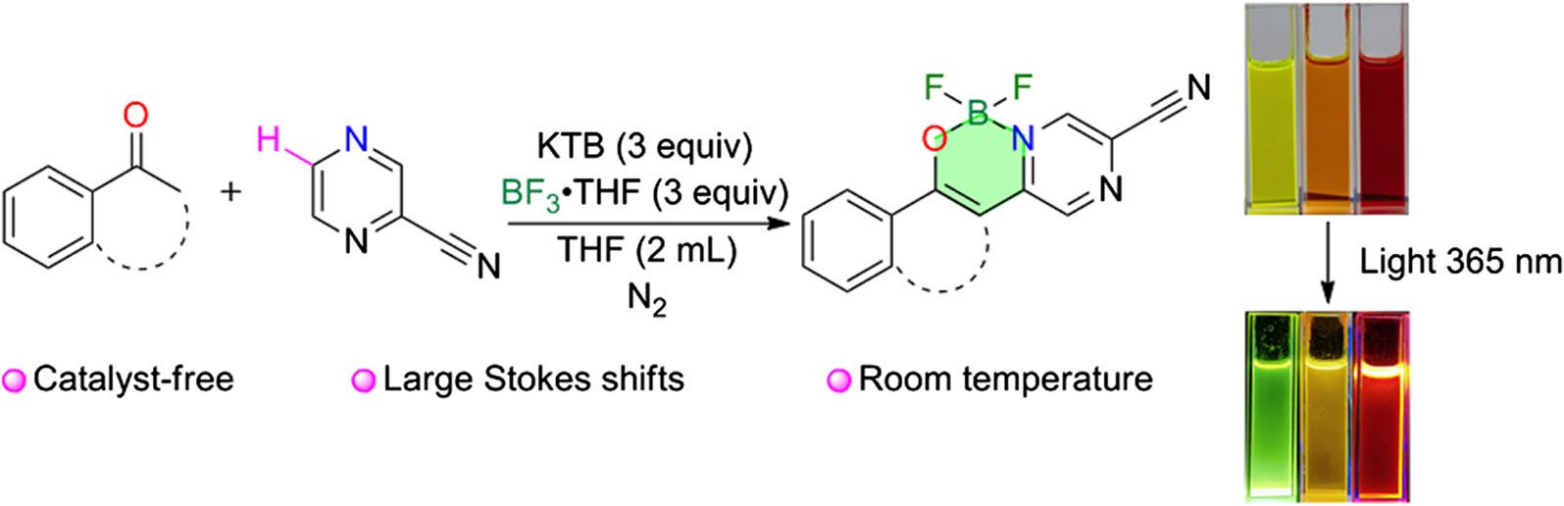

**Supplementary Information:**

The online version contains supplementary material available at 10.1186/s13065-023-00974-7.

## Introduction

Small organic fluorescent dyes are popular and have been attracting attention owing to their remarkable optical properties. Due to the characteristics associated with high fluorescence intensity and quantum yields, sharp absorption and fluorescence emission spectra, high photo- and chemical stability, organic difluoroboron (BF_2_) complexes have played increasingly important roles in many fields involving biological fluorophores, fluorescent indicators, photosensitizers, light-emitting materials, photodynamic therapy, laser dye and solar cells as well. Fluorescent materials with large Stokes shift play an important role in biological field [1–8]. Besides, fluorescence properties of organic difluoroboron complexes can be managed by changing the structure of the organic ligand. Since these advantages make organic difluoroboron complexes becomes a research hotspot.

**Fig. 1:**
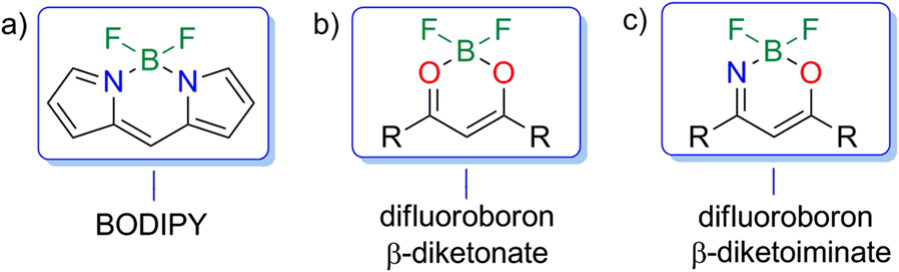
Three types of organic difluoroboron BF_2_ complexes

At present, these organic difluoroboron complexes are classified into three categories: N,N-bidentate (Fig. 1a), O,O-bidentate (Fig. 1b) and N,O-bidentate (Fig. 1c) complexes [9]. After years of research, the synthetic strategies.for access to N,N-bidentate and O,O-bidentate organic difluoroboron complexes are gradually mature. Boradipyrromethene (BODIPY) (Fig. 1a), a typical fluorescent dye of N,N-bidentate complexes, as well as its derivatives are continuously explored [10–21]. Meanwhile, extensive research has also been carried on difluoroboron β-diketonate (Fig. 1b) [22–24]. However, N,O-bidentate organic BF_2_ complexes are seldom investigated [25–28], especially for the synthesis of such compounds.

**Scheme 1:**
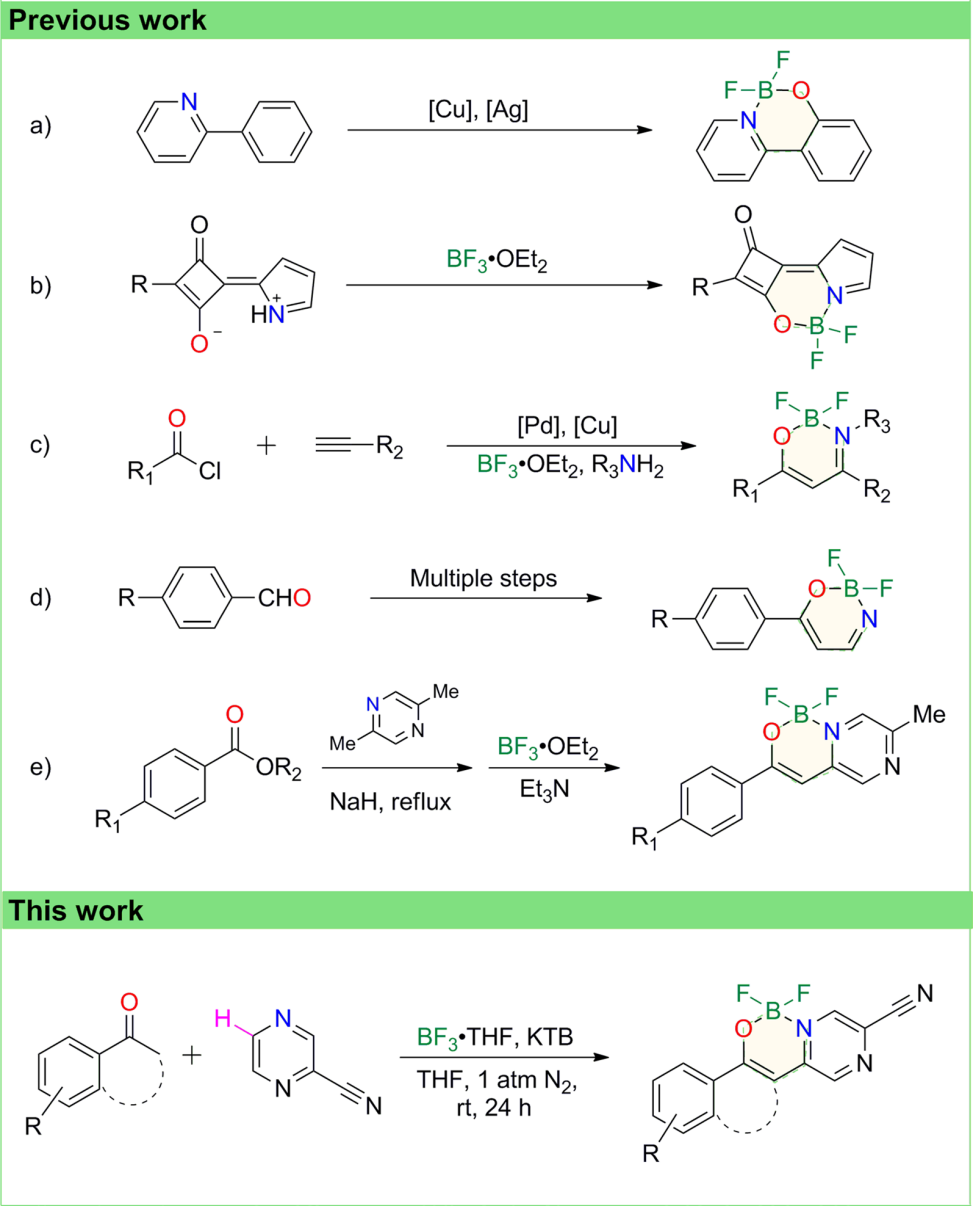
Different strategies to synthesize N,O-bidentate organic BF_2_ complexes

In recent year, a new class of N,O-bidentate organic BF_2_ complexes are prepared by 2-phenylpyridine derivatives (Scheme 1a) [28]. The reaction involves a Cu(OAc)_2_ catalyzed bimetallic system for the efficient C-H activation of 2-phenylpyridines, but restricted substrates limit the structural diversity of N,O-bidentate organic BF_2_ complexes. Besides, other published approaches to build the N,O-bidentate organic BF_2_ motifs suffer from their disadvantages. For example, substrates of the reaction in Scheme 1b are limited to a few of non-commercial available compounds endowed with specific structures [29]. Another adverse factor is that noble metal catalysts are required for the complete transformation of substrates (Scheme 1c) [30]. Moreover, characterized by tedious experimental operations and high energy consumption, multi-step methods usually have their limitations (Scheme 1d) [31]. In addition, harsh conditions and low yield are drawbacks for further industrial production and commercial application (Scheme 1e) [26]. From the perspective of foundation and application, more effective and convenient strategies are required for the development of the N,O-bidentate organic BF_2_ complexes synthetic chemistry.

In this paper, we report the synthesis and fluorescence properties of a novel pyrazineboron complex. Boron trifluoride and potassium t-butoxide induce regioselective C–H activation and difluoroboronation at room temperature. N,O-bidentate complexes are obtained by the reaction and excellent fluorescence properties of the products are shown in further results. In comparison to what described in the literatures, our synthesis method is superior in catalyst-free system, low-cost process and step economy.

## Results and discussions

**Scheme 2:**
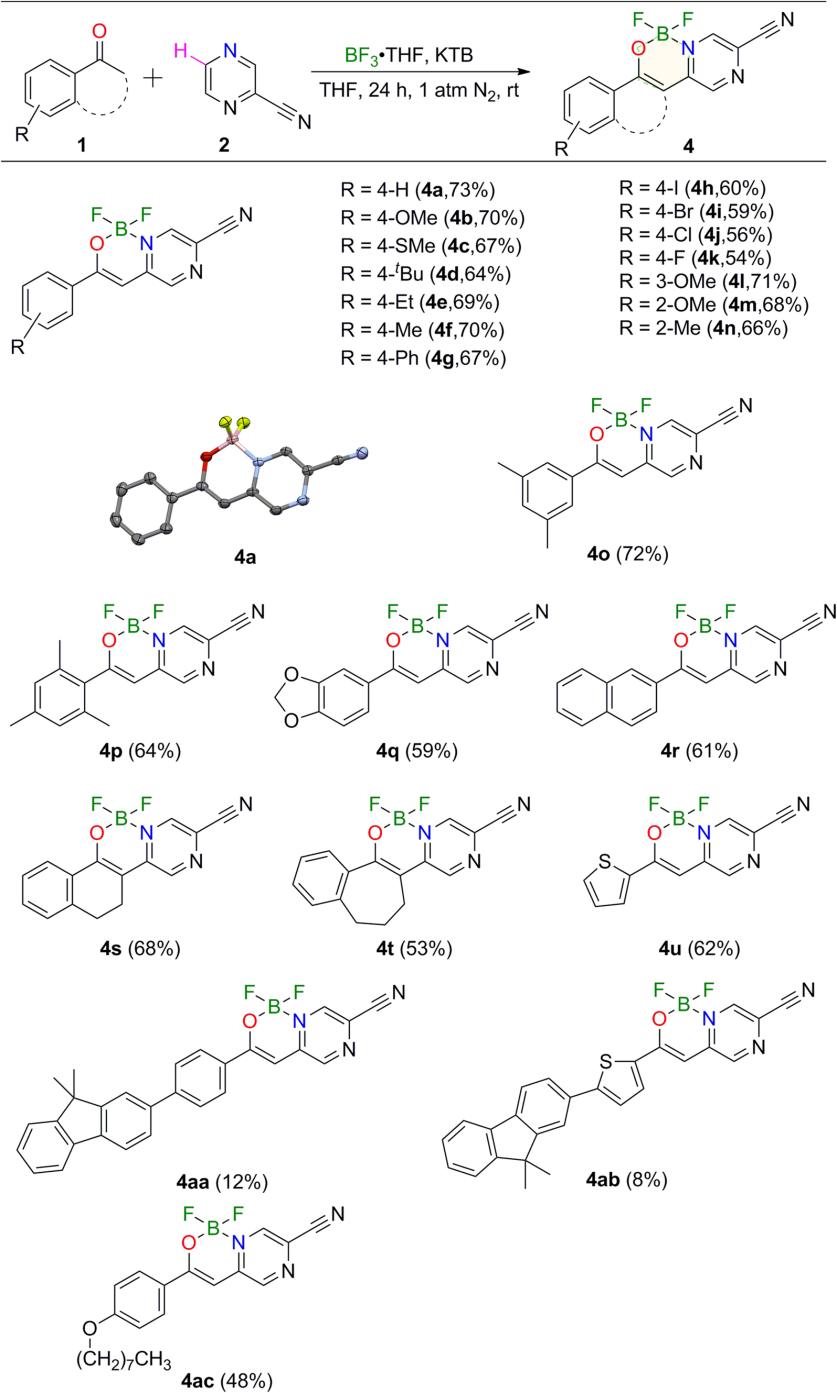
The scope of acetophenone derivatives used for the synthesis of compounds 4^*a,b*^. ^a^Reaction conditions: 1 (0.20 mmol), 2 (0.30 mmol), KTB (0.60 mmol), THF (2.0 mL), BF_3_∙THF (0.60 mmol), N_2_, rt, 24 h. ^b^Isolated yields

Acetophenone **1a** and 2-cyanopyrazine **2** were selected as model substrates to produce compound **4a** (Figure of Table 1). A mixture of acetophenone **1a** (0.20 mmol), 2-cyanopyrazine **2** (0.30 mmol), potassium t-butoxide (0.60 mmol) and boron trifluoride tetrahydrofuran (0.60 mmol) in THF (2.0 mL) was stirred in nitrogen atmosphere at room temperature for 24 h, at last providing compound **4a** in 73% yield based on acetophenone (Table 1, entry 1). The structure of **4a** was determined by X-ray crystallography (Scheme 2, **4a**). The crystal data of compounds **4a**, **4aa**, **4ab** and **5** are included in additional file 1, 2. Other alkali salts and/or organic base such as K_2_CO_3_, KOH and Et_3_N afforded the product in lower yields or no yield (entries 2–4). Higher temperature was a disadvantage to the reaction (entries 1, 5–7). If the temperature exceeds 100 ℃, no product will be obtained. The effect of solvent was found to be essential for the generation of the product (entries 1, 8–10).

**Table 1:** Optimization of the formation of **4a**^*a*^


Entry	Base	Temp (^o^C)	Solvent	Yields^b^
1	^***t***^**BuOK**	**rt**	**THF**	**73%**
2	**K**_**2**_**CO**_**3**_	rt	THF	None
3	**KOH**	rt	THF	None
4	**Et**_**3**_**N**	rt	THF	None
5	^*t*^BuOK	**60**	THF	31%
6	^*t*^BuOK	**80**	THF	10%
7	^*t*^BuOK	**100**	THF	trace
8	^*t*^BuOK	rt	**DCM**	10%
9	^*t*^BuOK	rt	**1,4-Dioxane**	trace
10	^*t*^BuOK	rt	**DMF**	None

^a^Reaction conditions: **1a** (0.20 mmol), **2** (0.30 mmol), base, solvent, BF_3_∙THF (0.60 mmol), N_2_. ^b^Yields were determined by ^1^H NMR analysis of the crude product using 1,3,5-trimethoxybenzene as the internal standard. Temp, Temperature

With the best conditions in hand, we sought to investigate the generality of the reaction (Scheme 2). The yields was discussed in terms of electronic effect and the steric effect of functional groups on the substrates. Firstly, experimental results showed that the electronic effect on the phenyl rings of **1** will affect the production of compound **4**. Substitution of the electron-donating groups or weak electron-withdrawing groups at the *para*-position of the phenyl group would bring about products in yields of 50–70% (**4a-4k**). Substitution of electron-donating groups at the *para*-position of the phenyl group had little influence on the reaction yield. The strong electron-donating group -OMe substituted product (**4b**) could be obtained in good yield. When the *para*-position of the phenyl group was electron withdrawing group, the situation was just the opposite. Substrates bearing weak electron-withdrawing groups could still react to give products **4h-4k** while the yield decreased in succession. However, the reaction was totally suppressed when strong electron-withdrawing groups such as − CF_3_, − NO_2_ and group -COOEt were introduced to the substrates. Secondly, the steric effect of substituents on the substrates was studied by employing methoxy- (**4b**, **4l**, **4m**) at the *para*, *meta*, and *ortho* positions of the phenyl group respectively. With the shift of the functional groups from the *para* to *ortho* positions, there was no obvious change in reaction yield. Moreover, substrates with two substituents at the 3,5 positions of phenyl (**4o**) or three substituents at the 2,4,6 positions of phenyl (**4p**) had little effect on the yield of the reaction. Some fused-ring and heterocyclic substrates were also tested, most of them providing corresponding compounds in good yields (45%-58%) (**4q**-**4u**). However, the yield of **4t** decreased significantly (45%). In addition, the synthetic utility of our reaction was also examined by running the experiments on gram scale. The reactions of acetophenone **1a** (9.0 mmol) with 2-cyanopyrazine **2** (13.5 mmol) in our system afforded the product **4a** in 46% yield (Additional file 3: Section 2.2).

**Scheme 3:**
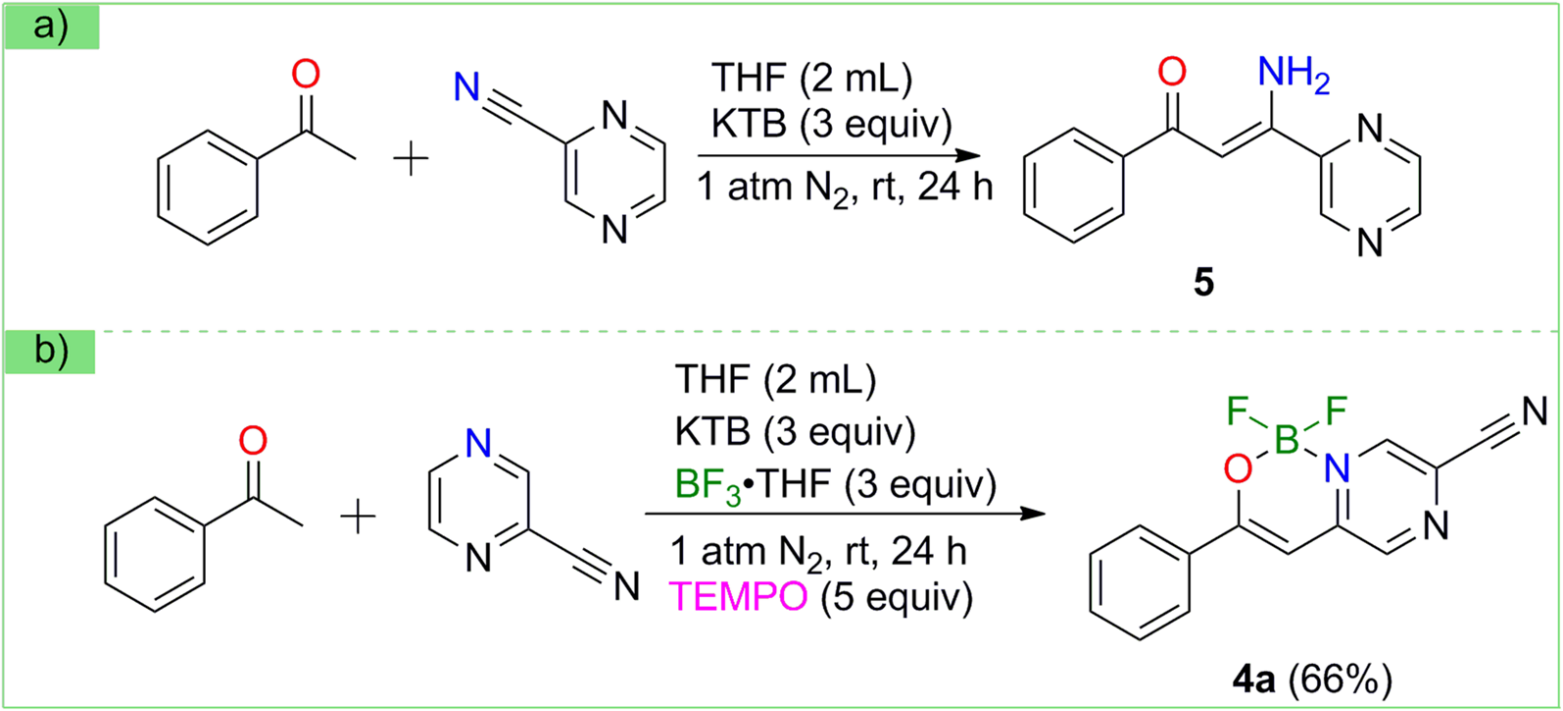
Control Experiments

**Scheme 4:**
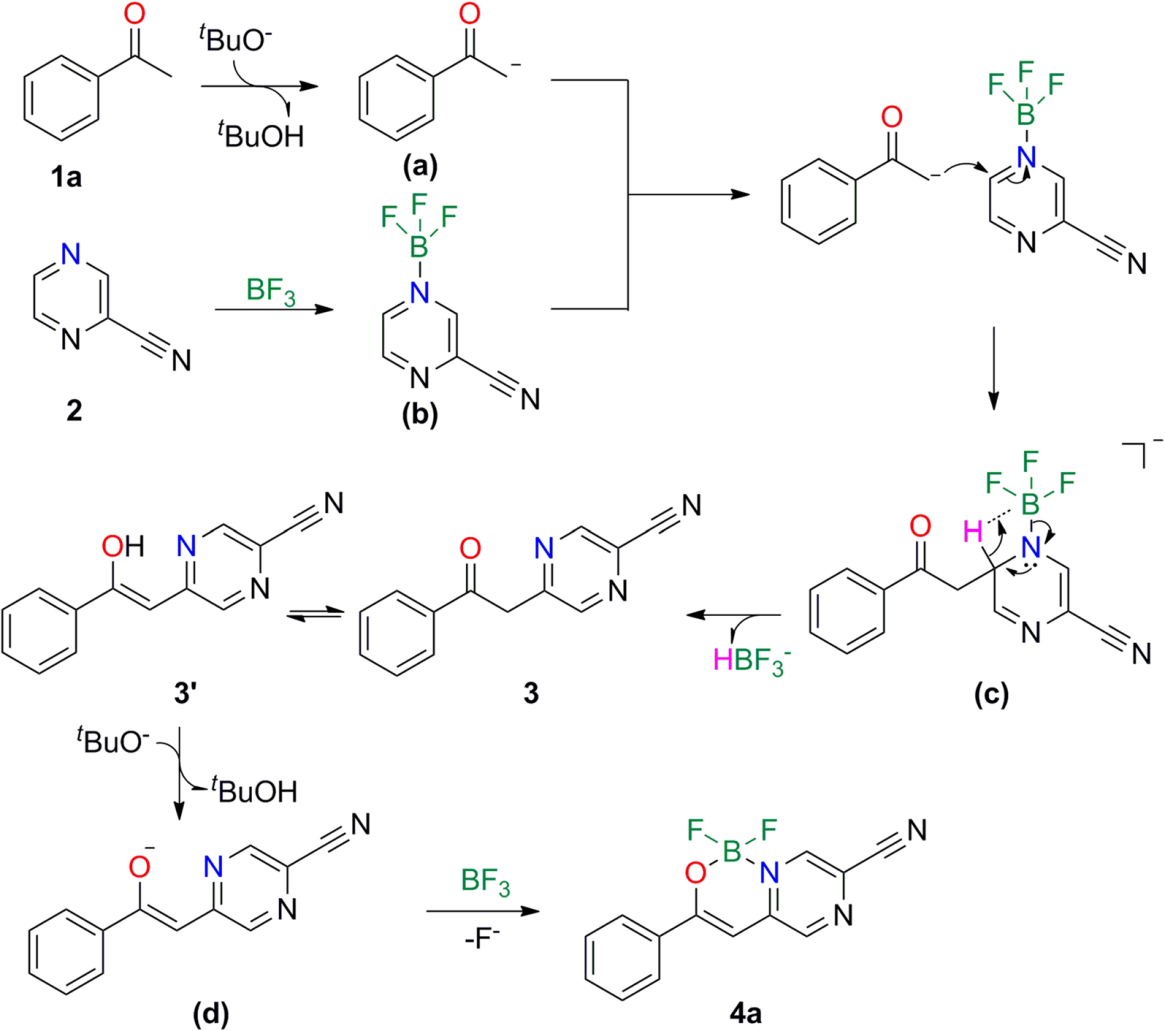
Proposed reaction mechanism for the generation of compound 4**a**

With the progress of building the N,O-bidentate organic BF_2_ motifs, several experiments were carried out for the investigation of the reaction mechanism (Scheme 3). In experiment shown in Scheme 3a, compound **5** was isolated instead of compound **3** or **3’** (Scheme 4) without the addition of boron trifluoride, indicating that C-H activation process was relevant to the use of boron trifluoride. When extra five equivalent free radical trapping agent (TEMPO) was added in standard conditions, the reaction proceeded effectively to afford **4a** in 66% yield (Scheme 3b), so we thought no free radical process was involved in the reaction pathways.

Based on the experimental results presented above, a possible reaction mechanism is proposed (Scheme 4). In the presence of base, acetophenone **1a** is deprotonated to form intermediate **(a)**. Reaction of 2-cyanopyrazine **2** with one equivalent boron trifluoride produces the intermediate **(b)**. After nucleophilic attack, intermediate **(a)** reacts with intermediate **(b)** to generate intermediate **(c)**. Intermediate **(c)** loses one molecule of HBF_3_^−^ by intermolecular electron transfer, then affording intermediate **3**. In solution, **3** is transformed into enol **3'** via keto-enol tautomerization. Finally, it **3**' immediately picks up another molecule of boron trifluoride to give target product **4a** in the presence of alkaline.

Compounds with D − π − A structure usually possess excellent luminescence property. Boron heterocycle is highly electron-deficient while -OMe at *para* position of phenyl in compound **4b** is an electron-donating group, which is favorable for the formation of the D − π − A system. The introduction of strong electron-donating group -OMe makes the compound **4b** exhibit excellent fluorescence performance. Fluorescence quantum yield of the compound **4b** was tested to be 79%. The fluorescent lifetime of it is 4.3 ns. Data of other compounds can be found in Additional file 3: Section 9.

**Fig. 2:**
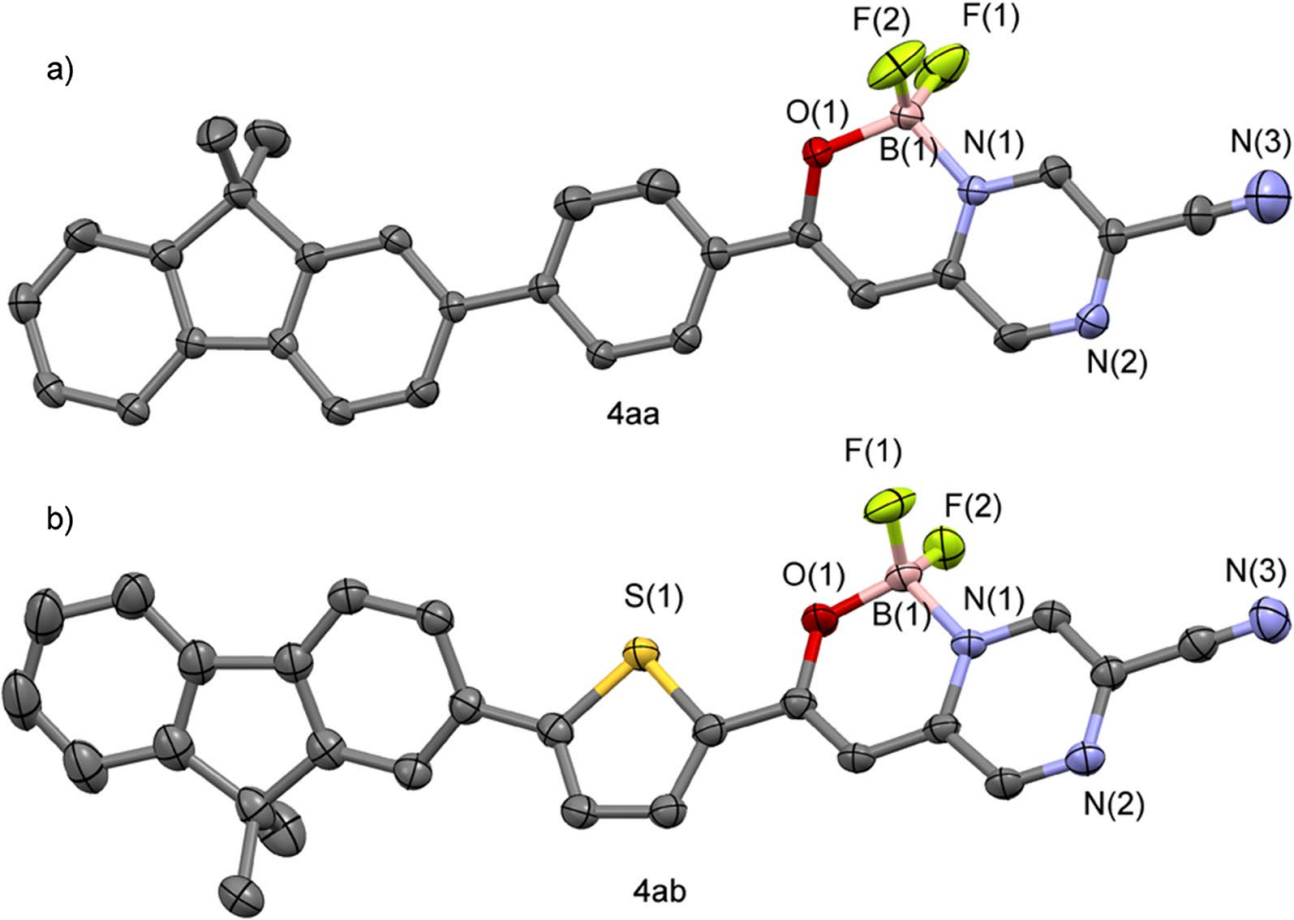
Crystal structures of compounds **4aa** and **4ab** with all non-hydrogen atoms shown as 50% probability ellipsoids

The extension of conjugation system in the fluorescent compound can reduce the energy gap between the highest occupied molecular orbital (HOMO) and the lowest unoccupied molecular orbital (LUMO), which leads to the red shift of emission wavelength. With that in mind, structural modification methods are discussed in order to obtain compounds with larger red shift. Fluorescence properties of compounds are usually enhanced by introducing fluorene. On the other hand, thiophene has distinctive electronic transmission capability [32, 33]. Therefore, it is common to introduce fluorene or thiophene ring to extend the conjugation system and improve the optical properties of compounds. Long-chain alkoxy group not only has stronger electron donating ability, but also can increase the solubility of compounds. According to the analysis, we designed and synthesized compounds **4aa, 4ab** and **4ac**. The structures of **4aa** and **4ab** were determined by X-ray crystallography (Fig. 2).

**Fig. 3:**
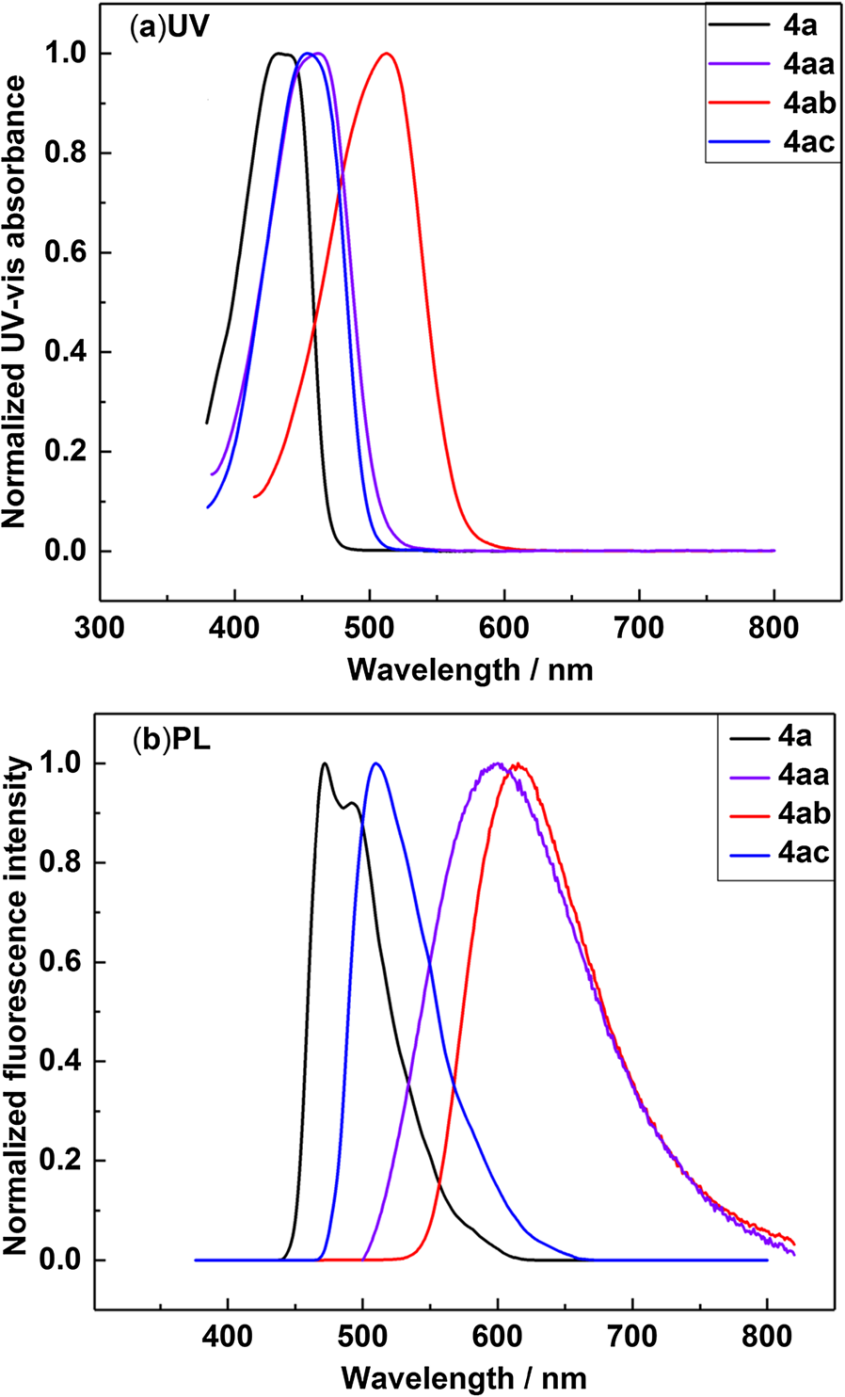
Absorption spectra and emission spectra of **4a**, **4aa**, **4ab** and **4ac** in dichloromethane at a concentration of 210^–5^ mol∙L^−1^

As shown in Fig. 3, the UV–vis and fluorescence spectra of representative N,O-bidentate organic BF_2_ complexes in dichloromethane were tested. The absorption and emission maxima of these BF_2_ complexes vary from 433 to 510 nm, and 472 nm to 615 nm respectively. Compounds **4a** and **4ac** exhibit good fluorescence properties (Table 2). Data of other compounds can be found in Additional file 3: Section 9.

**Table 2:** Optical Properties of 4a, 4aa, 4ab, and 4ac

Comp	λ_max_ (nm)	F_max_ (nm)	Stokes shifts (cm^−1^)	Φ_f_,%	τ_s_[ns]
4a	433	472	1908	75	3.5
4aa	462	600	4980	19	0.8
4ab	510	615	3350	19	0.8
4ac	455	510	2370	80	3.9

The extension of conjugation system in the fluorescent compound effectively makes compounds **4aa** and **4ab** red shifts. The solution-state fluorescence spectra of **4aa** and **4ab** exhibited larger Stokes shifts. The large Stokes shifts of fluorescent materials has the advantages of low background interference, small light damage to biological samples, strong sample penetrability and high detection sensitivity [34–36]. These compounds have potential for biological imaging.

## Conclusions

In summary, we have developed the diversity-oriented efficient one-pot synthesis of a series of a novel pyrazineboron complexes. Boron trifluoride and potassium t-butoxide induce C–H activation and difluoroboronation at room temperature. These compounds show excellent photophysical properties, including high fluorescence quantum yields in solution, large Stokes shifts and excellent stability. Further structural modification was carried out to improve the fluorescent properties of the products.

## Experimental

A mixture of acetophenone **1a** (0.20 mmol), 2-cyanopyrazine **2** (0.30 mmol), potassium t-butoxide (0.60 mmol) and boron trifluoride tetrahydrofuran (0.60 mmol) in THF (2.0 mL) was stirred in nitrogen atmosphere at room temperature for 24 h. After reaction, 10 mL water was added and the reaction mixture was exacted with dichloromethane (3×40 mL). Filtered through a pad of silica gel, and concentrated under reduced pressure. The crude product was purified on a silica gel column eluted with petroleum ether/dichloromethane (10:3 to absolute dichloromethane v/v) to afford the products **4a**-**4u**. **4aa**, **4ab** and **4ac** were obtained by the same method.

## Supplementary Information

**Additional file 1.** Checkcifs of the compounds 4a, 4aa, 4ab and 5.

**Additional file 2.** Cifs of the compounds 4a, 4aa, 4ab and 5.

**Additional file 3.** Supporting document showing the ^1^H NMR, ^13^C NMR, IR spectra and photophysical data of each compound studied in this paper.

## Data Availability

The data underlying this study are available in the published article and its online supplementary material. The Supporting Information is available free of charge via the Internet at 2217152, 2217156, 2217158 and 2217159 contains the supplementary crystallographic data for this paper. These data can be obtained free of charge via www.ccdc.cam.ac.uk/data_request/cif, or by emailing data_request@ccdc.cam.ac.uk, or by contacting The Cambridge Crystallographic Data Centre, 12 Union Road, Cambridge CB2 1EZ, UK; fax: + 44 1223 336033.
