# Supplementary material for: Synthesis of N,O-bidentate organic difluoroboron complexes and their photophysical studies
Source: BMC Chem. 2023 Jun 12;17(1):53. doi: 10.1186/s13065-023-00974-7 (PMC10259049; doi:10.1186/s13065-023-00974-7)

checkCIF/PLATON report

Structure factors have been supplied for datablock(s) 4a

THIS REPORT IS FOR GUIDANCE ONLY. IF USED AS PART OF A REVIEW PROCEDURE FOR PUBLICATION, IT SHOULD NOT REPLACE THE EXPERTISE OF AN EXPERIENCED CRYSTALLOGRAPHIC REFEREE.

No syntax errors found. CIF dictionary Interpreting this report

Datablock: 4a

Bond precision:	C-C = 0.0031 A		Wavelength=1.54184
Cell:	a=5.3416(2)	b=13.1660(4)	c=16.9223(7)
	alpha=90	beta=90	gamma=90
Temperature:	150 K		

	Calculated	Reported
Volume	1190.10(8)	1190.10(8)
Space group	P 21 21 21	P212121
Hall group	P 2ac 2ab	P 2ac 2ab
Moiety formula	C13 H8 B F2 N3 O	C13 H8 B F2 N3 O
Sum formula	C13 H8 B F2 N3 O	C13 H8 B F2 N3 O
Mr	271.03	271.03
Dx,g cm-3	1.513	1.513
Z	4	4
Mu (mm-1)	1.020	1.020
F000	552.0	552.0
F000'	554.01	
h,k,lmax	6,16,20	6,16,20
Nref	2269[1349]	2149
Tmin,Tmax	0.815,0.876	0.822,0.879
Tmin'	0.815	

Correction method= # Reported T Limits: Tmin=0.822 Tmax=0.879
AbsCorr = MULTI-SCAN

Data completeness= 1.59/0.95 Theta(max)= 69.950

R(reflections)= 0.0359(1960) wR2(reflections)= 0.0930(2149)

S = 1.052 Npar= 181

The following ALERTS were generated. Each ALERT has the format
test-name_ALERT_alert-type_alert-level.
Click on the hyperlinks for more details of the test.

Alert level C

```
STRVA01_ALERT_4_C           Flack test results are ambiguous.
           From the CIF: _refine_ls_abs_structure_Flack    0.670
           From the CIF: _refine_ls_abs_structure_Flack_su    0.170
PLAT907_ALERT_2_C Flack x > 0.5, Structure Needs to be Inverted? .       0.67 Check
PLAT911_ALERT_3_C Missing FCF Refl Between Thmin & STh/L=    0.600          2 Report
```

Alert level G

```
PLAT005_ALERT_5_G No Embedded Refinement Details Found  in the CIF     Please Do !
PLAT033_ALERT_4_G Flack x Value Deviates > 3.0 * sigma from Zero .      0.670 Note
PLAT066_ALERT_1_G Predicted and Reported Tmin&Tmax Range Identical          ? Check
PLAT093_ALERT_1_G No s.u.'s on H-positions, Refinement Reported as      mixed Check
PLAT710_ALERT_4_G Delete 1-2-3 or 2-3-4 Linear Torsion Angle ... #         32 Do !
              N2  -C11 -C13 -N3     37.00  5.00   1_555   1_555   1_555   1_555
PLAT710_ALERT_4_G Delete 1-2-3 or 2-3-4 Linear Torsion Angle ... #         33 Do !
              C12 -C11 -C13 -N3   -141.00  5.00   1_555   1_555   1_555   1_555
PLAT899_ALERT_4_G SHELXL97  is Deprecated and Succeeded by SHELXL/       2018 Note
PLAT912_ALERT_4_G Missing # of FCF Reflections Above STh/L=  0.600          6 Note
PLAT916_ALERT_2_G Hooft y and Flack x Parameter Values Differ by .       0.17 Check
PLAT961_ALERT_5_G Dataset Contains no Negative Intensities .......     Please Check
PLAT978_ALERT_2_G Number C-C Bonds with Positive Residual Density.          4 Info
```

0 **ALERT level A** = Most likely a serious problem - resolve or explain
0 **ALERT level B** = A potentially serious problem, consider carefully
3 **ALERT level C** = Check. Ensure it is not caused by an omission or oversight
11 **ALERT level G** = General information/check it is not something unexpected

2 ALERT type 1 CIF construction/syntax error, inconsistent or missing data
3 ALERT type 2 Indicator that the structure model may be wrong or deficient
1 ALERT type 3 Indicator that the structure quality may be low
6 ALERT type 4 Improvement, methodology, query or suggestion
2 ALERT type 5 Informative message, check

It is advisable to attempt to resolve as many as possible of the alerts in all categories. Often the minor alerts point to easily fixed oversights, errors and omissions in your CIF or refinement strategy, so attention to these fine details can be worthwhile. In order to resolve some of the more serious problems it may be necessary to carry out additional measurements or structure refinements. However, the purpose of your study may justify the reported deviations and the more serious of these should normally be commented upon in the discussion or experimental section of a paper or in the "special_details" fields of the CIF. checkCIF was carefully designed to identify outliers and unusual parameters, but every test has its limitations and alerts that are not important in a particular case may appear. Conversely, the absence of alerts does not guarantee there are no aspects of the results needing attention. It is up to the individual to critically assess their own results and, if necessary, seek expert advice.

Publication of your CIF in IUCr journals

A basic structural check has been run on your CIF. These basic checks will be run on all CIFs submitted for publication in IUCr journals (*Acta Crystallographica*, *Journal of Applied Crystallography*, *Journal of Synchrotron Radiation*); however, if you intend to submit to *Acta Crystallographica Section C* or *E* or *IUCrData*, you should make sure that full publication checks are run on the final version of your CIF prior to submission.

Publication of your CIF in other journals

Please refer to the *Notes for Authors* of the relevant journal for any special instructions relating to CIF submission.

PLATON version of 12/09/2022; check.def file version of 09/08/2022

Datablock 4a - ellipsoid plot

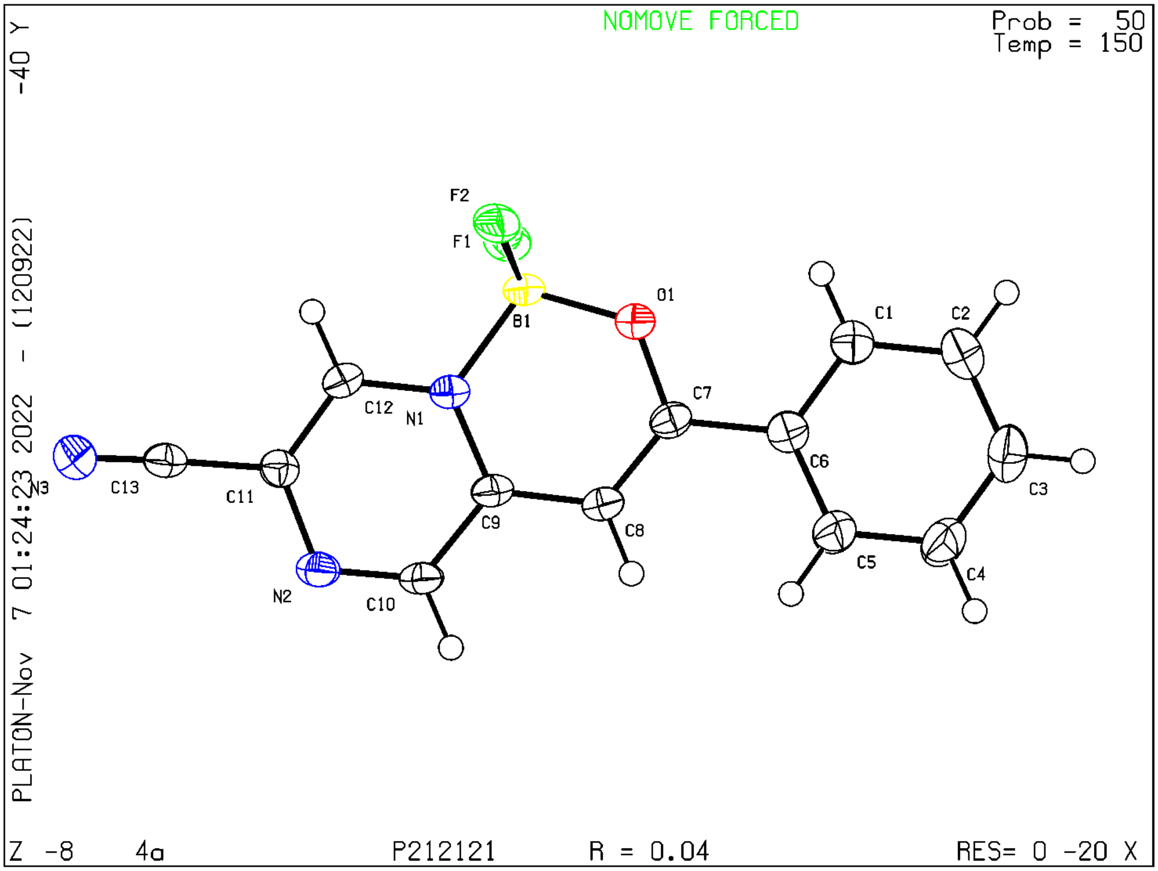

checkCIF/PLATON report

Structure factors have been supplied for datablock(s) 4aa

THIS REPORT IS FOR GUIDANCE ONLY. IF USED AS PART OF A REVIEW PROCEDURE FOR PUBLICATION, IT SHOULD NOT REPLACE THE EXPERTISE OF AN EXPERIENCED CRYSTALLOGRAPHIC REFEREE.

No syntax errors found.   CIF dictionary   Interpreting this report

Datablock: 4aa

```
Bond precision:    C-C = 0.0078 A           Wavelength=1.54184

Cell:              a=8.6992(7)     b=6.9631(6)         c=18.3825(14)
                   alpha=90        beta=98.989(7)      gamma=90
Temperature:       130 K

                 Calculated                    Reported
Volume           1099.82(16)                   1099.81(15)
Space group      P 21/m                        P21/m
Hall group       -P 2yb                        -P 2yb
Moiety formula   C28 H20 B F2 N3 O             C28 H20 B F2 N3 O
Sum formula      C28 H20 B F2 N3 O             C28 H20 B F2 N3 O
Mr               463.28                        463.28
Dx,g cm-3        1.399                         1.399
Z                2                             2
Mu (mm-1)        0.798                         0.798
F000             480.0                         480.0
F000'            481.52
h,k,lmax         10,8,22                       10,8,22
Nref             2273                          2254
Tmin,Tmax        0.839,0.866                   0.844,0.870
Tmin'            0.839

Correction method= # Reported T Limits: Tmin=0.844 Tmax=0.870
AbsCorr = MULTI-SCAN

Data completeness= 0.992           Theta(max)= 69.990

R(reflections)= 0.0739( 1451)                          wR2(reflections)=
                                                       0.2089( 2254)
S = 1.069                    Npar= 208
```

The following ALERTS were generated. Each ALERT has the format
test-name_ALERT_alert-type_alert-level.
Click on the hyperlinks for more details of the test.

Alert level C

```
PLAT241_ALERT_2_C High   'MainMol' Ueq as Compared to Neighbors of        C10 Check
PLAT241_ALERT_2_C High   'MainMol' Ueq as Compared to Neighbors of        C20 Check
PLAT250_ALERT_2_C Large U3/U1 Ratio for Average U(i,j) Tensor ....        2.6 Note
PLAT340_ALERT_3_C Low Bond Precision on  C-C Bonds ..............     0.00777 Ang.
PLAT906_ALERT_3_C Large K Value in the Analysis of Variance ......      25.613 Check
PLAT906_ALERT_3_C Large K Value in the Analysis of Variance ......       4.239 Check
PLAT911_ALERT_3_C Missing FCF Refl Between Thmin & STh/L=    0.600          7 Report
```

Alert level G

```
PLAT003_ALERT_2_G Number of Uiso or Uij Restrained non-H Atoms ...          4 Report
PLAT005_ALERT_5_G No Embedded Refinement Details Found  in the CIF     Please Do !
PLAT066_ALERT_1_G Predicted and Reported Tmin&Tmax Range Identical          ? Check
PLAT093_ALERT_1_G No s.u.'s on H-positions, Refinement Reported as      mixed Check
PLAT230_ALERT_2_G Hirshfeld Test Diff for    N3       --C13      .        5.2 s.u.
PLAT300_ALERT_4_G Atom Site Occupancy of O1         Constrained at        0.5 Check
PLAT301_ALERT_3_G Main Residue  Disorder ..............(Resd  1  )         3% Note
PLAT710_ALERT_4_G Delete 1-2-3 or 2-3-4 Linear Torsion Angle ... #         40 Do !
              N2  -C11 -C13 -N3    180.00  0.14    1_555   1_555   1_555   1_555
PLAT710_ALERT_4_G Delete 1-2-3 or 2-3-4 Linear Torsion Angle ... #         41 Do !
              C12 -C11 -C13 -N3      0.00  0.14    1_555   1_555   1_555   1_555
PLAT764_ALERT_4_G Overcomplete CIF Bond List Detected (Rep/Expd) .       1.10 Ratio
PLAT779_ALERT_4_G Suspect or Irrelevant (Bond) Angle(s) in CIF ...      37.60 Deg.
              O1   -C7   -O1      4_565   1_555   1_555 ......     #   28 Check
PLAT779_ALERT_4_G Suspect or Irrelevant (Bond) Angle(s) in CIF ...      34.10 Deg.
              O1   -B1   -O1      1_555   1_555   4_565 ......     #  101 Check
PLAT860_ALERT_3_G Number of Least-Squares Restraints ............          24 Note
PLAT899_ALERT_4_G SHELXL97  is Deprecated and Succeeded by SHELXL/       2018 Note
PLAT910_ALERT_3_G Missing # of FCF Reflection(s) Below Theta(Min).          1 Note
PLAT912_ALERT_4_G Missing # of FCF Reflections Above STh/L=  0.600         12 Note
PLAT961_ALERT_5_G Dataset Contains no Negative Intensities .......     Please Check
PLAT978_ALERT_2_G Number C-C Bonds with Positive Residual Density.          3 Info
```

0 **ALERT level A** = Most likely a serious problem - resolve or explain
0 **ALERT level B** = A potentially serious problem, consider carefully
7 **ALERT level C** = Check. Ensure it is not caused by an omission or oversight
18 **ALERT level G** = General information/check it is not something unexpected

2 ALERT type 1 CIF construction/syntax error, inconsistent or missing data
6 ALERT type 2 Indicator that the structure model may be wrong or deficient
7 ALERT type 3 Indicator that the structure quality may be low
8 ALERT type 4 Improvement, methodology, query or suggestion
2 ALERT type 5 Informative message, check

It is advisable to attempt to resolve as many as possible of the alerts in all categories. Often the minor alerts point to easily fixed oversights, errors and omissions in your CIF or refinement strategy, so attention to these fine details can be worthwhile. In order to resolve some of the more serious problems it may be necessary to carry out additional measurements or structure refinements. However, the purpose of your study may justify the reported deviations and the more serious of these should normally be commented upon in the discussion or experimental section of a paper or in the "special_details" fields of the CIF. checkCIF was carefully designed to identify outliers and unusual parameters, but every test has its limitations and alerts that are not important in a particular case may appear. Conversely, the absence of alerts does not guarantee there are no aspects of the results needing attention. It is up to the individual to critically assess their own results and, if necessary, seek expert advice.

Publication of your CIF in IUCr journals

A basic structural check has been run on your CIF. These basic checks will be run on all CIFs submitted for publication in IUCr journals (*Acta Crystallographica*, *Journal of Applied Crystallography*, *Journal of Synchrotron Radiation*); however, if you intend to submit to *Acta Crystallographica Section C* or *E* or *IUCrData*, you should make sure that full publication checks are run on the final version of your CIF prior to submission.

Publication of your CIF in other journals

Please refer to the *Notes for Authors* of the relevant journal for any special instructions relating to CIF submission.

PLATON version of 12/09/2022; check.def file version of 09/08/2022

Datablock 4aa - ellipsoid plot

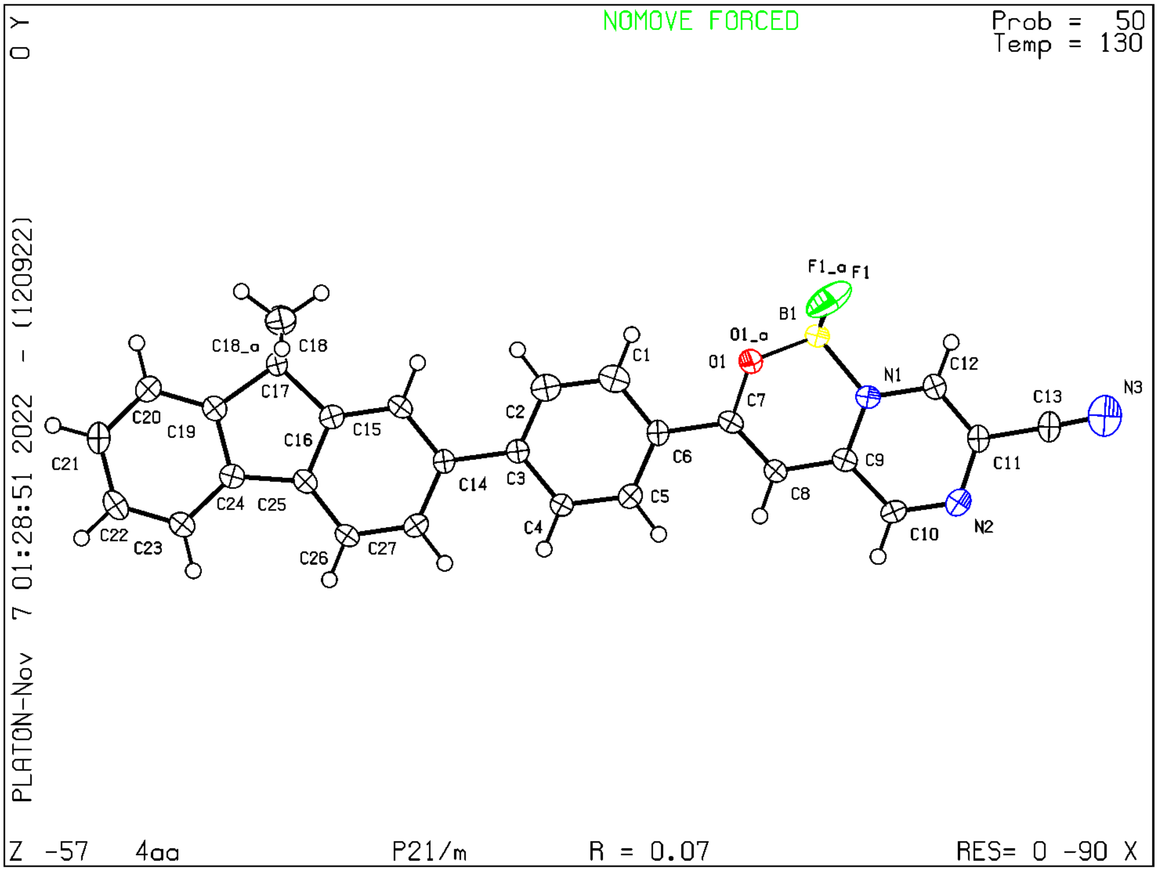

checkCIF/PLATON report

Structure factors have been supplied for datablock(s) 4ab

THIS REPORT IS FOR GUIDANCE ONLY. IF USED AS PART OF A REVIEW PROCEDURE FOR PUBLICATION, IT SHOULD NOT REPLACE THE EXPERTISE OF AN EXPERIENCED CRYSTALLOGRAPHIC REFEREE.

No syntax errors found. CIF dictionary Interpreting this report

Datablock: 4ab

Bond precision:	C-C = 0.0041 A		Wavelength=1.54184
Cell:	a=17.5431(4)	b=12.9697(2)	c=12.1624(2)
	alpha=90	beta=99.290(2)	gamma=90
Temperature:	157 K		

	Calculated	Reported
Volume	2731.00(9)	2730.99(9)
Space group	P 21/c	P21/c
Hall group	-P 2ybc	-P 2ybc
Moiety formula	C26 H18 B F2 N3 O S, C H Cl3	C26 H18 B F2 N3 O S, C H Cl3
Sum formula	C27 H19 B Cl3 F2 N3 O S	C27 H19 B Cl3 F2 N3 O S
Mr	588.67	588.67
Dx,g cm-3	1.432	1.432
Z	4	4
Mu (mm-1)	4.100	4.100
F000	1200.0	1200.0
F000'	1208.74	
h,k,lmax	21,15,14	21,15,14
Nref	5179	5174
Tmin,Tmax	0.502,0.960	0.494,0.956
Tmin'	0.419	

Correction method= # Reported T Limits: Tmin=0.494 Tmax=0.956
AbsCorr = MULTI-SCAN

Data completeness= 0.999 Theta(max)= 69.990

R(reflections)= 0.0579(4268) wR2(reflections)= 0.1646(5174)

S = 1.024 Npar= 345

The following ALERTS were generated. Each ALERT has the format
test-name_ALERT_alert-type_alert-level.
Click on the hyperlinks for more details of the test.

Alert level C

```
PLAT340_ALERT_3_C Low Bond Precision on  C-C Bonds ...............    0.00407 Ang.
PLAT906_ALERT_3_C Large K Value in the Analysis of Variance ......      2.883 Check
```

Alert level G

```
PLAT005_ALERT_5_G No Embedded Refinement Details Found  in the CIF     Please Do !
PLAT093_ALERT_1_G No s.u.'s on H-positions, Refinement Reported as      mixed Check
PLAT710_ALERT_4_G Delete 1-2-3 or 2-3-4 Linear Torsion Angle ... #         33 Do !
              N2  -C9  -C11 -N3    126.00  9.00   1_555   1_555   1_555   1_555
PLAT710_ALERT_4_G Delete 1-2-3 or 2-3-4 Linear Torsion Angle ... #         34 Do !
              C10 -C9  -C11 -N3    -52.00  9.00   1_555   1_555   1_555   1_555
PLAT790_ALERT_4_G Centre of Gravity not Within Unit Cell: Resd.  #          2 Note
              C H Cl3
PLAT899_ALERT_4_G SHELXL97  is Deprecated and Succeeded by SHELXL/       2018 Note
PLAT910_ALERT_3_G Missing # of FCF Reflection(s) Below Theta(Min).          1 Note
PLAT912_ALERT_4_G Missing # of FCF Reflections Above STh/L=  0.600          4 Note
PLAT961_ALERT_5_G Dataset Contains no Negative Intensities .......     Please Check
PLAT978_ALERT_2_G Number C-C Bonds with Positive Residual Density.          7 Info
```

0 **ALERT level A** = Most likely a serious problem - resolve or explain
0 **ALERT level B** = A potentially serious problem, consider carefully
2 **ALERT level C** = Check. Ensure it is not caused by an omission or oversight
10 **ALERT level G** = General information/check it is not something unexpected

1 ALERT type 1 CIF construction/syntax error, inconsistent or missing data
1 ALERT type 2 Indicator that the structure model may be wrong or deficient
3 ALERT type 3 Indicator that the structure quality may be low
5 ALERT type 4 Improvement, methodology, query or suggestion
2 ALERT type 5 Informative message, check

It is advisable to attempt to resolve as many as possible of the alerts in all categories. Often the minor alerts point to easily fixed oversights, errors and omissions in your CIF or refinement strategy, so attention to these fine details can be worthwhile. In order to resolve some of the more serious problems it may be necessary to carry out additional measurements or structure refinements. However, the purpose of your study may justify the reported deviations and the more serious of these should normally be commented upon in the discussion or experimental section of a paper or in the "special_details" fields of the CIF. checkCIF was carefully designed to identify outliers and unusual parameters, but every test has its limitations and alerts that are not important in a particular case may appear. Conversely, the absence of alerts does not guarantee there are no aspects of the results needing attention. It is up to the individual to critically assess their own results and, if necessary, seek expert advice.

Publication of your CIF in IUCr journals

A basic structural check has been run on your CIF. These basic checks will be run on all CIFs submitted for publication in IUCr journals (*Acta Crystallographica*, *Journal of Applied Crystallography*, *Journal of Synchrotron Radiation*); however, if you intend to submit to *Acta Crystallographica Section C* or *E* or *IUCrData*, you should make sure that full publication checks are run on the final version of your CIF prior to submission.

Publication of your CIF in other journals

Please refer to the *Notes for Authors* of the relevant journal for any special instructions relating to CIF submission.

PLATON version of 12/09/2022; check.def file version of 09/08/2022

Datablock 4ab - ellipsoid plot

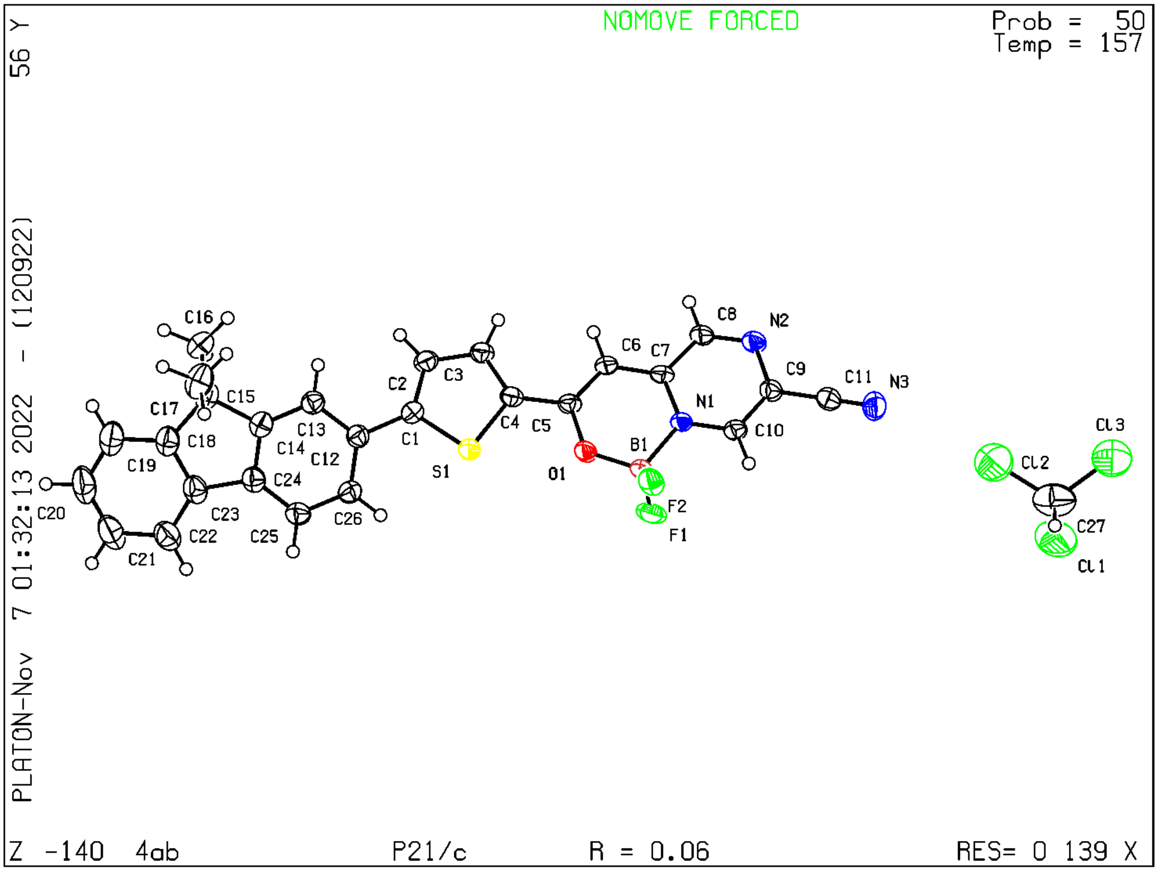

checkCIF/PLATON report

Structure factors have been supplied for datablock(s) 5

THIS REPORT IS FOR GUIDANCE ONLY. IF USED AS PART OF A REVIEW PROCEDURE FOR PUBLICATION, IT SHOULD NOT REPLACE THE EXPERTISE OF AN EXPERIENCED CRYSTALLOGRAPHIC REFEREE.

No syntax errors found. CIF dictionary Interpreting this report

Datablock: 5

```
Bond precision:   C-C = 0.0042 A          Wavelength=1.54184

Cell:              a=6.9059(7)     b=11.6510(6)      c=14.2914(12)
                   alpha=90        beta=90           gamma=90
Temperature:       280 K

                  Calculated                  Reported
Volume            1149.90(16)                 1149.90(16)
Space group       P 21 21 21                  P212121
Hall group        P 2ac 2ab                   P 2ac 2ab
Moiety formula    C13 H11 N3 O                C13 H11 N3 O
Sum formula       C13 H11 N3 O                C13 H11 N3 O
Mr                225.25                      225.25
Dx,g cm-3         1.301                       1.301
Z                 4                           4
Mu (mm-1)         0.695                       0.695
F000              472.0                       472.0
F000'             473.40
h,k,lmax          8,14,17                     8,14,17
Nref              2185[ 1286]                 2139
Tmin,Tmax         0.861,0.914                 0.856,0.915
Tmin'             0.852

Correction method= # Reported T Limits: Tmin=0.856 Tmax=0.915
AbsCorr = NONE

Data completeness= 1.66/0.98       Theta(max)= 69.970

R(reflections)= 0.0496( 1627)                        wR2(reflections)=
                                                     0.1592( 2139)

S = 1.089                   Npar= 155
```

The following ALERTS were generated. Each ALERT has the format
test-name_ALERT_alert-type_alert-level.
Click on the hyperlinks for more details of the test.

Alert level C

```
STRVA01_ALERT_4_C           Flack parameter is too small
           From the CIF: _refine_ls_abs_structure_Flack   -0.500
           From the CIF: _refine_ls_abs_structure_Flack_su    0.500
PLAT031_ALERT_4_C Refined Extinction Parameter Within Range of ...      2.529 Sigma
PLAT241_ALERT_2_C High   'MainMol' Ueq as Compared to Neighbors of         C4 Check
PLAT340_ALERT_3_C Low Bond Precision on  C-C Bonds ...............    0.00417 Ang.
PLAT790_ALERT_4_C Centre of Gravity not Within Unit Cell: Resd.  #          1 Note
              C13 H11 N3 O
PLAT911_ALERT_3_C Missing FCF Refl Between Thmin & STh/L=    0.600          5 Report
```

Alert level G

```
PLAT005_ALERT_5_G No Embedded Refinement Details Found  in the CIF     Please Do !
PLAT007_ALERT_5_G Number of Unrefined Donor-H Atoms .............          2 Report
PLAT032_ALERT_4_G Std. Uncertainty on Flack Parameter Value High .      0.500 Report
PLAT066_ALERT_1_G Predicted and Reported Tmin&Tmax Range Identical          ? Check
PLAT093_ALERT_1_G No s.u.'s on H-positions, Refinement Reported as      mixed Check
PLAT899_ALERT_4_G SHELXL97  is Deprecated and Succeeded by SHELXL/       2018 Note
PLAT912_ALERT_4_G Missing # of FCF Reflections Above STh/L=  0.600          5 Note
PLAT913_ALERT_3_G Missing # of Very Strong Reflections in FCF ....          2 Note
PLAT961_ALERT_5_G Dataset Contains no Negative Intensities .......     Please Check
PLAT978_ALERT_2_G Number C-C Bonds with Positive Residual Density.          0 Info
```

0 **ALERT level A** = Most likely a serious problem - resolve or explain
0 **ALERT level B** = A potentially serious problem, consider carefully
6 **ALERT level C** = Check. Ensure it is not caused by an omission or oversight
10 **ALERT level G** = General information/check it is not something unexpected

2 ALERT type 1 CIF construction/syntax error, inconsistent or missing data
2 ALERT type 2 Indicator that the structure model may be wrong or deficient
3 ALERT type 3 Indicator that the structure quality may be low
6 ALERT type 4 Improvement, methodology, query or suggestion
3 ALERT type 5 Informative message, check

It is advisable to attempt to resolve as many as possible of the alerts in all categories. Often the minor alerts point to easily fixed oversights, errors and omissions in your CIF or refinement strategy, so attention to these fine details can be worthwhile. In order to resolve some of the more serious problems it may be necessary to carry out additional measurements or structure refinements. However, the purpose of your study may justify the reported deviations and the more serious of these should normally be commented upon in the discussion or experimental section of a paper or in the "special_details" fields of the CIF. checkCIF was carefully designed to identify outliers and unusual parameters, but every test has its limitations and alerts that are not important in a particular case may appear. Conversely, the absence of alerts does not guarantee there are no aspects of the results needing attention. It is up to the individual to critically assess their own results and, if necessary, seek expert advice.

Publication of your CIF in IUCr journals

A basic structural check has been run on your CIF. These basic checks will be run on all CIFs submitted for publication in IUCr journals (*Acta Crystallographica*, *Journal of Applied Crystallography*, *Journal of Synchrotron Radiation*); however, if you intend to submit to *Acta Crystallographica Section C* or *E* or *IUCrData*, you should make sure that full publication checks are run on the final version of your CIF prior to submission.

Publication of your CIF in other journals

Please refer to the *Notes for Authors* of the relevant journal for any special instructions relating to CIF submission.

PLATON version of 12/09/2022; check.def file version of 09/08/2022

Datablock 5 - ellipsoid plot

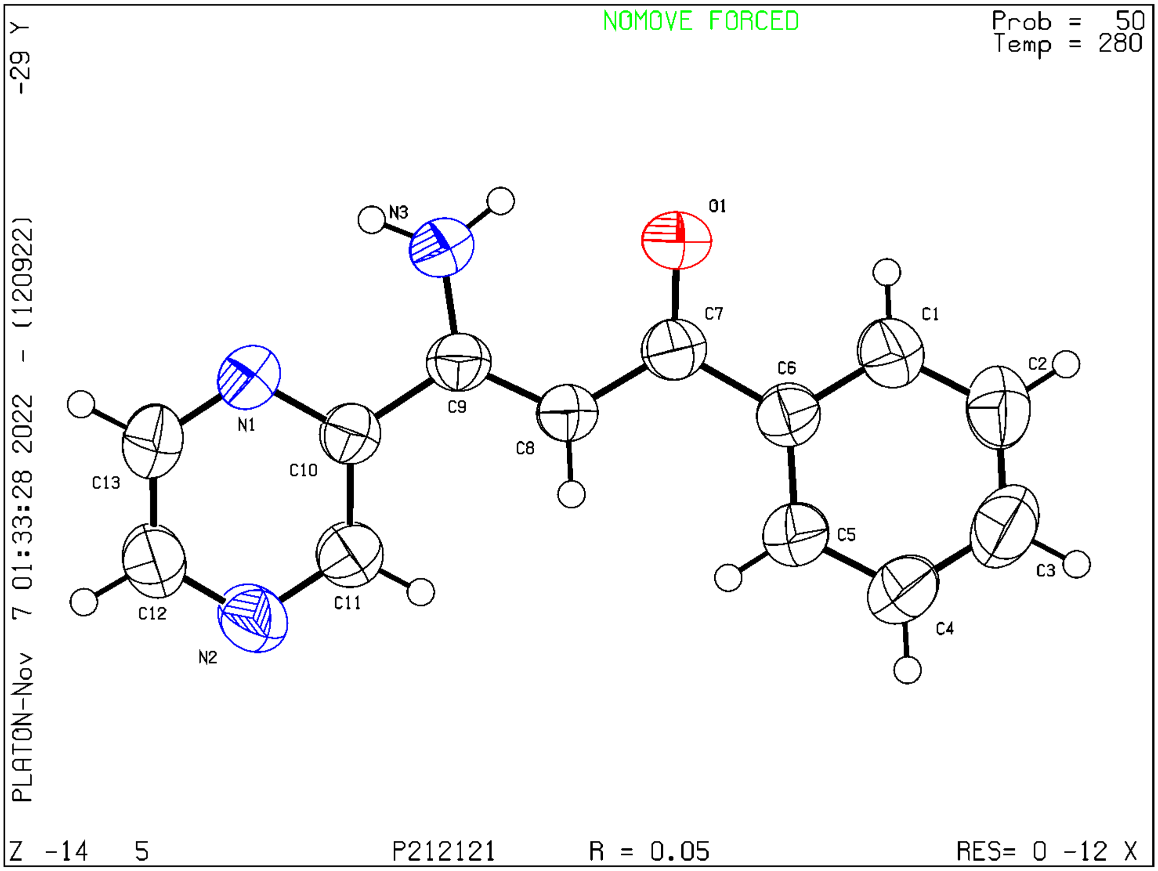

Supplement: Supplementary file 1 — Additional file 1. Checkcifs of the compounds 4a, 4aa, 4ab and 5. [file 13065_2023_974_MOESM1_ESM.pdf]
