# Supplementary material for: Synthesis of N,O-bidentate organic difluoroboron complexes and their photophysical studies
Source: BMC Chem. 2023 Jun 12;17(1):53. doi: 10.1186/s13065-023-00974-7 (PMC10259049; doi:10.1186/s13065-023-00974-7)
Supplement: Supplementary file 3 — Additional file 3. Supporting document showing the 1H NMR, 13C NMR, IR spectra and photophysical data of each compound studied in this paper. [file 13065_2023_974_MOESM3_ESM.docx]

**Additional file**

Synthesis of N,O-Bidentate Organic Difluoroboron Complexes and Their Photophysical Studies

Jin Guang,^a,b^ Weibin Fan,^a^ Zhiqi Liu^a^ and Deguang Huang*^,a^

*^a^State Key Laboratory of Structural Chemistry, Fujian Institute of Research on the Structure of Matter, Chinese Academy of Sciences, Fuzhou, Fujian 350002, China*

*^b^University of Chinese Academy of Sciences, Beijing 100049, China*

**Corresponding Author: Deguang Huang, E-mail: dhuang@fjirsm.ac.cn*

| **Table of contents:** |  |
| --- | --- |
| 1. General Information·················································································· | S2 |
| 2. Experimental procedure············································································· | S3 |
| 3. Characterization Data for the Products···························································· | S6 |
| 4. X-ray Structure Determinations···································································· | S14 |
| 5. Crystallographic data of compounds······························································· | S15 |
| 6. ^1^H NMR and ^13^C NMR spectra of compounds··················································· | S16 |
| 7. IR spectrum of compound **4a**······································································· | S44 |
| 8. Excitation-Emission-Matrix spectra of compound **4a**··········································· | S45 |
| 9. UV absorption and fluorescence data of compound **4** series molecules······················ | S46 |
| 10. References··························································································· | S51 |

**1. General Information**

**Chemicals.** Unless otherwise stated, commercial grade chemicals were used without further purification. Tetrahydrofuran were distilled over sodium under N_2_. Volume reduction and drying steps were performed in vacuo. The starting material 1-(4-(9,9-dimethyl-9H-fluoren-2-yl)phenyl) ethanone (**4a_1_**) and 1-(5-(9,9-dimethyl-9H-fluoren-2-yl) ethanone (**4a_2_**) for the synthesis of compound **4aa** and **4ab** were synthesized by the Suzuki Coupling reaction. The starting material 1-(4-(octyloxy)phenyl)ethanone (**4a_3_**) for the synthesis of compound **4ac** was synthesized according to literature method

**General Physical Measurements.** ^1^H NMR spectra were recorded on Bruker Avance III (400 MHz) and chemical shifts were expressed in *δ* ppm values with reference to tetramethylsilane (TMS) as internal standard. HR-MS (ESI) spectra were obtained using a Bruker Impact II quardrupole time off light mass spectrometer. The single crystal data were collected on an Oxford Diffraction Supernova dual diffractometer equipped with an Oxford Cryostream 700 low-temperature apparatus. UV-Vis spectra are recorded with a Lambda365 (190-1100 nm) ultraviolet spectrophotometer. Fluorescence spectra are recorded with a FLS1000 Spectrometer. IR spectrum is recorded with Bruker Vertex 70.

**2. Experimental procedure**

**2.1 General experimental procedure for the synthesis of N,O-bidentate BF_2_ complexes.**

A mixture of acetophenone **1a** (0.20 mmol), 2-cyanopyrazine **2** (0.30 mmol), potassium t-butoxide (0.60 mmol) and boron trifluoride tetrahydrofuran (0.60 mmol) in THF (2.0 mL) was stirred in nitrogen atmosphere at room temperature for 24 h. After reaction, 10 mL water was added and the reaction mixture was exacted with dichloromethane (3🞨40 mL). Filtered through a pad of silica gel, and concentrated under reduced pressure. The crude product was purified on a silica gel column eluted with petroleum ether/dichloromethane (10:3 to absolute dichloromethane v/v) to afford the products **4a**-**4u**. **4aa**, **4ab** and **4ac** were obtained by the same method.

**2.2 Experimental procedure for the scale-up reactions.**

A mixture of acetophenone **1a** (9.0 mmol, 1.08 g), 2-cyanopyrazine **2** (13.5 mmol, 1.41 g), potassium t-butoxide (27.0 mmol, 3.0g) and boron trifluoride tetrahydrofuran (27.0 mmol) in THF (50.0 mL) was stirred in nitrogen atmosphere at room temperature for 24 h. After reaction, 30 mL water was added and the reaction mixture was exacted with dichloromethane (3🞨100 mL). Filtered through a pad of silica gel, and concentrated under reduced pressure. The crude product was purified on a silica gel column eluted with petroleum ether /dichloromethane (10:3 to absolute dichloromethane v/v) to afford the product **4a** in yield 46% (1.12 g).

**2.3 Experimental procedure for the reactions shown in the Scheme 3.**

a) A mixture of acetophenone **1a** (0.20 mmol), 2-cyanopyrazine **2** (0.30 mmol) and potassium t-butoxide (0.60 mmol) in THF (2.0 mL) was stirred in nitrogen atmosphere at room temperature for 24 h. The reaction mixture was diluted with dichloromethane (10 mL). Filtered through a pad of silica gel, and concentrated under reduced pressure. The crude product was purified on a silica gel column eluted with petroleum ether/ethyl acetate (10:1 to 1:1 v/v) to afford the product **5**.

b) A mixture of acetophenone **1a** (0.20 mmol), 2-cyanopyrazine **2** (0.30 mmol), potassium t-butoxide (0.60 mmol), boron trifluoride tetrahydrofuran (0.60 mmol) and TEMPO (1.0 mmol) in THF (2.0 mL) was stirred in nitrogen atmosphere at room temperature for 24 h. After reaction, 10 mL water was added and the reaction mixture was exacted with dichloromethane (3🞨40 mL). Filtered through a pad of silica gel, and concentrated under reduced pressure. The crude product was purified on a silica gel column eluted with petroleum ether/dichloromethane (10:3 to absolute dichloromethane v/v) to afford the product **4a** in yield 66% (35.7 mg).

**2.4 Experimental procedure for the reactions of synthetic raw materials 4a_1_ and 4a_2_ in the Scheme S1, 4a_3_ in the Scheme S2.**

**Scheme S1. Reactions of synthetic raw materials 4a_1_ and 4a_2_**

a) A mixture of 4-bromoacetophenone (0.2 mmol, 39.8mg), 9,9-dimethyl-9H-fluoren-2-yl-boronic acid (0.2 mmol, 47.6 mg), tetrakis (triphenylphosphine) palladium (0.02 mmol, 23.1 mg) and potassium carbonate (1.0 mmol, 138.2 mg) in Chloroform (1.5 mL) and H_2_O (0.5 mL) was stirred at 100 ^o^C for 24 h in nitrogen. After cooling to room temperature, the reaction mixture was diluted with dichloromethane (10 mL), filtered through a pad of silica gel, and concentrated under reduced pressure. The crude product was purified on a silica gel column eluted with petroleum ether/ethyl acetate (15:1 to 3:1 v/v) to afford the product **4a_1._**

b) A mixture of 2-acetyl-5-bromothiophene (0.2 mmol, 41.0 mg), 9,9-dimethyl-9H-fluoren-2-yl-boronic acid (0.2 mmol, 47.6 mg), tetrakis (triphenylphosphine) palladium (0.02 mmol, 23.1 mg) and potassium carbonate (1.0 mmol, 138.2 mg) in Chloroform (1.5 mL) and H_2_O (0.5 mL) was stirred at 100 ^o^C for 24 h in nitrogen. After cooling to room temperature, the reaction mixture was diluted with dichloromethane (10 mL), filtered through a pad of silica gel, and concentrated under reduced pressure. The crude product was purified on a silica gel column eluted with petroleum ether/ethyl acetate (15:1 to 3:1 v/v) to afford the product **4a_2._**

**Scheme S2. Reactions of synthetic raw materials 4a_3_**

A mixture of 4'-hydroxyacetophenone (0.2 mmol, 27.2 mg), 1-bromooctane (0.3 mmol, 57.9 mg) and potassium carbonate (0.6 mmol, 82.9 mg) in N,N-Dimethylformamide (2 mL) was stirred at 80 ^o^C for 24 h. After cooling to room temperature, the reaction mixture was diluted with dichloromethane (10 mL), filtered through a pad of silica gel, and concentrated under reduced pressure. The crude product was purified on a silica gel column eluted with petroleum ether/ethyl acetate (60:1 to 5:1 v/v) to afford the product **4a_3_**.

**3. Characterization Data for the Products**

**7-cyano-1,1-difluoro-3-phenyl-1H-pyrazino[1,2-c][1,3,2]oxazaborinin-9-ium-1-uide (4a)**

Yield, 70% (37.5 mg); yellow solid; mp 210-212 ^o^C; ^1^H NMR (400 MHz, DMSO) δ 9.20 (s, 1H), 9.09 (s, 1H), 8.05 (d, *J* = 7.3 Hz, 2H), 7.67 (t, *J* = 7.2 Hz, 1H), 7.60 (t, *J* = 7.4 Hz, 2H), 7.28 (s, 1H). ^13^C NMR (101 MHz, DMSO) δ 168.9, 149.7, 147.1, 137.9, 133.5, 133.0, 129.7, 127.5, 124.3, 116.0, 93.4. HR-MS (ESI) *m/z* [M+Na]^+^ calcd for C_13_H_8_BF_2_N_3_ONa, 294.0626; found, 294.0621. IR neat 3091, 2237 cm^-1^.

**7-cyano-1,1-difluoro-3-(4-methoxyphenyl)-1H-pyrazino[1,2-c][1,3,2]oxazaborinin-9-ium-1-uide (4b)**

Yield, 65% (39.1 mg); yellow solid; mp 211-213 ^o^C; ^1^H NMR (400 MHz, DMSO) δ 9.07 (s, 1H), 8.97 (s, 1H), 8.01 (d, *J* = 8.9 Hz, 2H), 7.14 (s, 2H), 7.12 (s, 1H), 3.87 (s, 3H). ^13^C NMR (101 MHz, DMSO) δ 169.4, 163. 9, 149.6, 147.2, 137.8, 129.9, 125.1, 122.9, 116.1, 115.2, 92.1, 56.2. HR-MS (ESI) *m/z* [M+Na]^+^ calcd for C_14_H_10_BF_2_N_3_O_2_Na, 324.0732; found, 324.0737.

**7-cyano-1,1-difluoro-3-(4-(methylthio)phenyl)-1H-pyrazino[1,2-c][1,3,2]oxazaborinin-9-ium-1-uide (4c)**

Yield, 62% (39.3 mg); red solid; mp 230-231 ^o^C; ^1^H NMR (400 MHz, DMSO) δ 9.13 (s, 1H), 8.99 (s, 1H), 7.93 (d, *J* = 8.4 Hz, 2H), 7.40 (d, *J* = 8.4 Hz, 2H), 7.18 (s, 1H), 2.54 (s, 3H). ^13^C NMR (101 MHz, DMSO) δ 168.8, 149.7, 147.0, 146.4, 137.9, 128.7, 127.9, 125.8, 123.5, 116.0, 92.8, 14.4. HR-MS (ESI) *m/z* [M+Na]^+^ calcd for C_14_H_10_BF_2_N_3_OSNa, 340.0503; found, 340.0508.

**3-(4-(tert-butyl)phenyl)-7-cyano-1,1-difluoro-1H-pyrazino[1,2-c][1,3,2]oxazaborinin-9-ium-1-uide (4d)**

Yield, 60% (39.5 mg); yellow solid; mp 183-185 ^o^C; ^1^H NMR (400 MHz, DMSO) δ 9.15 (s, 1H), 9.06 (s, 1H), 7.97 (d, *J* = 8.5 Hz, 2H), 7.61 (d, *J* = 8.5 Hz, 2H), 7.23 (s, 1H), 1.32 (s, 9H). ^13^C NMR (101 MHz, DMSO) δ 169.1, 156.9, 149.7, 147.2, 137.9, 130.3, 127.5, 126.6, 123.8, 116.0, 93.0, 35.4, 31.2. HR-MS (ESI) *m/z* [M+Na]^+^ calcd for C_17_H_16_BF_2_N_3_ONa, 350.1252; found, 350.1251.

**7-cyano-3-(4-ethylphenyl)-1,1-difluoro-1H-pyrazino[1,2-c][1,3,2]oxazaborinin-9-ium-1-uide (4e)**

Yield, 64% (38.2 mg); yellow solid; mp 188-189 ^o^C; ^1^H NMR (400 MHz, DMSO) δ 9.13 (s, 1H), 9.03 (s, 1H), 7.95 (d, *J* = 7.8 Hz, 2H), 7.42 (d, *J* = 9.3 Hz, 2H), 7.21 (s, 1H), 2.69 (q, *J* = 7.6 Hz, 2H), 1.21 (t, *J* = 7.6 Hz, 3H). ^13^C NMR (101 MHz, DMSO) δ 169.3, 150.3, 149.6, 147.2, 137.9, 130.5, 129.1, 127.7, 123.7, 116.0, 92.9, 28.7, 15.5. HR-MS (ESI) *m/z* [M+Na]^+^ calcd for C_15_H_12_BF_2_N_3_ONa, 322.0939; found, 322.0945.

**7-cyano-1,1-difluoro-3-(p-tolyl)-1H-pyrazino[1,2-c][1,3,2]oxazaborinin-9-ium-1-uide (4f)**

Yield, 65% (36.8 mg); yellow solid; mp 214-216 ^o^C; ^1^H NMR (400 MHz, DMSO) δ 9.16 (s, 1H), 9.04 (s, 1H), 7.95 (d, *J* = 8.2 Hz, 2H), 7.41 (d, *J* = 8.0 Hz, 2H), 7.23 (s, 1H), 2.39 (s, 3H). ^13^C NMR (101 MHz, DMSO) δ 169.2, 149.7, 147.2, 144.3, 137.9, 130.4, 130.2, 127.6, 123.8, 116.1, 92.9, 21.7. HR-MS (ESI) *m/z* [M+Na]^+^ calcd for C_14_H_10_BF_2_N_3_ONa, 308.0783; found, 308.0788.

**3-([1,1'-biphenyl]-4-yl)-7-cyano-1,1-difluoro-1H-pyrazino[1,2-c][1,3,2]oxazaborinin-9-ium-1-uide (4g)**

Yield, 62% (43.0 mg); yellow solid; mp 252-253 ^o^C; ^1^H NMR (400 MHz, DMSO) δ 9.19 (s, 1H), 9.07 (s, 1H), 8.13 (d, *J* = 8.4 Hz, 2H), 7.92 (d, *J* = 8.4 Hz, 2H), 7.79 (d, *J* = 7.4 Hz, 2H), 7.52 (t, *J* = 7.5 Hz, 2H), 7.44 (t, *J* = 7.3 Hz, 1H), 7.33 (s, 1H). ^13^C NMR (101 MHz, DMSO) δ 168.5, 149.7, 147.1, 144.8, 139.05, 138.0, 131.8, 129.6, 129.1, 128.2, 127.8, 127.4, 124.1, 116.0, 93.5. HR-MS (ESI) *m/z* [M+Na]^+^ calcd for C_19_H_12_BF_2_N_3_ONa, 370.0939; found, 370.0946.

**7-cyano-1,1-difluoro-3-(4-iodophenyl)-1H-pyrazino[1,2-c][1,3,2]oxazaborinin-9-ium-1-uide (4h)**

Yield, 55% (43.6 mg); yellow solid; mp 241-243 ^o^C; ^1^H NMR (400 MHz, DMSO) δ 9.19 (s, 1H), 9.07 (s, 1H), 7.98 (d, *J* = 8.6 Hz, 2H), 7.79 (d, *J* = 8.6 Hz, 2H), 7.29 (s, 1H). ^13^C NMR (101 MHz, DMSO) δ 167.8, 149.7, 147.0, 138.6, 138.0, 132.4, 129.0, 128.4, 124.6, 115.9, 93.7. HR-MS (ESI) *m/z* [M+Na]^+^ calcd for C_13_H_7_BF_2_IN_3_ONa, 419.9593; found, 419.9588.

**3-(4-bromophenyl)-7-cyano-1,1-difluoro-1H-pyrazino[1,2-c][1,3,2]oxazaborinin-9-ium-1-uide (4i)**

Yield, 54% (37.8 mg); yellow solid; mp 232-235 ^o^C; ^1^H NMR (400 MHz, DMSO) δ 9.22 (s, 1H), 9.08 (s, 1H), 7.97 (d, *J* = 8.6 Hz, 2H), 7.83 (d, *J* = 15.7, 8.6 Hz, 2H), 7.31 (s, 1H). ^13^C NMR (101 MHz, DMSO) δ 167.4, 149.7, 147.0, 138.0, 132.8, 132.2, 129.3, 127.4, 124.7, 115.9, 93.8. HR-MS (ESI) *m/z* [M+Na]^+^ calcd for C_13_H_7_BrBF_2_N_3_ONa, 371.9731; found, 371.9727.

**3-(4-chlorophenyl)-7-cyano-1,1-difluoro-1H-pyrazino[1,2-c][1,3,2]oxazaborinin-9-ium-1-uide (4j)**

Yield, 51% (31.1 mg); yellow solid; mp 226-227 ^o^C; ^1^H NMR (400 MHz, DMSO) δ 9.21 (s, 1H), 9.06 (s, 1H), 8.03 (d, *J* = 10.6 Hz, 2H), 7.64 (d, *J* = 8.6 Hz, 2H), 7.28 (s, 1H). ^13^C NMR (101 MHz, DMSO) δ 167.3, 149.6, 147.0, 138.3, 138.0, 131.8, 129.8, 129.2, 124.6, 115.9, 93.7. HR-MS (ESI) *m/z* [M+Na]^+^ calcd for C_13_H_7_ClBF_2_N_3_ONa, 328.0236; found, 328.0231.

**7-cyano-1,1-difluoro-3-(4-fluorophenyl)-1H-pyrazino[1,2-c][1,3,2]oxazaborinin-9-ium-1-uide (4k)**

Yield, 50% (29.0 mg); yellow solid; mp 220-221 ^o^C; ^1^H NMR (400 MHz, DMSO) δ 9.20 (s, 1H), 9.06 (s, 1H), 8.12 (dd, *J* = 8.8, 5.4 Hz, 2H), 7.45 (t, *J* = 8.8 Hz, 2H), 7.27 (s, 1H). ^13^C NMR (101 MHz, DMSO) δ 167.7 (s), 165.4 (d, *J* = 252.4 Hz), 149.6 (s), 147.1 (s), 138.0 (s), 130.3 (d, *J* = 9.4 Hz), 129.5 (d, *J* = 2.7 Hz), 124.3 (s), 116.9 (d, *J* = 22.2 Hz), 116.0 (s), 93.3 (s). HR-MS (ESI) *m/z* [M+Na]^+^ calcd for C_13_H_7_BF_3_N_3_ONa, 312.0532; found, 312.0526.

**7-cyano-1,1-difluoro-3-(3-methoxyphenyl)-1H-pyrazino[1,2-c][1,3,2]oxazaborinin-9-ium-1-uide (4l)**

Yield, 66% (39.7 mg); yellow solid; mp 193-195 ^o^C; ^1^H NMR (400 MHz, DMSO) δ 9.19 (s, 1H), 9.06 (s, 1H), 7.63 (d, *J* = 7.9 Hz, 1H), 7.55 – 7.50 (m, 2H), 7.30 (s, 1H), 7.23 (d, *J* = 10.0 Hz, 1H), 3.86 (s, 3H). ^13^C NMR (101 MHz, DMSO) δ 168.6, 160.1, 149.6, 147.1, 138.0, 134.4, 130.9, 124.3, 119.9, 119.5, 116.0, 112.1, 93.7, 55.9. HR-MS (ESI) *m/z* [M+Na]^+^ calcd for C_14_H_10_BF_2_N_3_O_2_Na, 324.0732; found, 324.0736.

**7-cyano-1,1-difluoro-3-(2-methoxyphenyl)-1H-pyrazino[1,2-c][1,3,2]oxazaborinin-9-ium-1-uide (4m)**

Yield, 63% (38.0 mg); yellow solid; mp 218-220 ^o^C; ^1^H NMR (400 MHz, DMSO) δ 9.18 (s, 1H), 9.14 (s, 1H), 7.94 (d, *J* = 7.3 Hz, 1H), 7.58 (t, *J* = 22.0, 14.6 Hz, 1H), 7.37 (s, 1H), 7.24 (d, *J* = 8.3 Hz, 1H), 7.13 (t, *J* = 7.4 Hz, 1H), 3.92 (s, 3H). ^13^C NMR (101 MHz, DMSO) δ 166.4, 159.6, 150.3, 147.1, 137.7, 134.9, 129.8, 124.0, 121.4, 121.2, 116.0, 113.1, 98.1, 56.5. HR-MS (ESI) *m/z* [M+Na]^+^ calcd for C_14_H_10_BF_2_N_3_O_2_Na, 324.0732; found, 324.0735.

**7-cyano-1,1-difluoro-3-(o-tolyl)-1H-pyrazino[1,2-c][1,3,2]oxazaborinin-9-ium-1-uide (4n)**

Yield, 61% (34.8 mg); yellow solid; mp 142-144 ^o^C; ^1^H NMR (400 MHz, DMSO) δ 9.19 (s, 1H), 9.12 (s, 1H), 7.64 (d, *J* = 7.2 Hz, 1H), 7.48 (t, *J* = 7.5 Hz, 1H), 7.41 – 7.33 (m, 2H), 6.78 (s, 1H), 2.50 (s, 3H). ^13^C NMR (101 MHz, DMSO) δ 172.7, 149.6, 146.8, 137.8, 137.7, 134.1, 132.2, 132.0, 129.5, 126.8, 124.6, 115.9, 97.1, 21.0. HR-MS (ESI) *m/z* [M+Na]^+^ calcd for C_14_H_10_BF_2_N_3_ONa, 308.0783; found, 308.0786.

**7-cyano-3-(3,5-dimethylphenyl)-1,1-difluoro-1H-pyrazino[1,2-c][1,3,2]oxazaborinin-9-ium-1-uide (4o)**

Yield, 67% (40.1 mg); yellow solid; mp 253-255 ^o^C; ^1^H NMR (400 MHz, DMSO) δ 9.16 (s, 1H), 9.03 (s, 1H), 7.65 (s, 2H), 7.28 (s, 1H), 7.22 (s, 1H), 2.36 (s, 6H). ^13^C NMR (101 MHz, DMSO) δ 169.3, 149.6, 147.1, 139.0, 137.9, 135.1, 132.9, 125.2, 124.0, 116.0, 93.3, 21.3. HR-MS (ESI) *m/z* [M+Na]^+^ calcd for C_15_H_12_BF_2_N_3_ONa, 322.0939; found, 322.0932.

**7-cyano-1,1-difluoro-3-mesityl-1H-pyrazino[1,2-c][1,3,2]oxazaborinin-9-ium-1-uide (4p)**

Yield, 59% (36.8 mg); yellow solid; mp 177-179 ^o^C; ^1^H NMR (400 MHz, DMSO) δ 9.24 (s, 1H), 9.10 (s, 1H), 6.98 (s, 2H), 6.49 (s, 1H), 2.28 (s, 3H), 2.24 (s, 6H). ^13^C NMR (101 MHz, DMSO) δ 173.1, 149.3, 146.4, 139.7, 137.9, 136.0, 132.5, 128.9, 125.0, 115.9, 98.9, 21.2, 19.8. HR-MS (ESI) *m/z* [M+Na]^+^ calcd for C_16_H_14_BF_2_N_3_ONa, 336.1096; found, 336.1089.

**3-(benzo[d][1,3]dioxol-5-yl)-7-cyano-1,1-difluoro-1H-pyrazino[1,2-c][1,3,2]oxazaborinin-9-ium-1-uide (4q)**

Yield, 54% (34.0 mg); yellow solid; mp 232-235 ^o^C; ^1^H NMR (400 MHz, DMSO) δ 9.08 (s, 1H), 8.94 (s, 1H), 7.66 (d, *J* = 9.7 Hz, 1H), 7.53 (s, 1H), 7.14 (s, 1H), 7.11 (d, *J* = 8.3 Hz, 1H), 6.18 (s, 2H). ^13^C NMR (101 MHz, DMSO) δ 168.8, 152.2, 149.6, 148.7, 147.1, 137.8, 126.9, 123.9, 123.2, 116.1, 109.3, 106.9, 102.9, 92.6. HR-MS (ESI) *m/z* [M+Na]^+^ calcd for C_14_H_8_BF_2_N_3_O_3_Na, 338.0525; found, 338.0531.

**7-cyano-1,1-difluoro-3-(naphthalen-2-yl)-1H-pyrazino[1,2-c][1,3,2]oxazaborinin-9-ium-1-uide (4r)**

Yield, 56% (36.0 mg); yellow solid; mp 216-218 ^o^C; ^1^H NMR (400 MHz, DMSO) δ 9.19 (s, 1H), 9.07 (s, 1H), 8.69 (s, 1H), 8.15 (d, *J* = 7.8 Hz, 1H), 8.13 – 8.04 (m, 2H), 7.99 (d, *J* = 8.3 Hz, 1H), 7.73 – 7.58 (m, 2H), 7.40 (s, 1H). ^13^C NMR (101 MHz, DMSO) δ 168.6, 149.9, 147.0, 138.0, 135.3, 132.9, 130.2, 130.0, 129.3, 129.3, 128.7, 128.2, 127.8, 124.2, 123.4, 116.0, 94.0. HR-MS (ESI) *m/z* [M+Na]^+^ calcd for C_17_H_10_BF_2_N_3_ONa, 344.0783; found, 344.0781.

**2-cyano-12,12-difluoro-6,12-dihydro-5H-naphtho[2,1-e]pyrazino[1,2-c][1,3,2]oxazaborinin-13-ium-12-uide (4s)**

Yield, 58% (34.4 mg); yellow solid; mp 210-212 ^o^C; ^1^H NMR (400 MHz, DMSO) δ 9.27 (s, 1H), 9.16 (s, 1H), 7.84 (d, *J* = 7.7 Hz, 1H), 7.51 (t, *J* = 7.5, 1.0 Hz, 1H), 7.46 – 7.34 (m, 2H), 3.02 – 2.95 (m, 2H), 2.95 – 2.88 (m, 2H). ^13^C NMR (101 MHz, DMSO) δ 163.6, 147.2, 145.4, 140.9, 137.9, 133.1, 129.7, 128.5, 127.7, 125.1, 123.8, 116.1, 102.7, 26.8, 20.4. HR-MS (ESI) *m/z* [M+Na]^+^ calcd for C_15_H_10_BF_2_N_3_ONa, 320.0783; found, 320.0776.

**2-cyano-13,13-difluoro-5,6,7,13-tetrahydrobenzo[3,4]cyclohepta[1,2-e]pyrazino[1,2-c][1,3,2]oxazaborinin-14-ium-13-uide (4t)**

Yield, 45% (28.0 mg); yellow solid; mp 178-180 ^o^C; ^1^H NMR (400 MHz, DMSO) δ 9.41 (s, 1H), 9.16 (s, 1H), 7.64 (d, *J* = 7.6, 1.1 Hz, 1H), 7.53 (t, *J* = 7.4, 1.4 Hz, 1H), 7.46 (t, *J* = 7.5, 1.1 Hz, 1H), 7.40 (d, *J* = 7.5 Hz, 1H), 2.67 (t, *J* = 6.9 Hz, 2H), 2.47 (t, *J* = 6.8 Hz, 2H), 2.27 – 2.20 (m, 2H). ^13^C NMR (101 MHz, DMSO) δ 169.5, 147.7, 146.1, 141.8, 137.7, 135.3, 132.3, 130.0, 128.9, 127.4, 124.0, 116.0, 106.4, 32.8, 31.4, 22.4. HR-MS (ESI) *m/z* [M+Na]^+^ calcd for C_16_H_12_BF_2_N_3_ONa, 334.0939; found, 334.0935.

**7-cyano-1,1-difluoro-3-(thiophen-2-yl)-1H-pyrazino[1,2-c][1,3,2]oxazaborinin-9-ium-1-uide (4u)**

Yield, 57% (31.5 mg); red solid; mp 245-246 ^o^C; ^1^H NMR (400 MHz, DMSO) δ 9.11 (s, 1H), 9.01 (s, 1H), 8.08 (d, *J* = 4.9 Hz, 1H), 8.04 (d, *J* = 3.8 Hz, 1H), 7.34 (d, *J* = 4.4 Hz, 1H), 7.09 (s, 1H). ^13^C NMR (101 MHz, DMSO) δ 164.3, 149.5, 146.9, 138.0, 137.8, 135.1, 132.3, 130.1, 123.3, 116.1, 92.4. HR-MS (ESI) *m/z* [M+Na]^+^ calcd for C_11_H_6_BF_2_N_3_OSNa, 300.0190; found, 300.0186.

**7-cyano-3-(4-(9,9-dimethyl-9H-fluoren-2-yl)phenyl)-1,1-difluoro-1H-pyrazino[1,2-c][1,3,2]oxazaborinin-9-ium-1-uide (4aa)**

Yield, 12% (11.13 mg);yellow solid; mp 246-248 ^o^C; ^1^H NMR (400 MHz, DMSO) ) δ 9.19 (s, 1H), 9.09 (s, 1H), 8.15 (d, *J* = 8.2 Hz. 2H), 8.02 (d, *J* = 8.5 Hz, 3H), 7.96 (d, *J* = 7.9 Hz, 1H), 7.89 (d, *J* = 5.6 Hz, 1H), 7.80 (d, *J* = 7.8 Hz, 1H), 7.59 (d, *J* = 5.4 Hz, 1H), 7.40 – 7.33 (m, 3H), 1.52 (s, 6H). ^13^C NMR (101 MHz, DMSO) δ 168.6, 154.8, 154.3, 149.7, 147.1, 145.2, 139.6, 138.4, 138.1, 138.0, 131.6, 128.2, 127.8, 127.7, 127.5, 126.6, 124.0, 123.3, 121.9, 121.3, 121.0, 116.0, 93.5, 47.2, 27.3. HR-MS (ESI) *m/z* [M+Na]^+^ calcd for C_28_H_20_BF_2_N_3_ONa, 486.1565; found, 486.1561.

**7-cyano-3-(5-(9,9-dimethyl-9H-fluoren-2-yl)thiophen-2-yl)-1,1-difluoro-1H-pyrazino[1,2-c][1,3,2]oxazaborinin-9-ium-1-uide (4ab)**

Yield, 8% (7.51 mg);red solid; mp 206-208 ^o^C; ^1^H NMR (400 MHz, DMSO) δ 9.09 (s, 1H), 8.97 (s, 1H), 8.11 (d, *J* = 4.1 Hz, 1H), 8.05 (s, 1H), 7.93 (d, *J* = 8.0 Hz, 1H), 7.88 (dd, *J* = 9.5, 3.9 Hz, 2H), 7.84 – 7.79 (m, 1H), 7.62 – 7.54 (m, 1H), 7.41 – 7.33 (m, 2H), 7.13 (s, 1H), 1.51 (s, 6H). ^13^C NMR (101 MHz, DMSO) δ 164.0, 154.9, 154.3, 152.5, 149.6, 146.7, 140.4, 138.2, 138.0, 136.0, 133.9, 132.0, 128.5, 127.7, 126.5, 125.7, 123.4, 122.9, 121.5, 121.1, 121.0, 116.2, 92.4, 47.2, 27.1. HR-MS (ESI) *m/z* [M+Na]^+^ calcd for C_26_H_18_BF_2_N_3_OSNa, 492.1129; found, 492.1123.

**7-cyano-1,1-difluoro-3-(4-(octyloxy)phenyl)-1H-pyrazino[1,2-c][1,3,2]oxazaborinin-9-ium-1-uide (4ac)**

Yield, 48% (38.31 mg);yellow solid; mp 165-167 ^o^C; ^1^H NMR (400 MHz, CDCl_3_) δ 8.66 (s, 1H), 8.33 (s, 1H), 7.98 (d, *J* = 8.9 Hz, 1H), 7.26 (s, 1H), 6.98 (d, *J* = 8.9 Hz, 1H), 6.50 (s, 1H), 4.05 (t, *J* = 6.5 Hz, 1H), 1.87 – 1.76 (m, 1H), 1.58 (s, 1H), 1.51 – 1.42 (m, 1H), 1.38 – 1.25 (m, 3H), 0.89 (t, *J* = 6.6 Hz, 1H). ^13^C NMR (101 MHz, CDCl_3_) δ 172.7, 164.2, 148.1, 146.7, 136.2, 130.2, 124.4, 122.0, 115.0, 114.7, 90.4, 68.6, 31.8, 29.3, 29.2, 29.1, 26.0, 22.7, 14.1. HR-MS (ESI) *m/z* [M+Na]^+^ calcd for C_21_H_24_BF_2_N_3_O_2_Na, 422.1827; found, 422.1833.

**1-(4-(9,9-dimethyl-9H-fluoren-2-yl)phenyl)ethanone (4a_1_)**

white solid; ^1^H NMR (400 MHz, CDCl_3_) δ 8.07 (d, *J* = 8.4 Hz, 2H), 7.81 (d, *J* = 7.9 Hz, 1H), 7.79 – 7.74 (m, 3H), 7.72 (s, 1H), 7.62 (dd, *J* = 7.9, 1.5 Hz, 1H), 7.52 – 7.46 (m, 1H), 7.42 – 7.33 (m, 2H), 2.66 (s, 3H), 1.57 (s, 6H). ^13^C NMR (101 MHz, CDCl_3_) δ 197.8, 154.5, 154.0, 146.2, 139.5, 138.9, 138.6, 135.7, 129.0, 127.7, 127.3, 127.2, 126.5, 122.8, 121.5, 120.5, 120.3, 47.1, 27.3, 26.7.

**1-(5-(9,9-dimethyl-9H-fluoren-2-yl)thiophen-2-yl)ethanone (4a_2_)**

white solid; ^1^H NMR (400 MHz, CDCl_3_) δ 7.76 – 7.70 (m, 2H), 7.69 (s, 1H), 7.66 (d, *J* = 4.0 Hz,1H), 7.63 (dd, *J* = 7.9, 1.5 Hz, 1H), 7.48 – 7.41 (m, 1H), 7.39 – 7.30 (m, 3H), 2.57 (s, 3H), 1.52 (s, 6H). ^13^C NMR (101 MHz, CDCl_3_) δ 190.6, 154.5, 154.0, 153.5, 142.7, 140.4, 138.3, 133.6, 132.25, 127.9, 127.2, 125.5, 123.7, 122.7, 120.6, 120.6, 120.4, 47.0, 27.1, 26.6.

**1-(4-(octyloxy)phenyl)ethanone (4a_3_)**

Oil; ^1^H NMR (400 MHz, CDCl_3_) δ 7.90 (d, *J* = 8.9 Hz, 2H), 6.89 (d, *J* = 8.9 Hz, 2H), 3.99 (t, *J* = 6.6 Hz, 2H), 2.53 (s, 3H), 1.84 – 1.69 (m, 2H), 1.50 – 1.39 (m, 2H), 1.36 – 1.23 (m, 8H), 0.87 (t, *J* = 6.8 Hz, 3H). ^13^C NMR (101 MHz, CDCl_3_) δ 196.8, 163.1, 130.6, 130.1, 114.1, 68.3, 31.8, 29.3, 29.2, 29.1, 26.3, 26.0, 22.7, 14.1.

**(Z)-3-amino-1-phenyl-3-(pyrazin-2-yl)prop-2-en-1-one (5)**

yellow solid; ^1^H NMR (400 MHz, CDCl_3_) δ 10.18 (s, 1H), 9.22 (s, 1H), 8.67 (dd, *J* = 11.4, 1.9 Hz, 2H), 7.98 (d, *J* = 6.6 Hz, 2H), 7.54 – 7.42 (m, 3H), 7.08 (s, 1H), 6.60 (s, 1H). ^13^C NMR (101 MHz, CDCl_3_) δ 191.0, 154.5, 147.0, 145.9, 143.6, 142.2, 140.0, 131.5, 128.5, 127.3, 90.0.

**4. X-ray Structure Determinations**

Diffraction-quality crystals were obtained from the following solvents: Chloroform/hexane (**4a**, **4aa** and **4ab**), Chloroform (**5**). Diffraction data were collected on an Oxford Diffraction Supernova dual diffractometer equipped with an Oxford Cryostream 700 low-temperature apparatus. Cu K\a radiation source (*λ* = 1.54184 Å) were used for the data collection. Single crystals were coated with Paratone-N oil and mounted on a Nylon loop for diffraction. The data reduction and cell refinement were processed using CrysAlisPro software.^1^ Structures were solved by direct methods using the SHELXTL program packages.^2^ All non-hydrogen atoms were refined anisotropically and hydrogen atoms were added geometrically. Crystal data and refinement details were given in Tables S1. Other refinement details and explanations were included in individual CIF files.


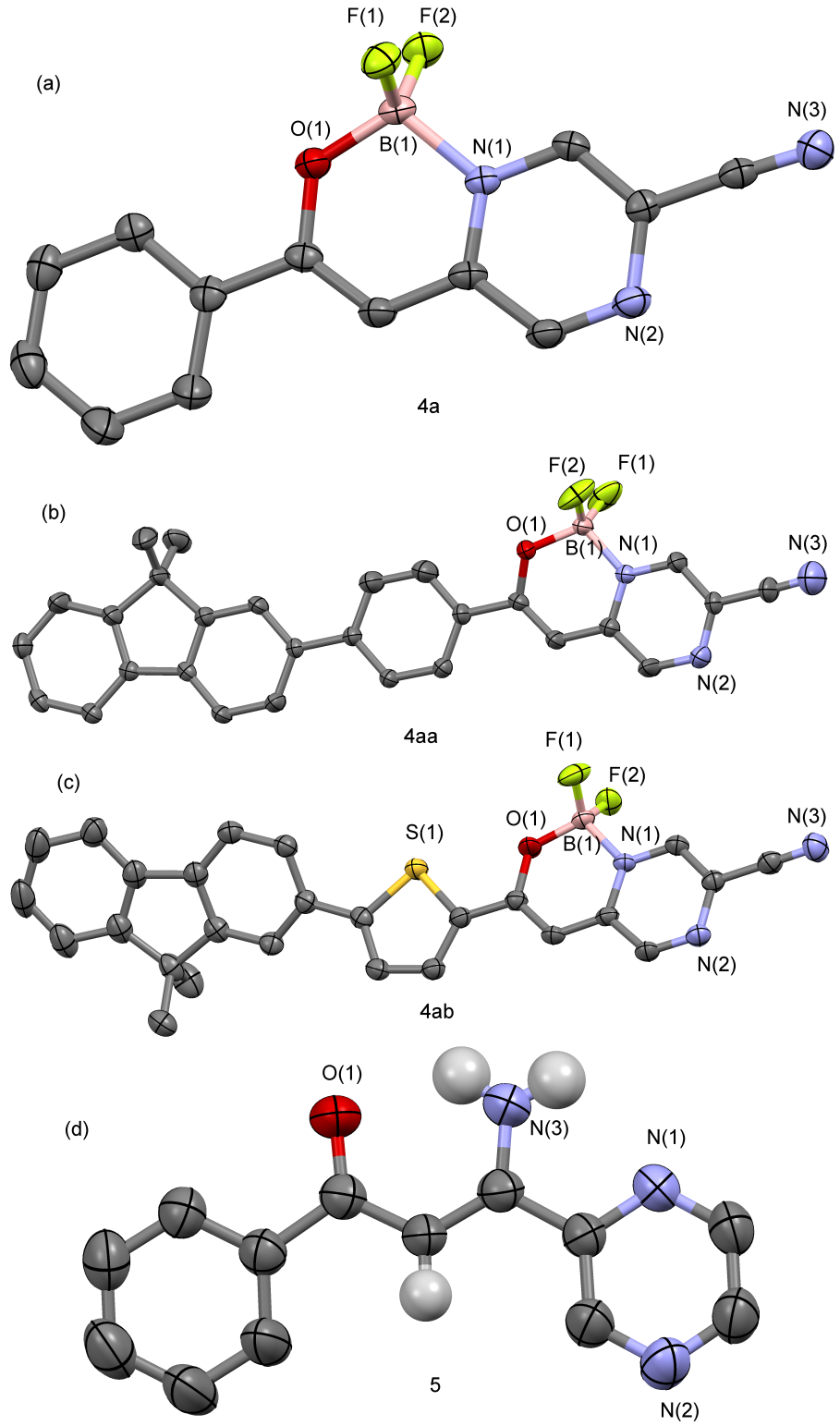


**Fig. S1** Crystal structures of compounds **4a** (a), **4aa** (b), **4ab** (c) and **5** (d) as 50% probability ellipsoids.

**5. Crystallographic data of compounds**

**Table S1.** **Crystallographic data for compounds 4a, 4aa, 4ab and 5 *^a^***

|  | **4a***^b^* | **4aa***^c^* | **4ab***^d^* | **5***^e^* |
| --- | --- | --- | --- | --- |
| formula | C_13_H_8_BF_2_N_3_O | C_28_H_20_BF_2_N_3_O | C_27_H_19_BCl_3_F_2_N_3_OS | C_13_H_11_N_3_O |
| *M* | 271.03 | 463.28 | 588.67 | 225.25 |
| crystal system | Orthorhombic | Monoclinic | Monoclinic | Orthorhombic |
| space group | P2_1_2_1_2_1_ | P2_1_/m | P2_1_/c | P2_1_2_1_2_1_ |
| *a*, Å | 5.3416(2) | 8.6992(7) | 17.5431(4) | 6.9059(7) |
| *b*, Å | 13.1660(4) | 6.9631(6) | 12.9697(2) | 11.6510(6) |
| *c*, Å | 16.9223(7) | 18.3825(14) | 12.1624(2) | 14.2914(12) |
| *α*, deg | 90 | 90 | 90 | 90 |
| *β*, deg | 90 | 98.989(7) | 99.290(2) | 90 |
| γ, deg | 90 | 90 | 90 | 90 |
| *V*, Å^3^ | 1190.10(8) | 1099.81(15) | 2730.99(9) | 1149.90(16) |
| Z | 4 | 2 | 4 | 4 |
| *μ*, mm^-1^ | 1.020 | 0.798 | 4.100 | 0.695 |
| independent data | 2149 | 2254 | 5174 | 2139 |
| refined parameters | 181 | 208 | 345 | 155 |
| *R_1_^f^, wR_2_^g^*(*I*>2*σ*(*I*)) | 0.0359, 0.0896 | 0.0739, 0.1855 | 0.0579, 0.1558 | 0.0496, 0.1392 |
| *R_1_,wR_2_* (all data) | 0.0414, 0.0930 | 0.1239, 0.2089 | 0.0698, 0.1646 | 0.0671, 0.1592 |

*^a^*Cu Kα radiation (*λ* = 1.54184 Å). *^b^*T = 150(2) K. *^c^*T = 130(2) K. *^d^*T = 157(2) K. *^e^*T = 280(2) K. *^f^R_1_* = Σ||*F_o_*| – |*F_c_*||/Σ|*F_o_*|. *^g^wR_2_* = {Σ[w(*Fo*^2^ – *F_c_*^2^)^2^/(*F_o_*^2^)^2^]}^1/2^.

**6. ^1^H NMR and ^13^C NMR spectra of compounds**

**7-cyano-1,1-difluoro-3-phenyl-1H-pyrazino[1,2-c][1,3,2]oxazaborinin-9-ium-1-uide (4a)**

**
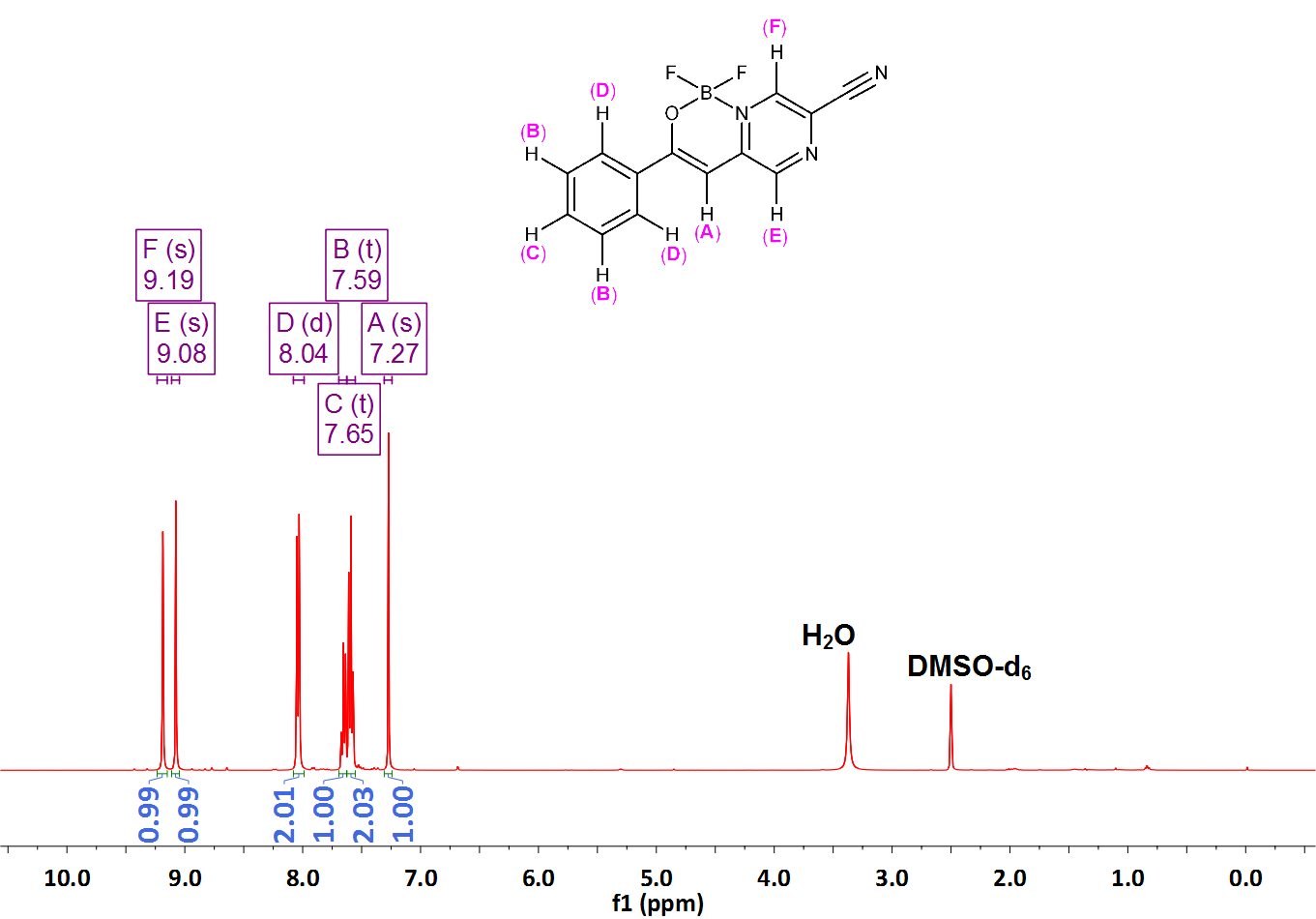
**

**
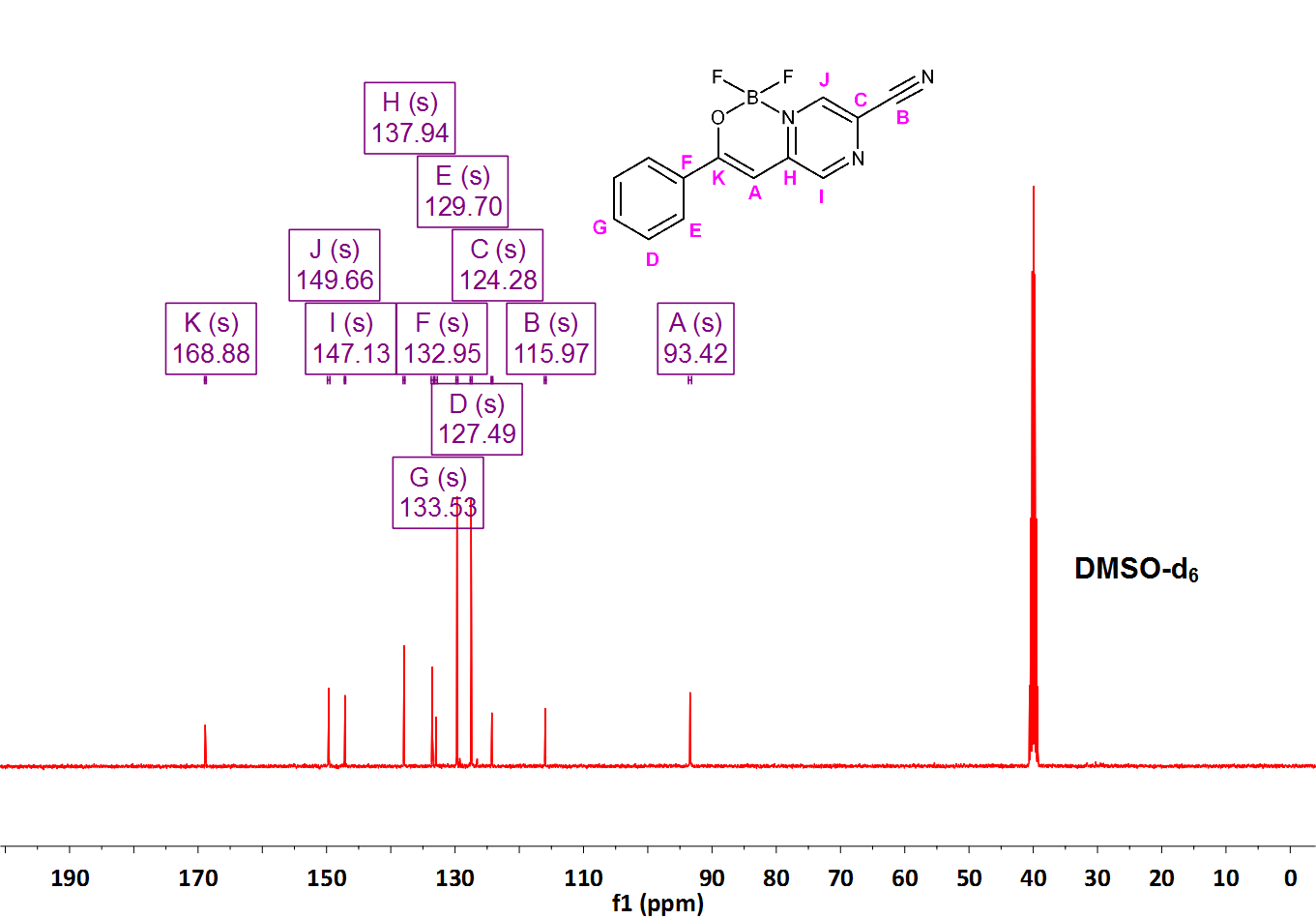
**

**7-cyano-1,1-difluoro-3-(4-methoxyphenyl)-1H-pyrazino[1,2-c][1,3,2]oxazaborinin-9-ium-1-uide (4b)**

**
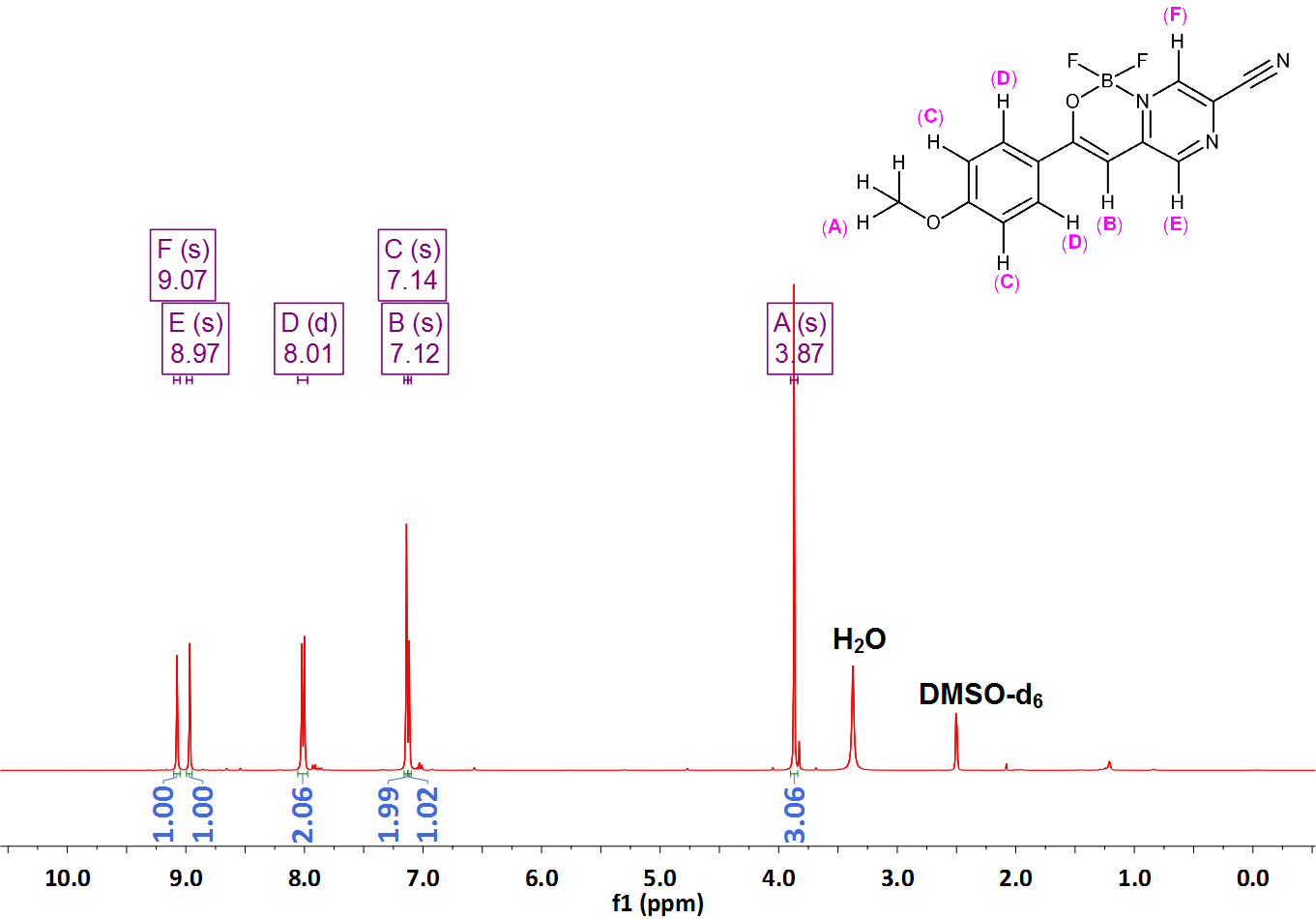
**

**
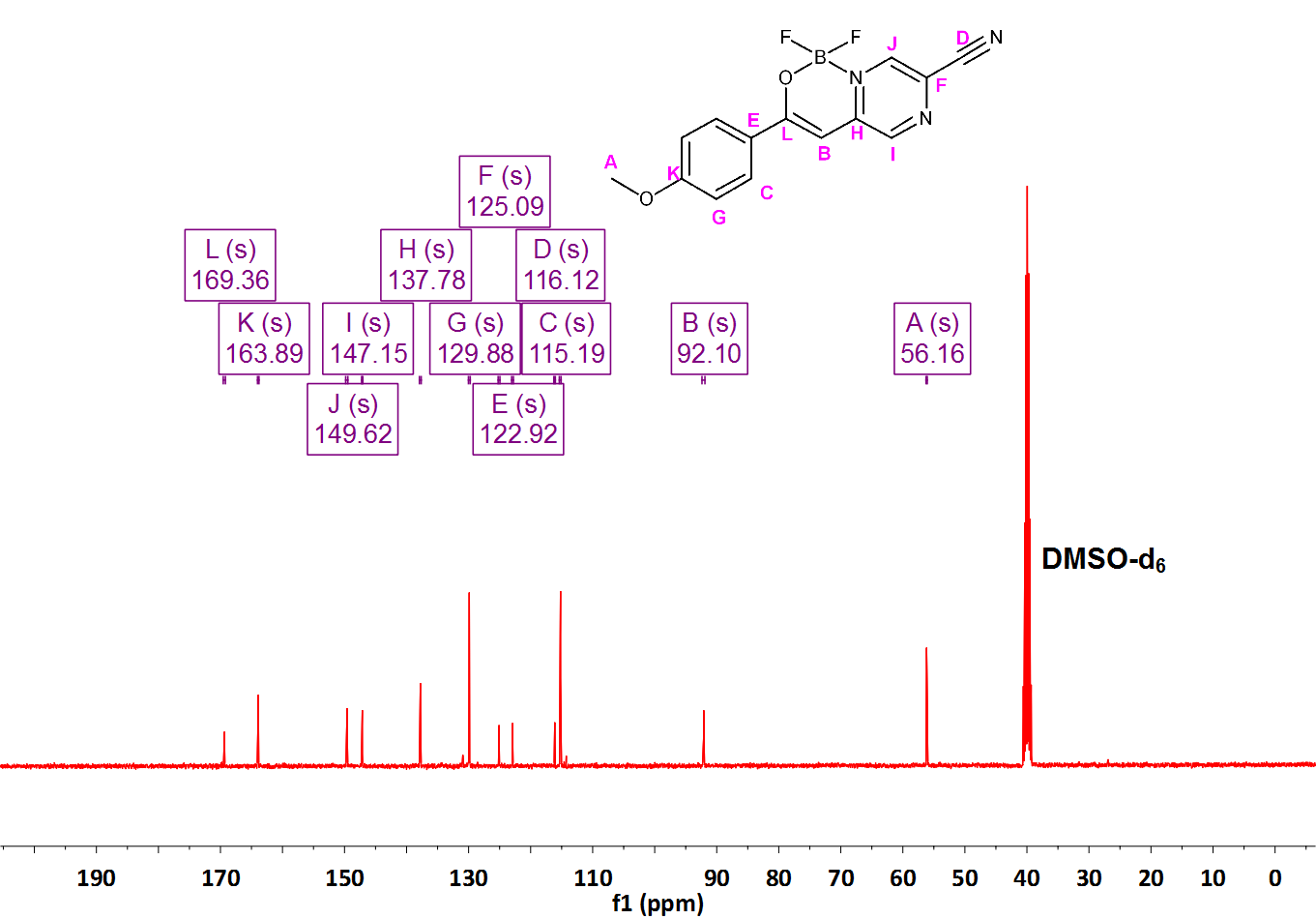
**

**7-cyano-1,1-difluoro-3-(4-(methylthio)phenyl)-1H-pyrazino[1,2-c][1,3,2]oxazaborinin-9-ium-1-uide (4c)**

**
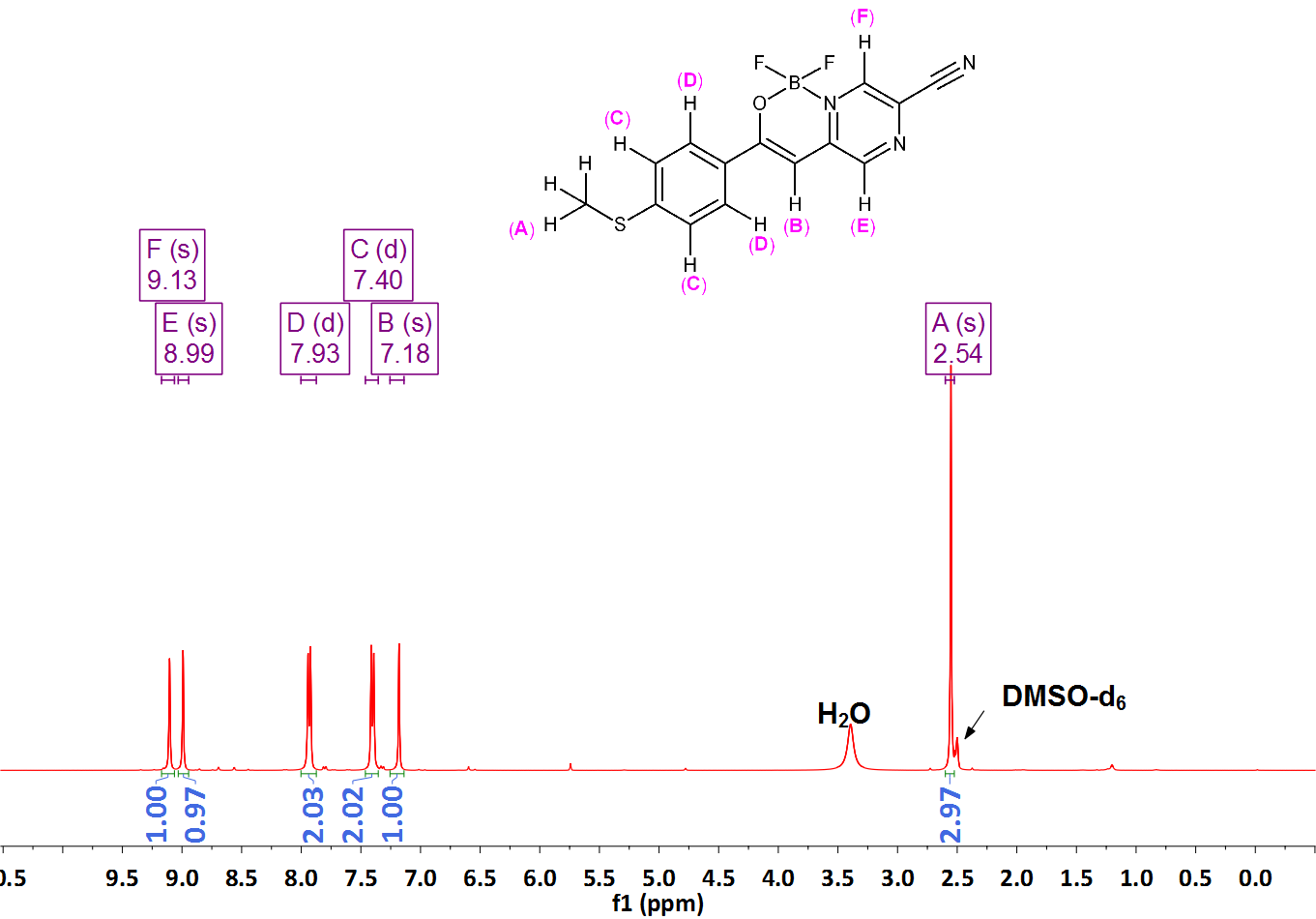
**

**
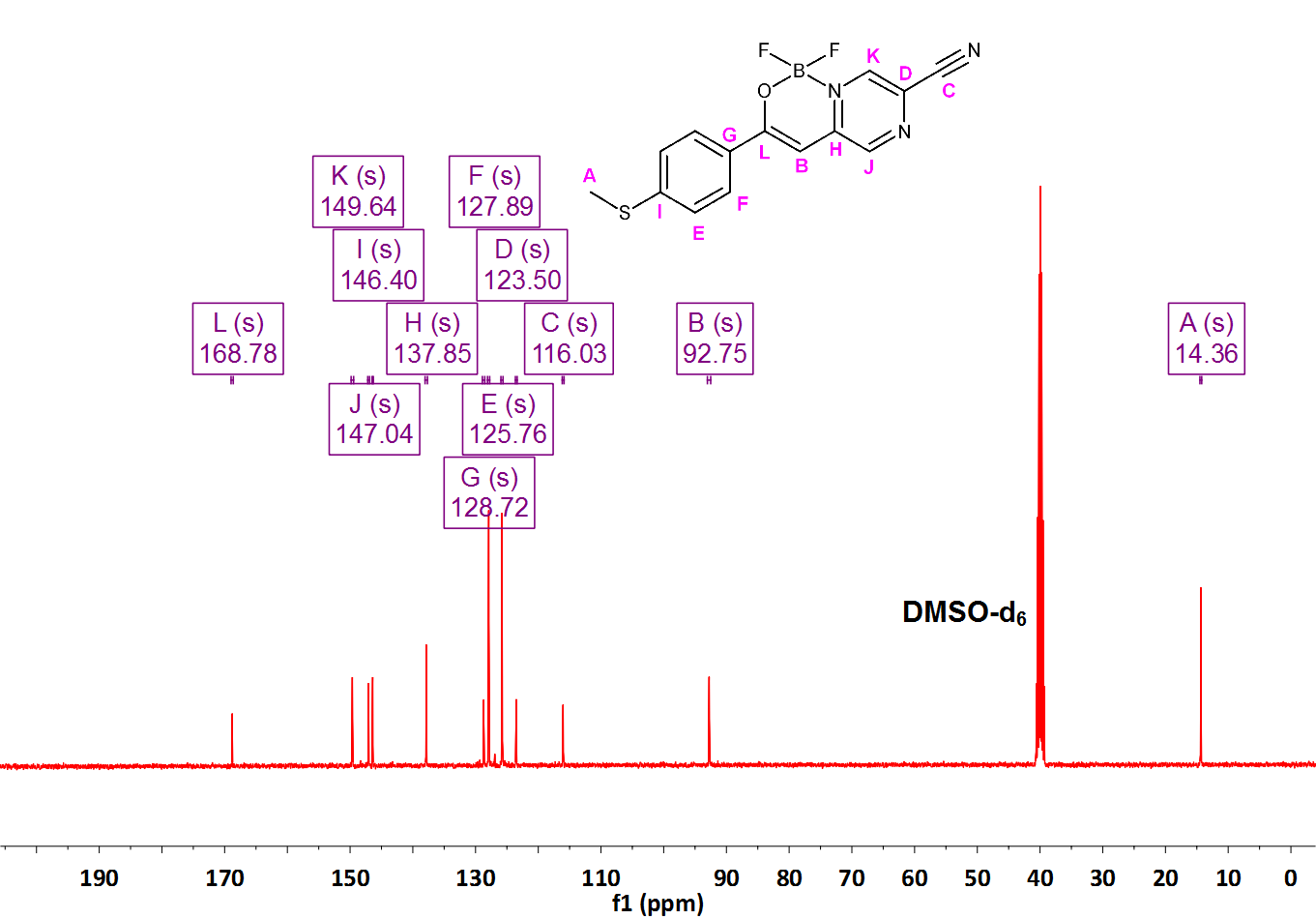
**

**3-(4-(tert-butyl)phenyl)-7-cyano-1,1-difluoro-1H-pyrazino[1,2-c][1,3,2]oxazaborinin-9-ium-1-uide (4d)**

**
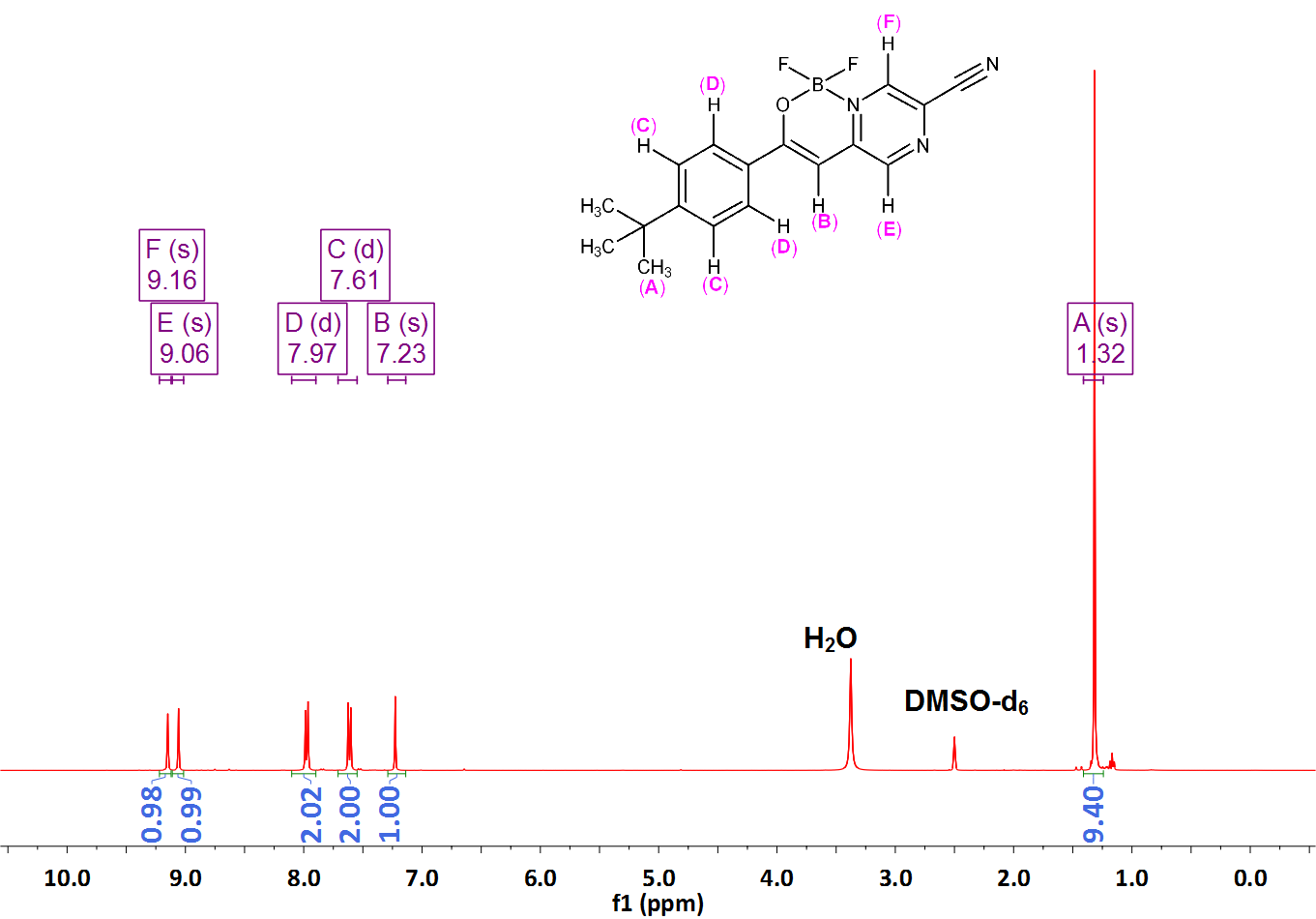
**

**
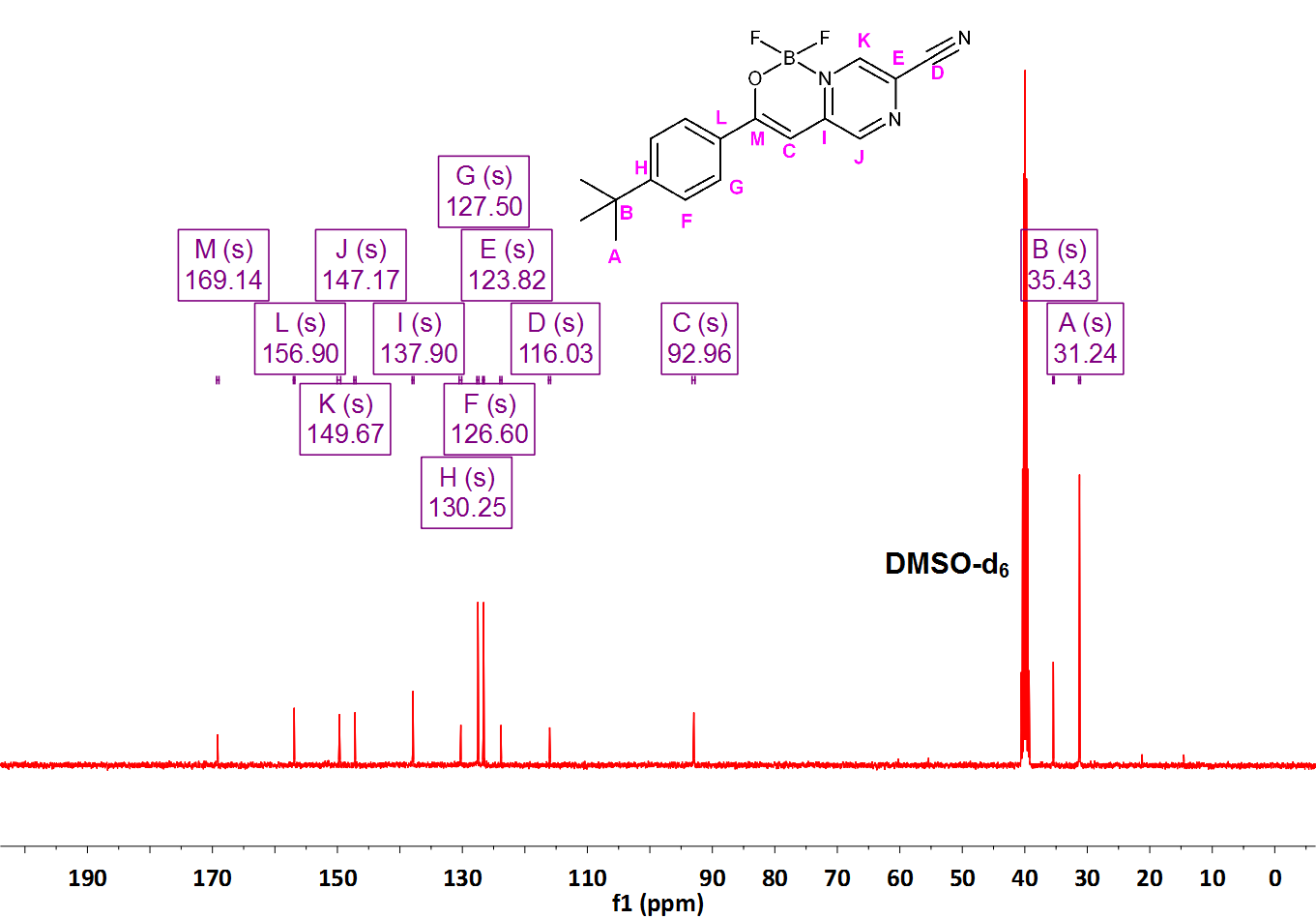
**

**7-cyano-3-(4-ethylphenyl)-1,1-difluoro-1H-pyrazino[1,2-c][1,3,2]oxazaborinin-9-ium-1-uide (4e)**

**
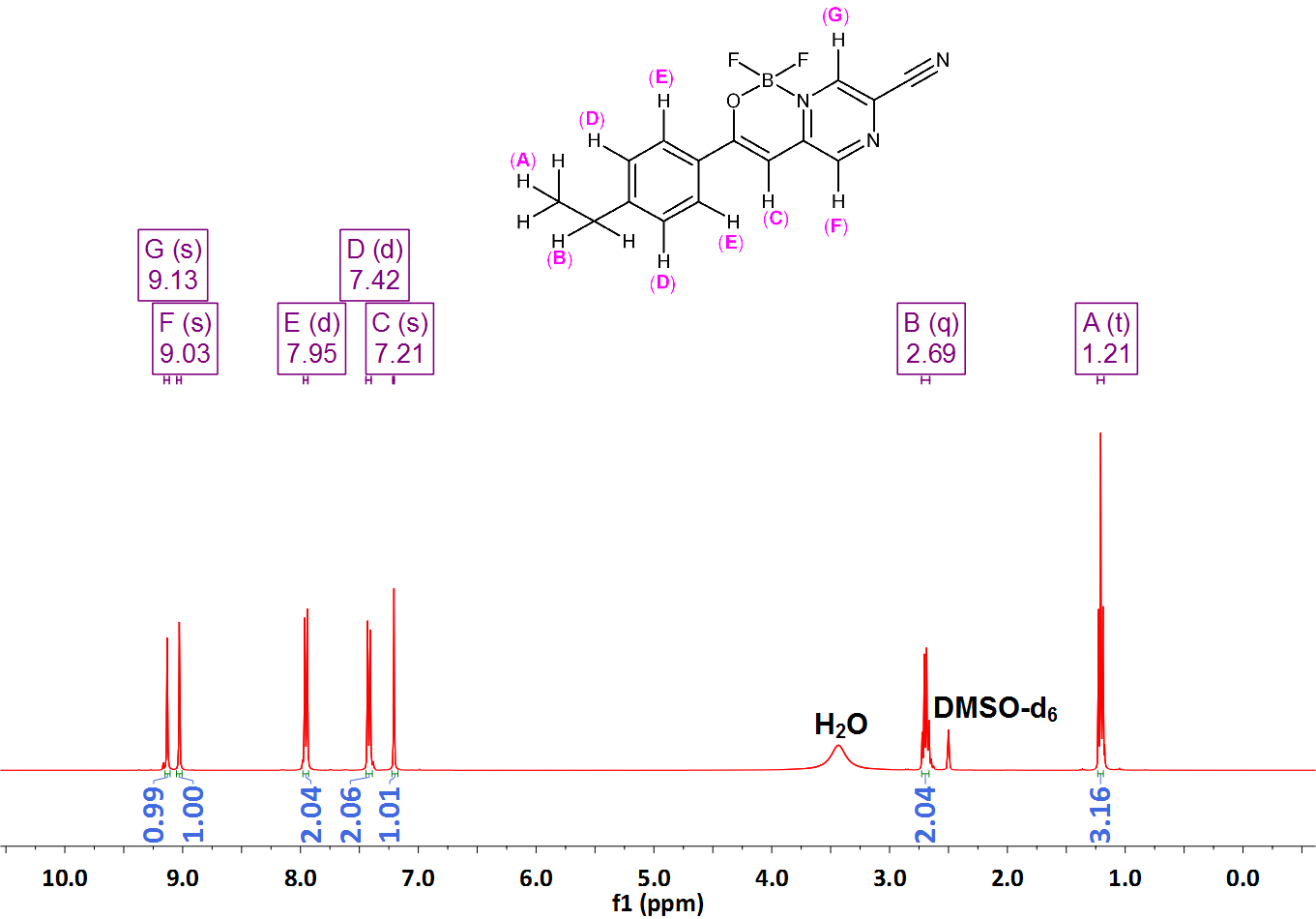
**

**
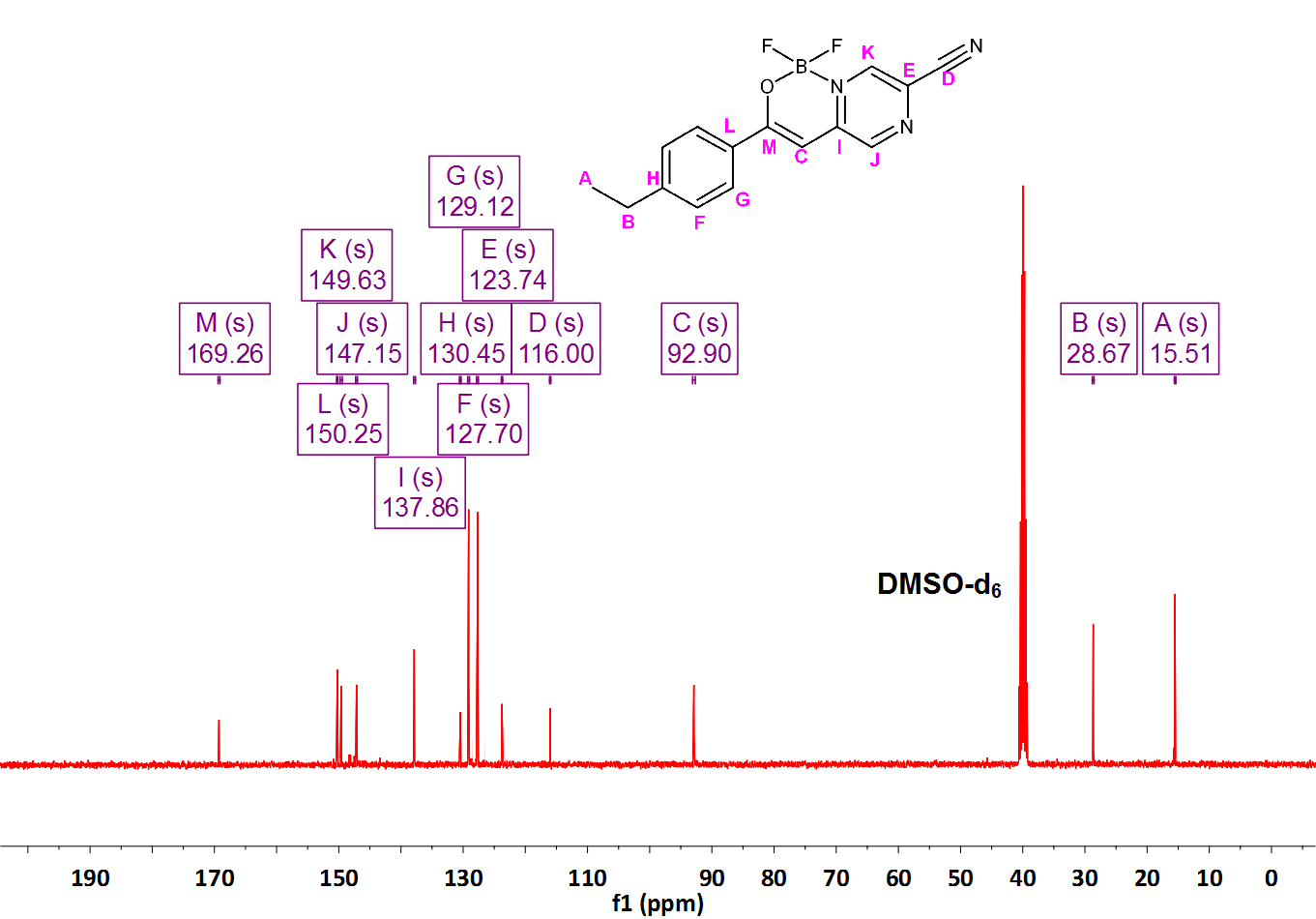
**

**7-cyano-1,1-difluoro-3-(p-tolyl)-1H-pyrazino[1,2-c][1,3,2]oxazaborinin-9-ium-1-uide (4f)**

**
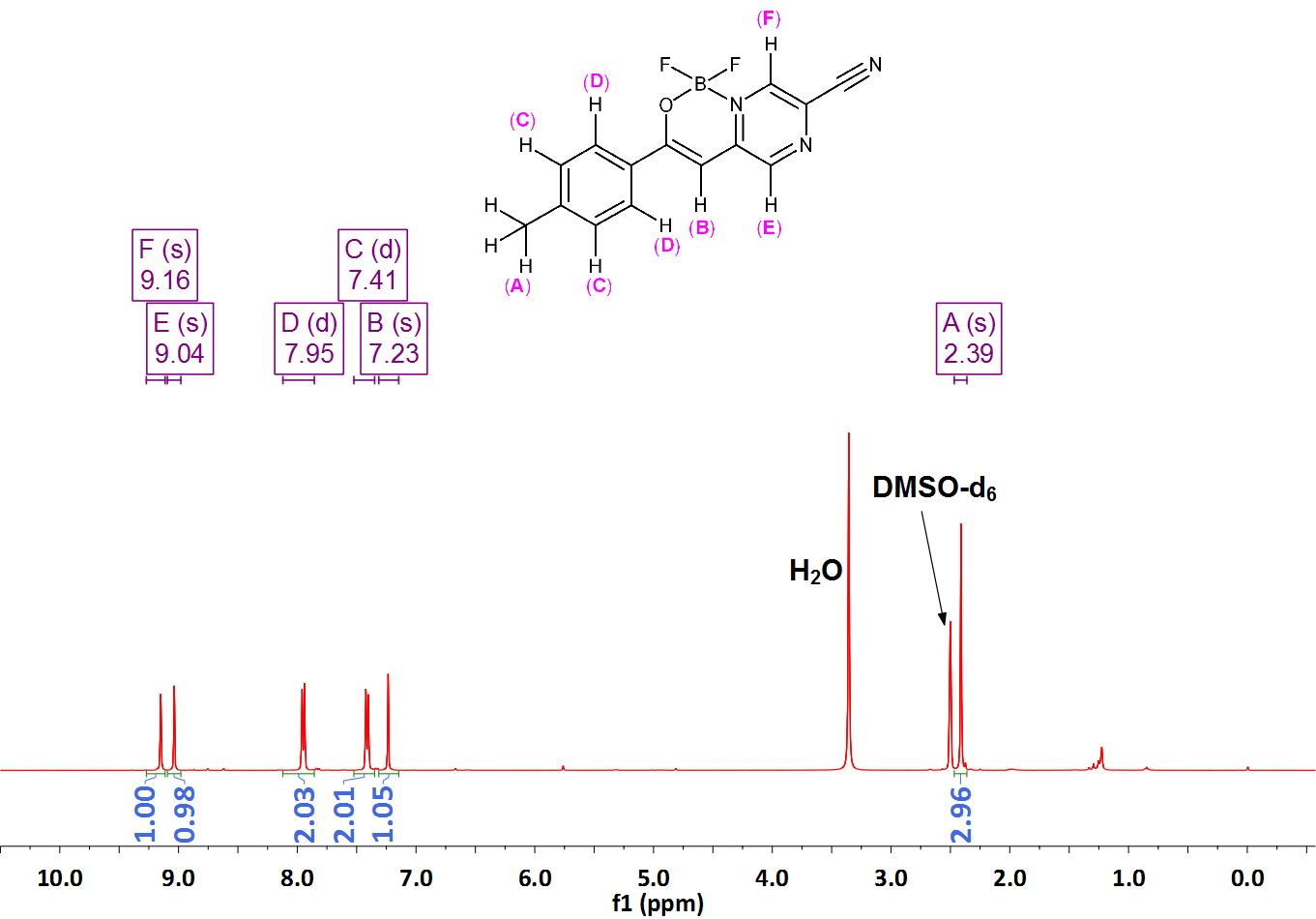
**

**
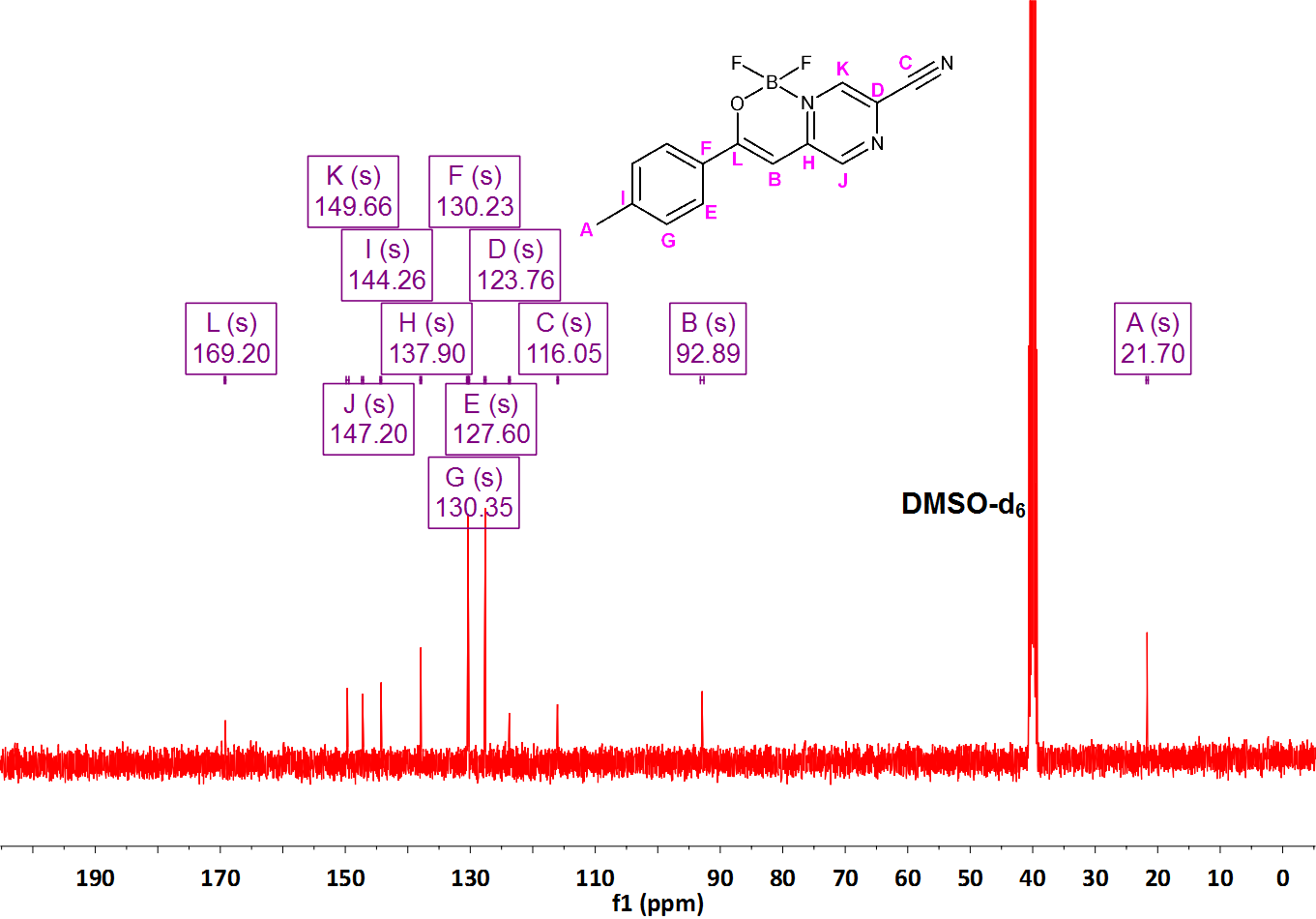
**

**3-([1,1'-biphenyl]-4-yl)-7-cyano-1,1-difluoro-1H-pyrazino[1,2-c][1,3,2]oxazaborinin-9-ium-1-uide (4g)**

**
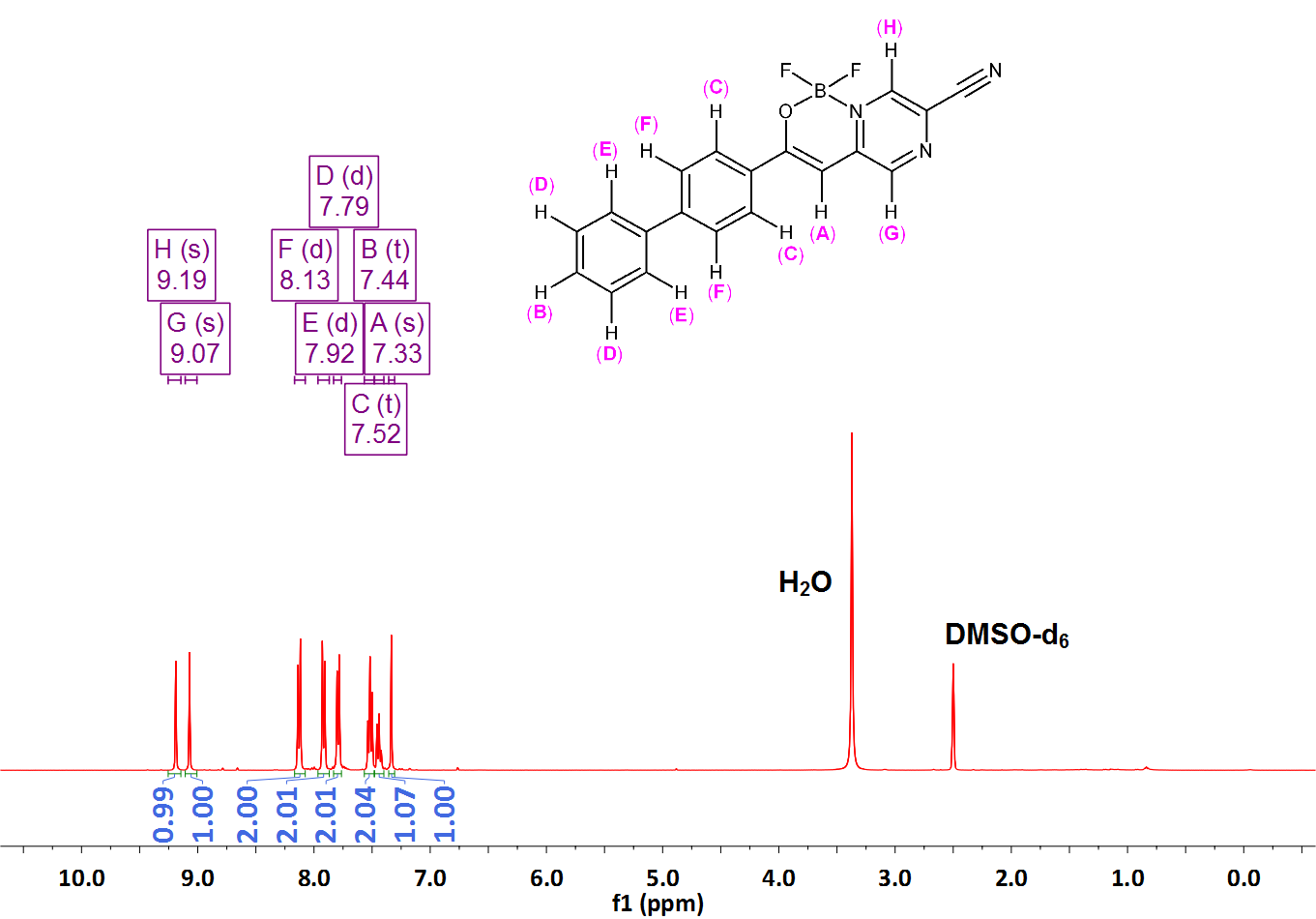
**

**
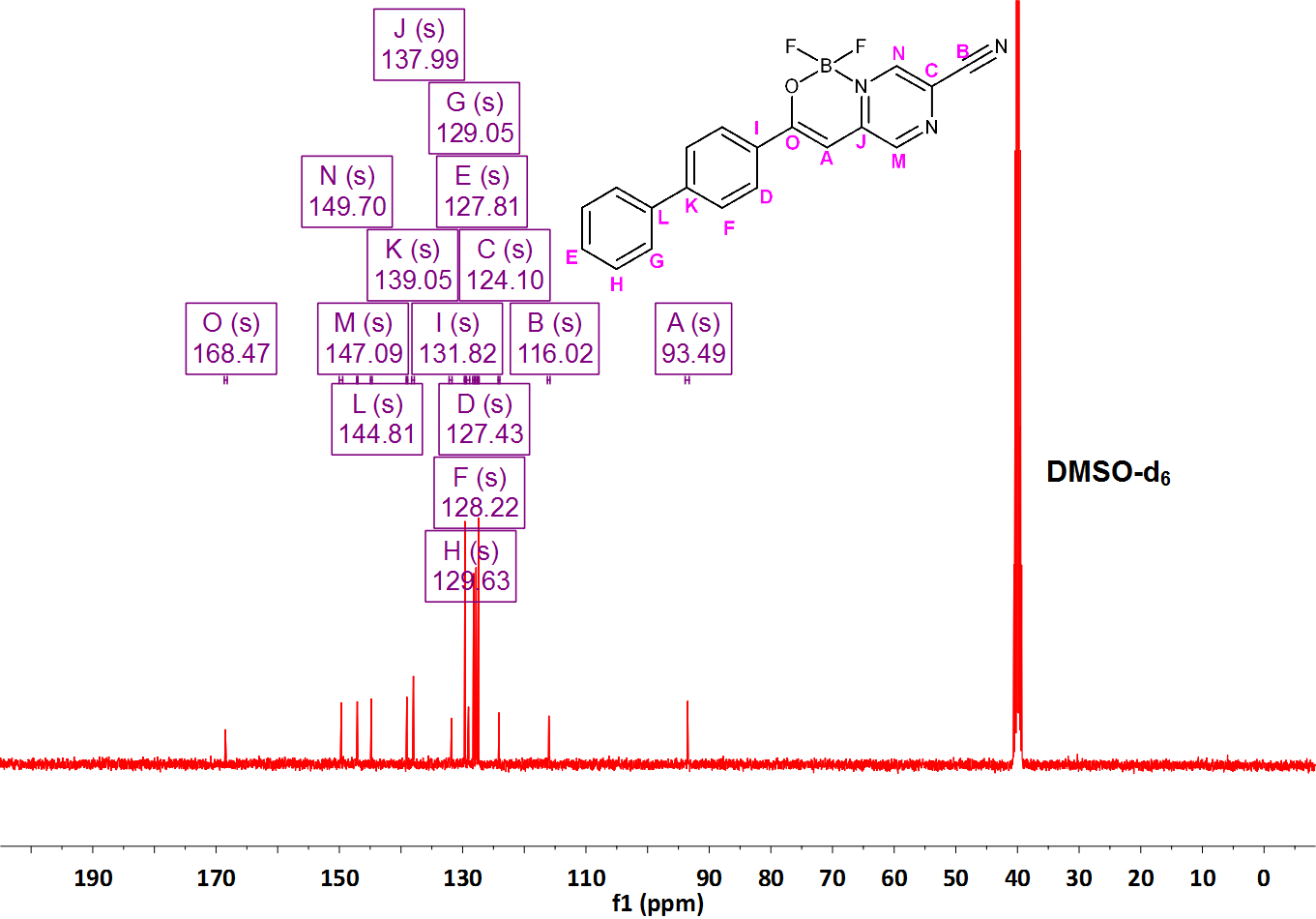
**

**7-cyano-1,1-difluoro-3-(4-iodophenyl)-1H-pyrazino[1,2-c][1,3,2]oxazaborinin-9-ium-1-uide (4h)**

**
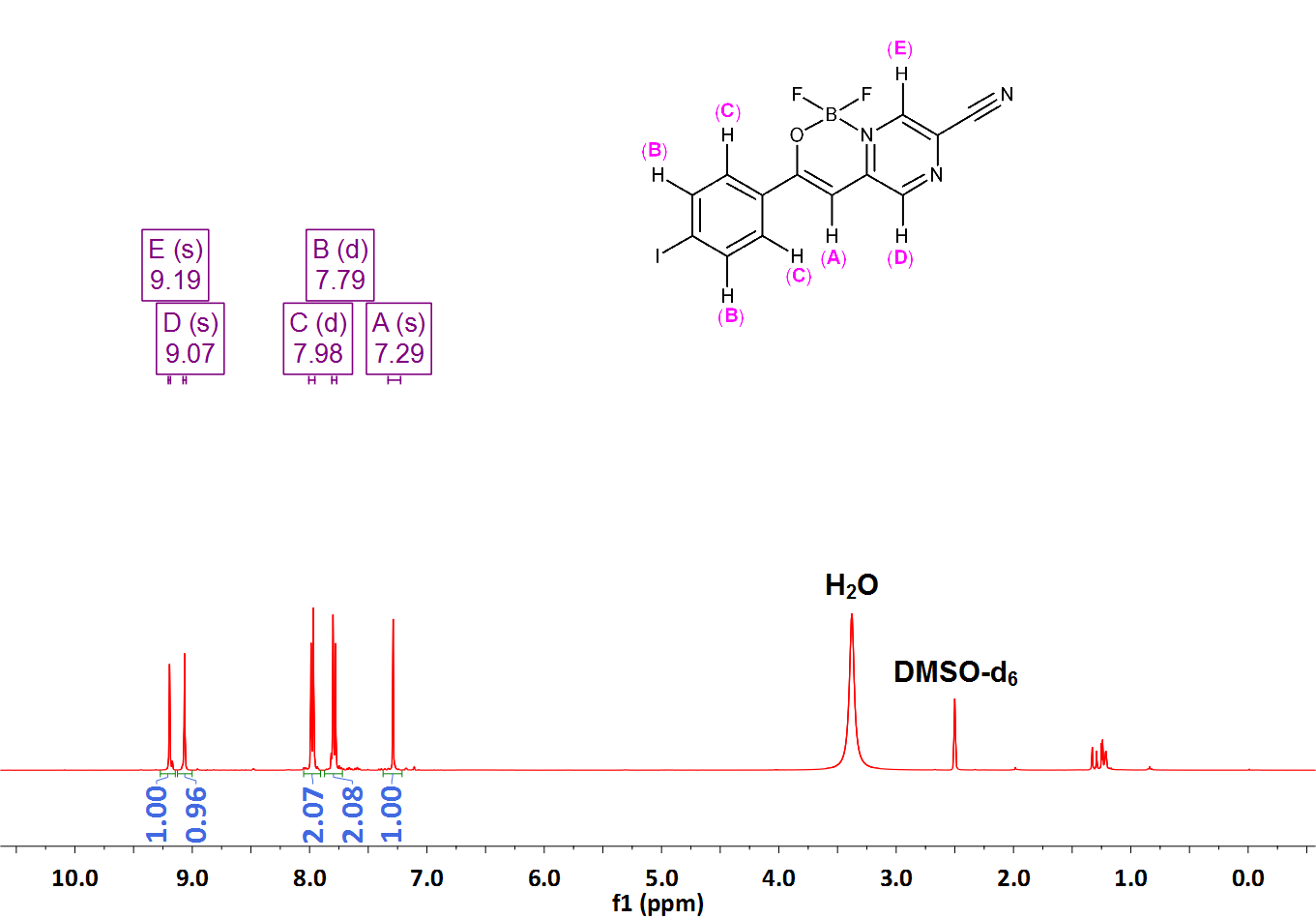
**

**
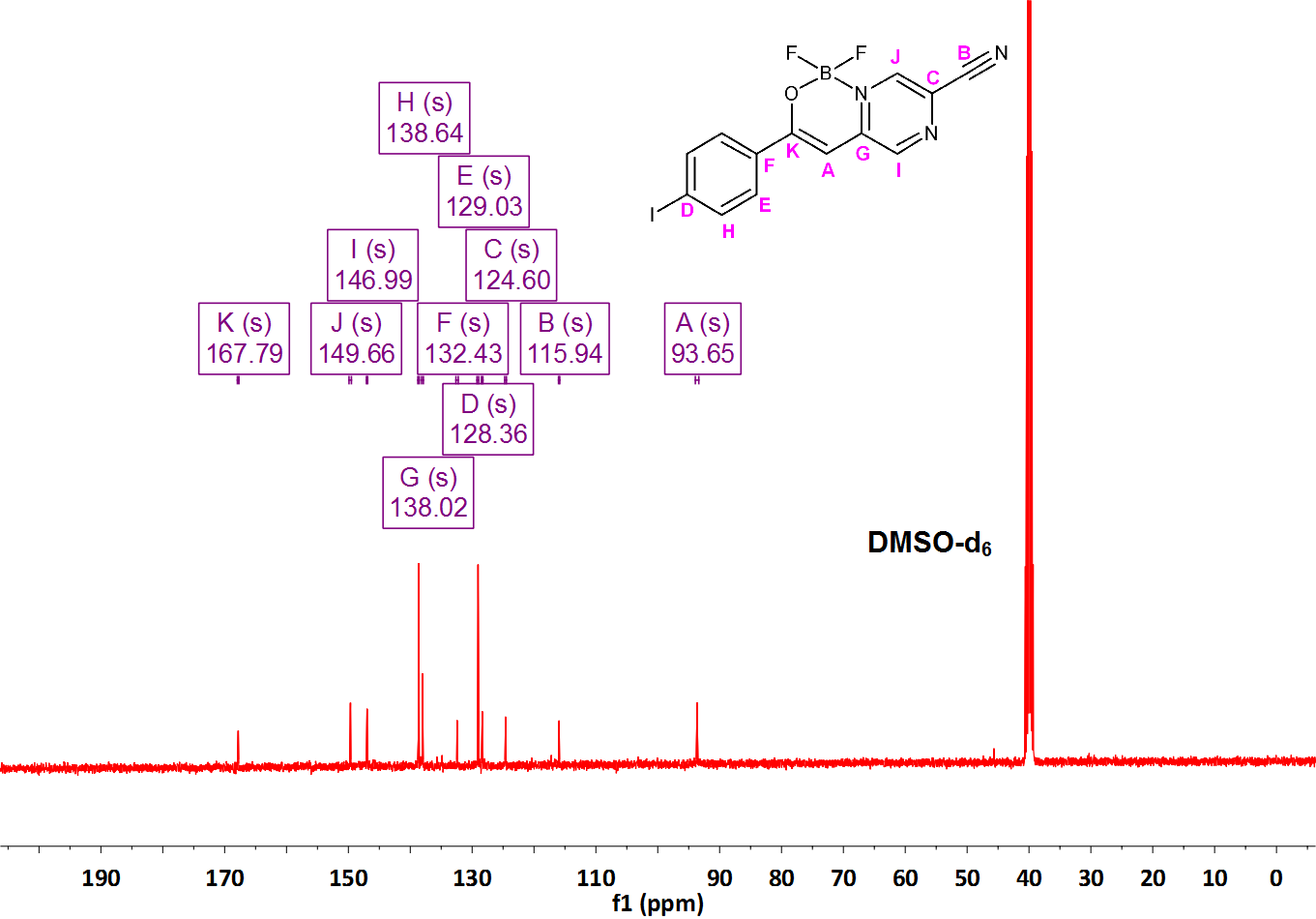
**

**3-(4-bromophenyl)-7-cyano-1,1-difluoro-1H-pyrazino[1,2-c][1,3,2]oxazaborinin-9-ium-1-uide (4i)**

**
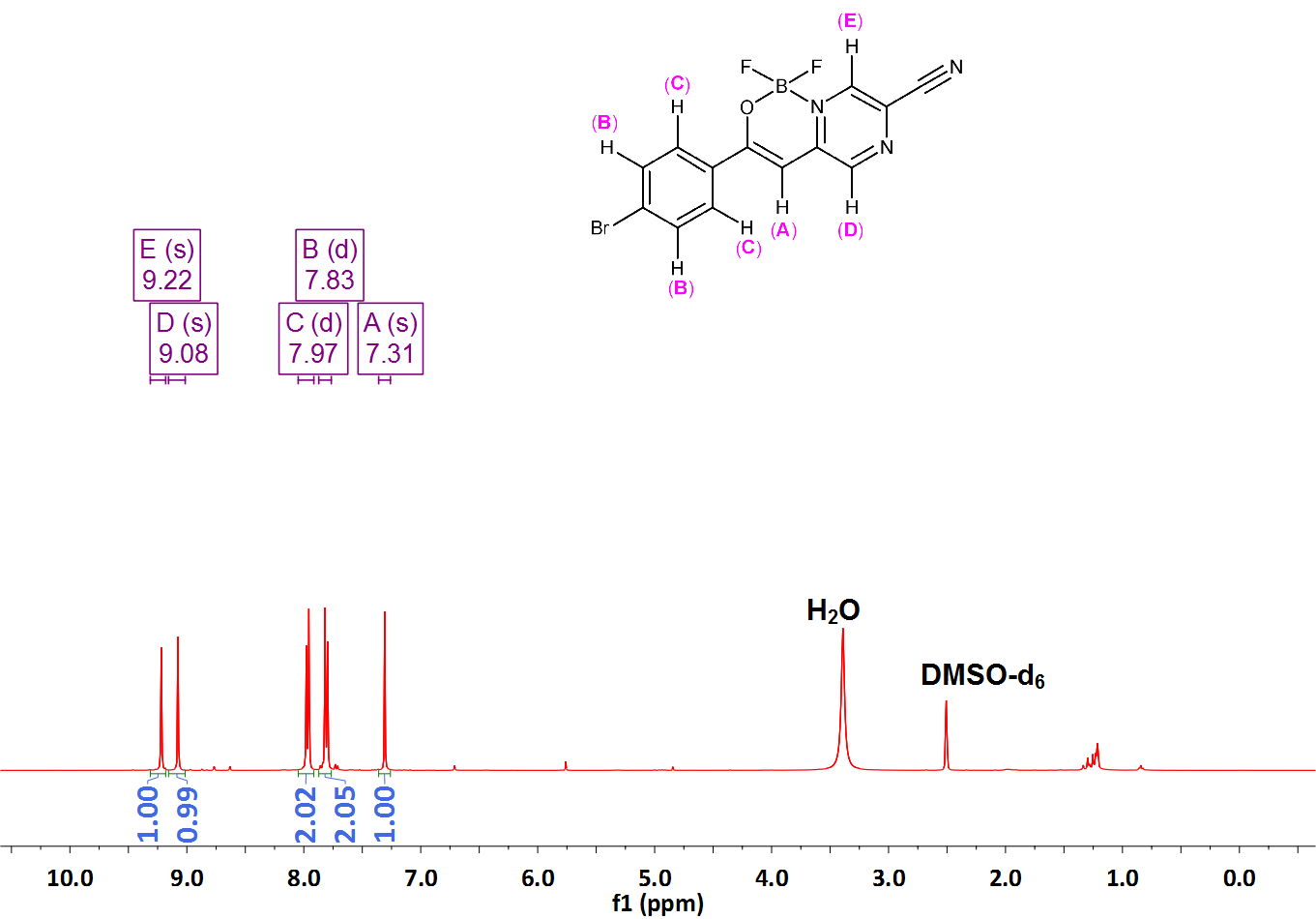
**

**
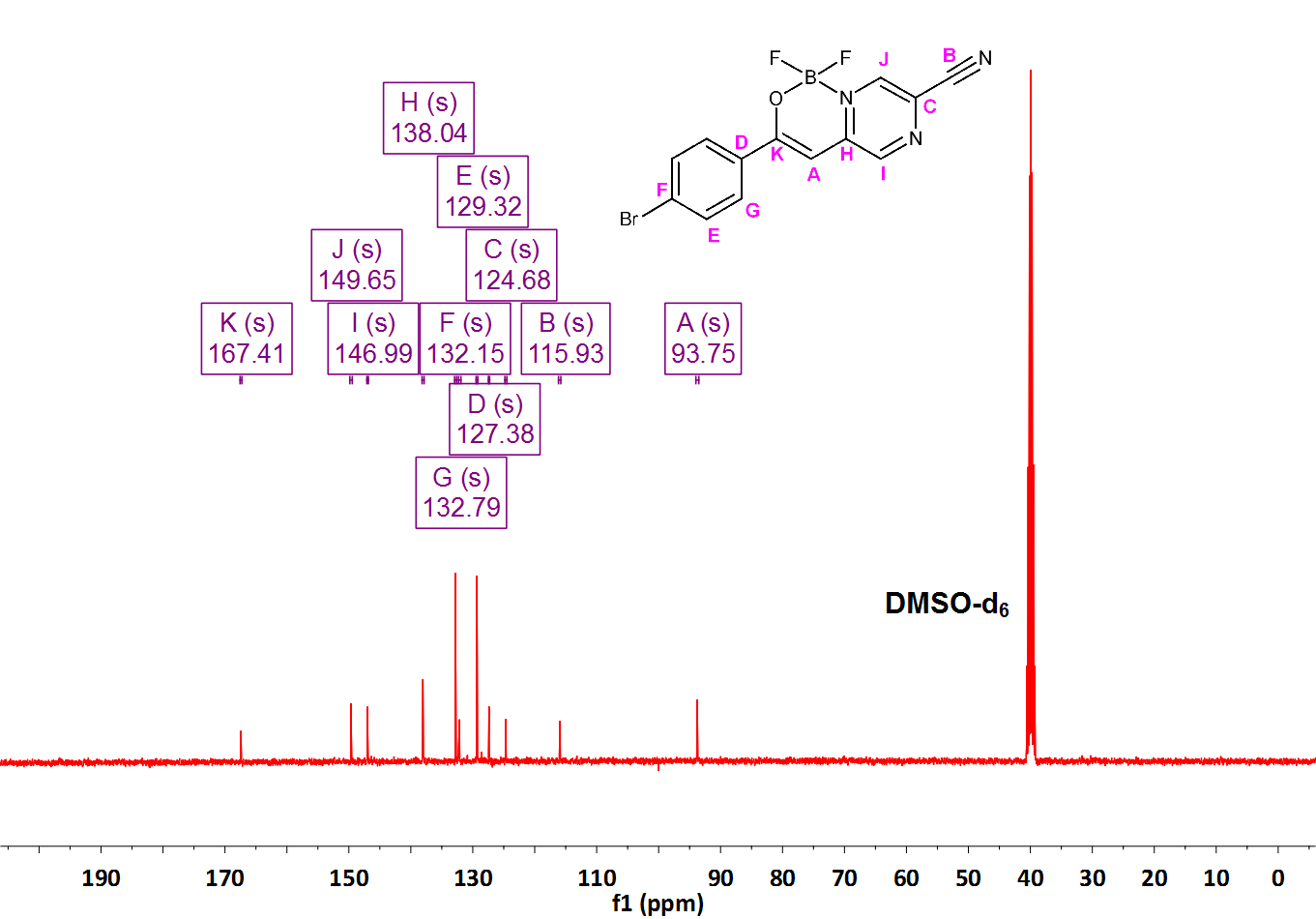
**

**3-(4-chlorophenyl)-7-cyano-1,1-difluoro-1H-pyrazino[1,2-c][1,3,2]oxazaborinin-9-ium-1-uide (4j)**

**
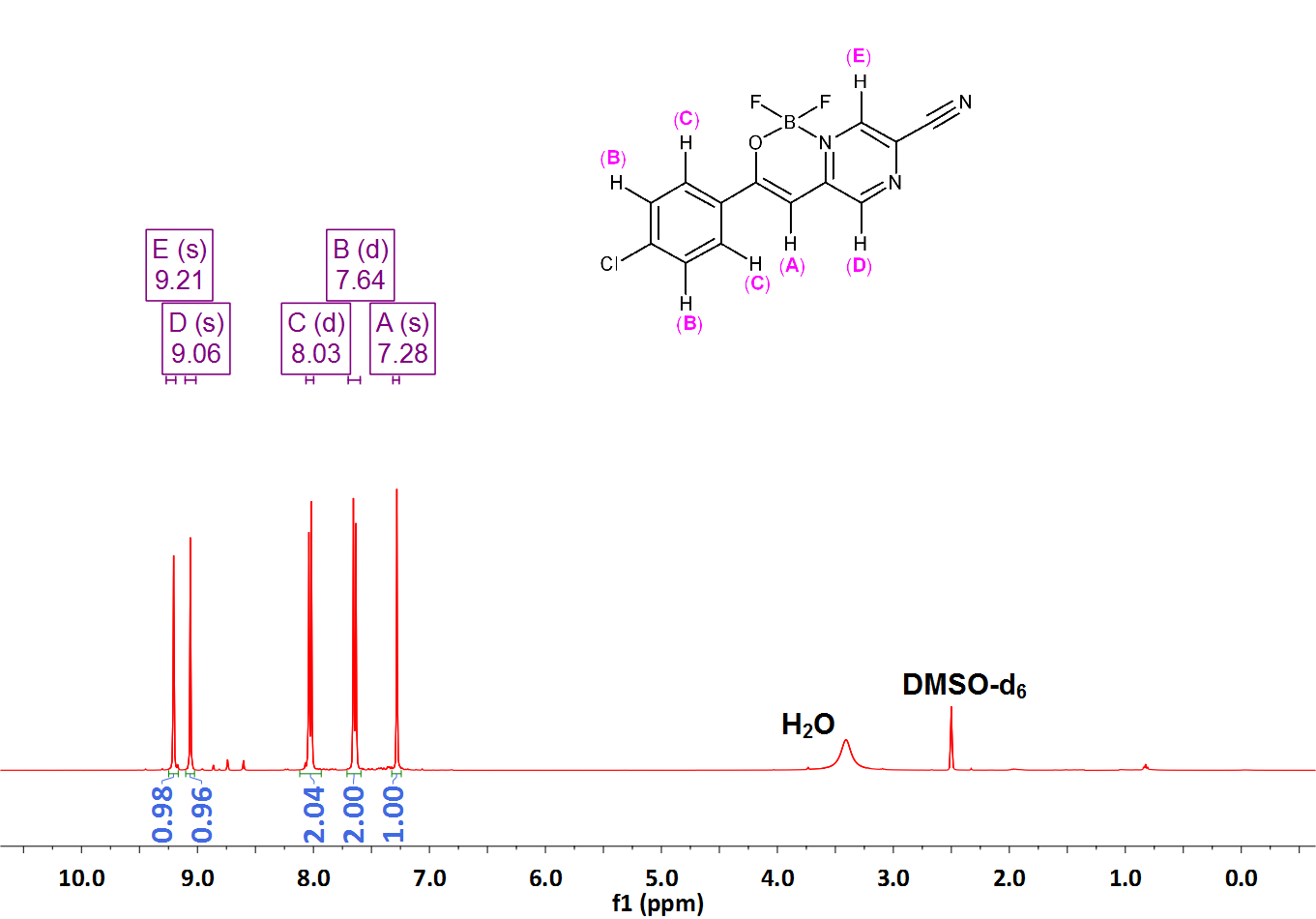
**

**
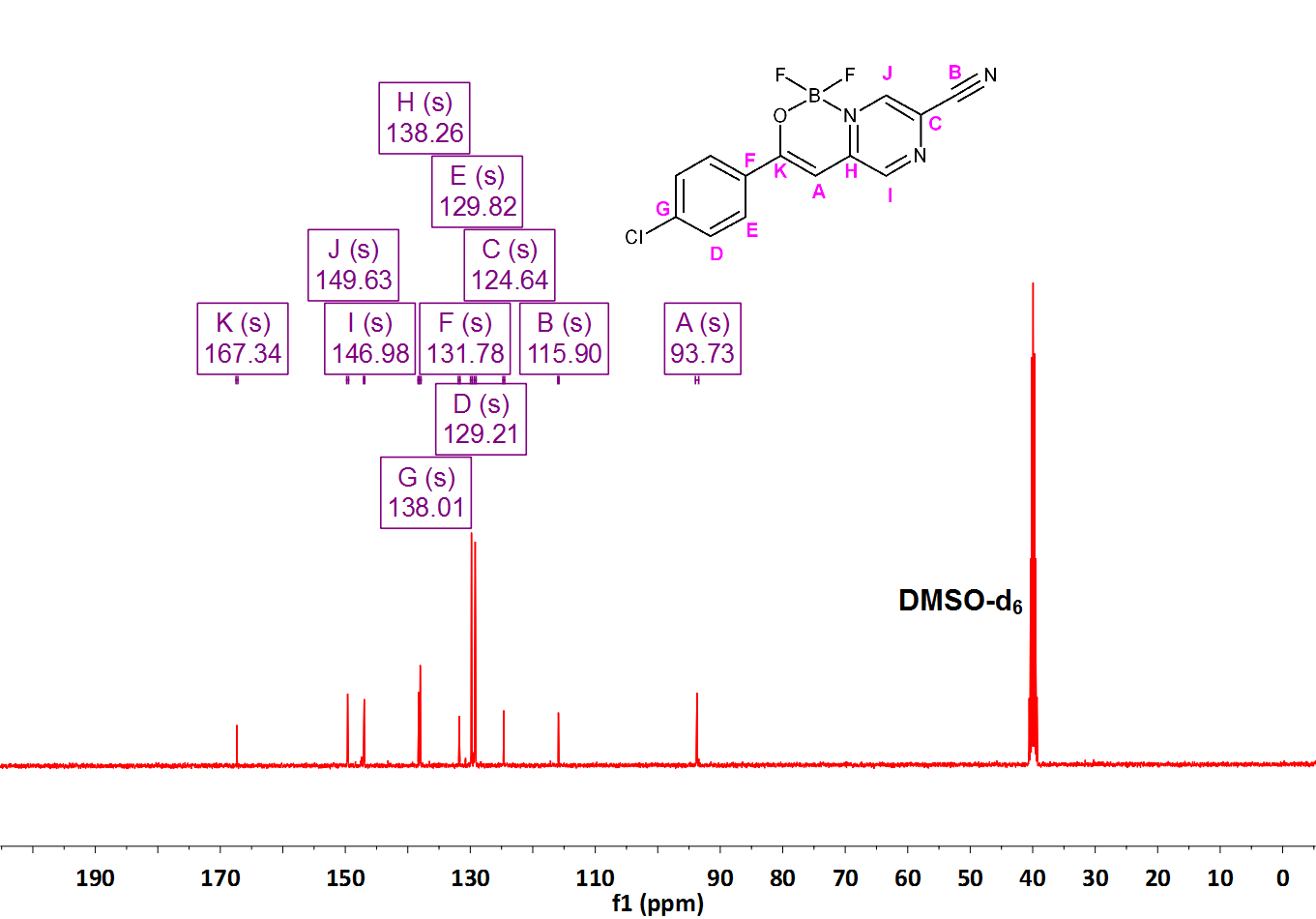
**

**7-cyano-1,1-difluoro-3-(4-fluorophenyl)-1H-pyrazino[1,2-c][1,3,2]oxazaborinin-9-ium-1-uide (4k)**

**
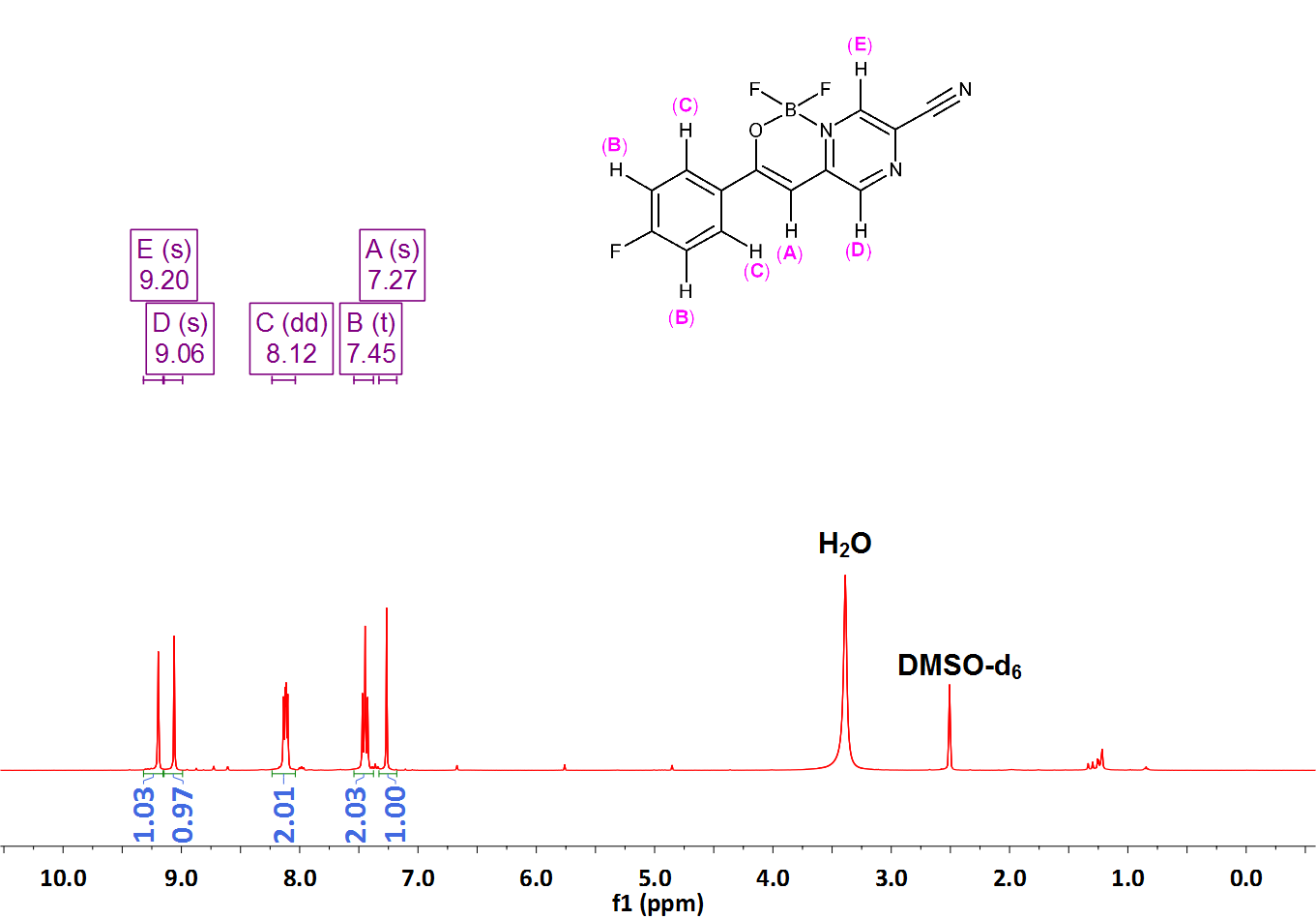
**

**
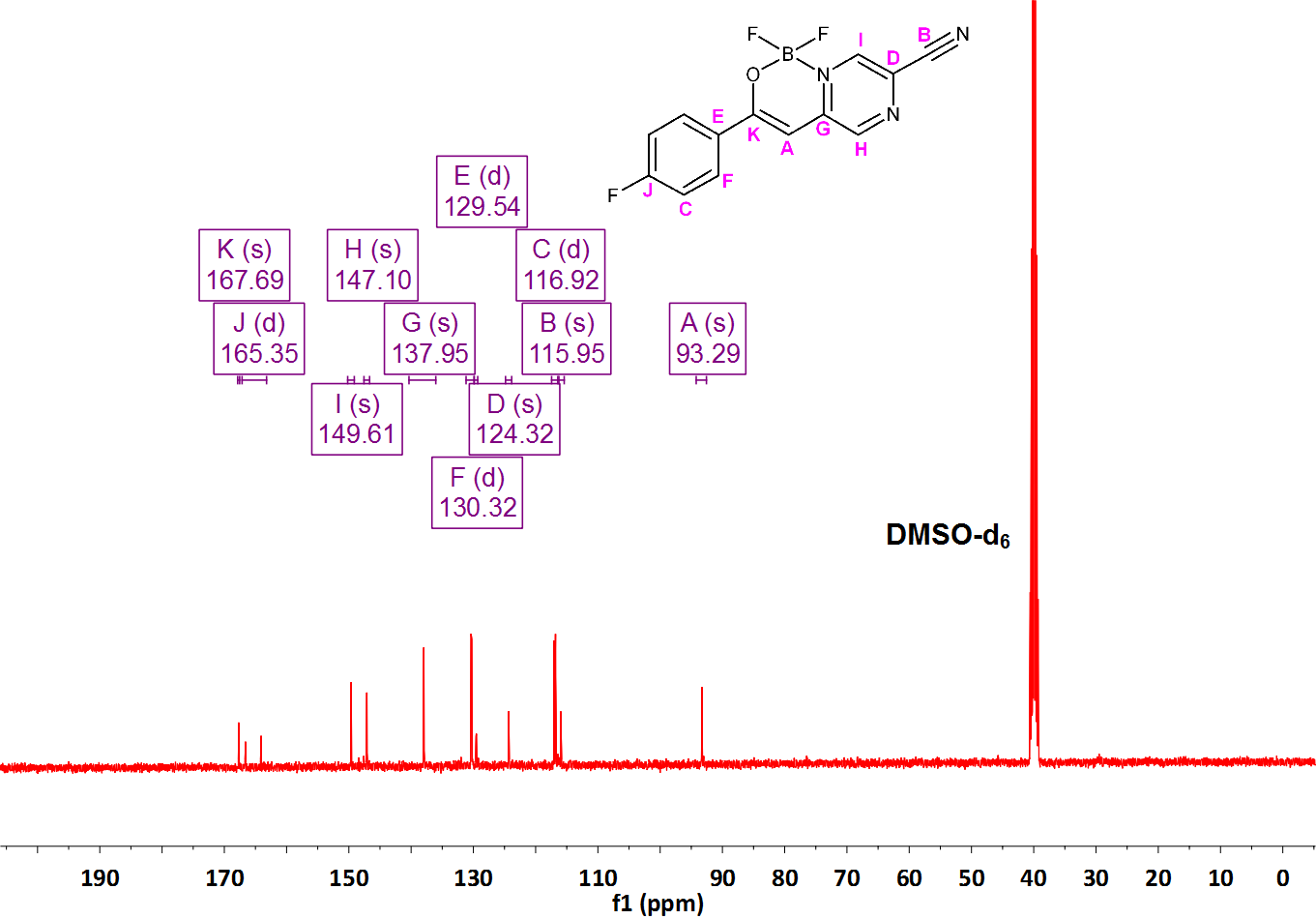
**

**7-cyano-1,1-difluoro-3-(3-methoxyphenyl)-1H-pyrazino[1,2-c][1,3,2]oxazaborinin-9-ium-1-uide (4l)**

**
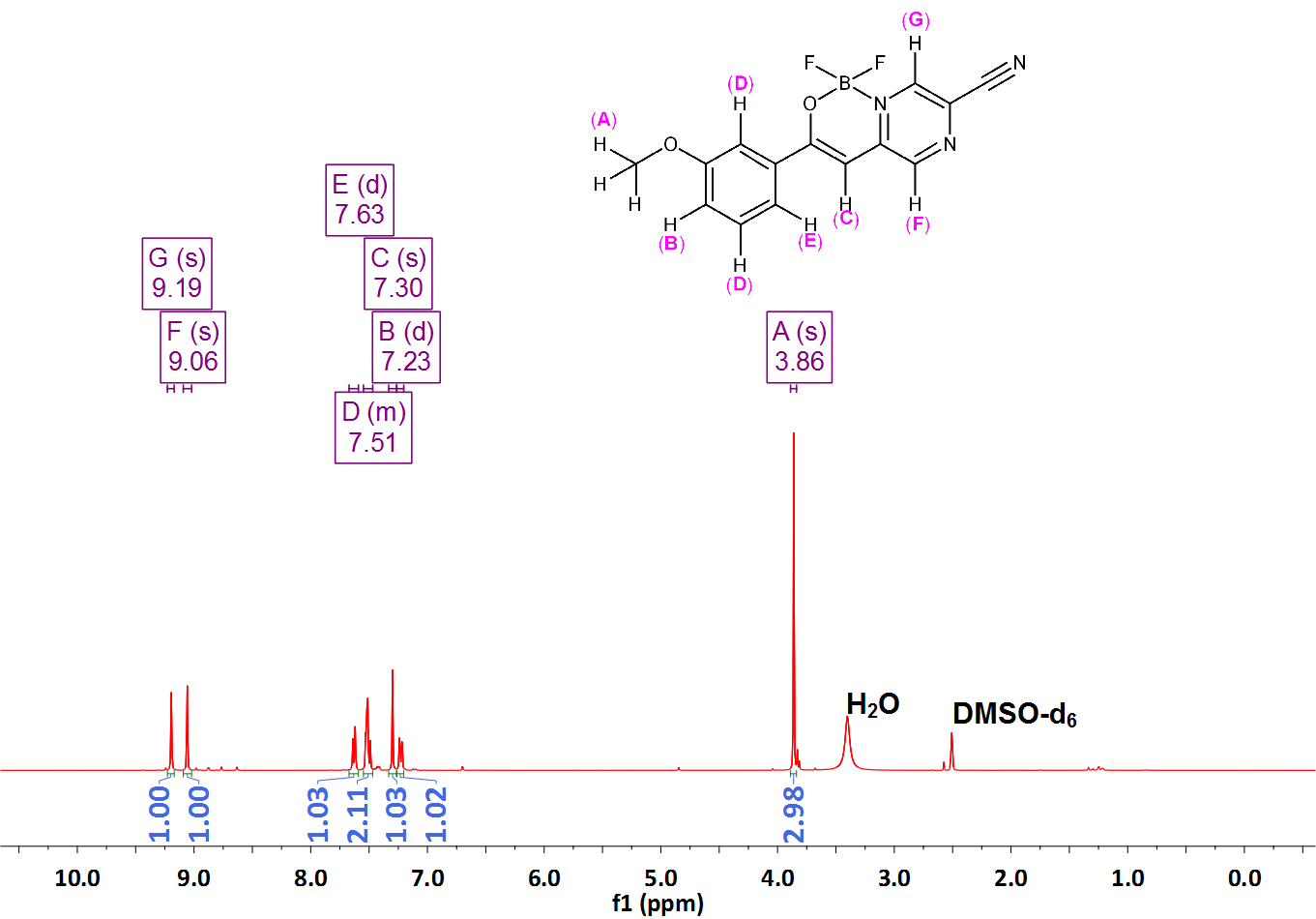
**

**
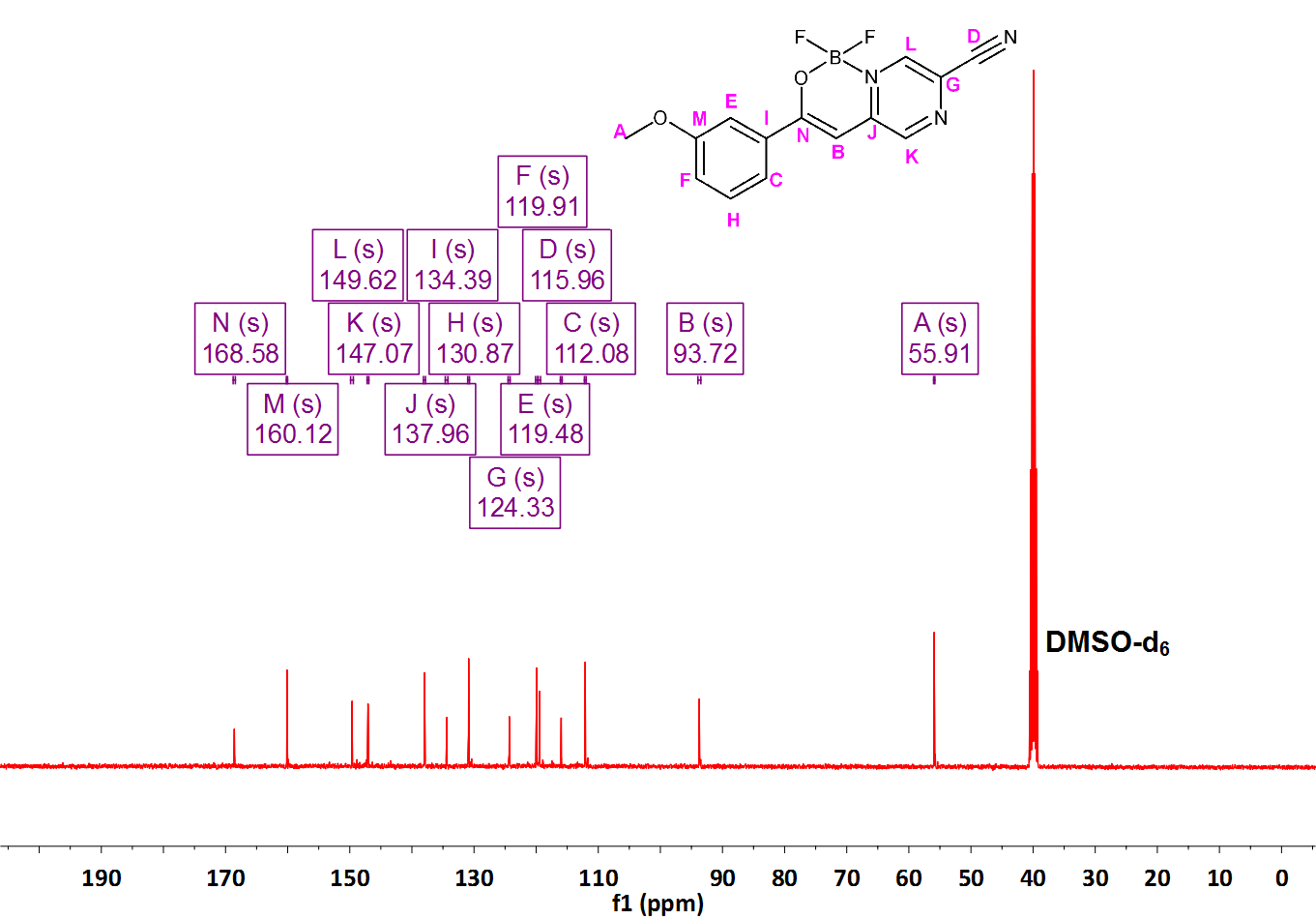
**

**7-cyano-1,1-difluoro-3-(2-methoxyphenyl)-1H-pyrazino[1,2-c][1,3,2]oxazaborinin-9-ium-1-uide (4m)**

**
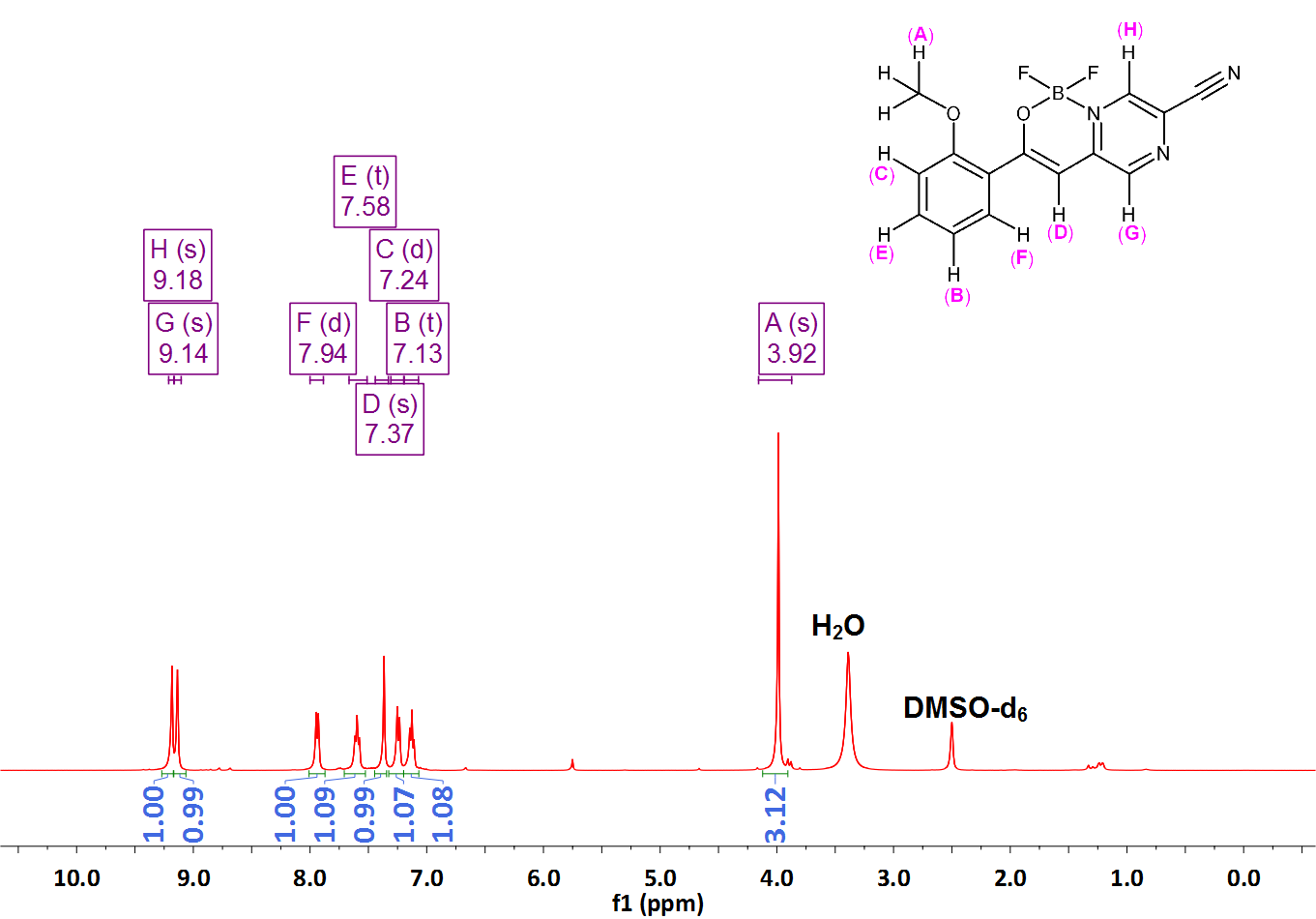
**

**
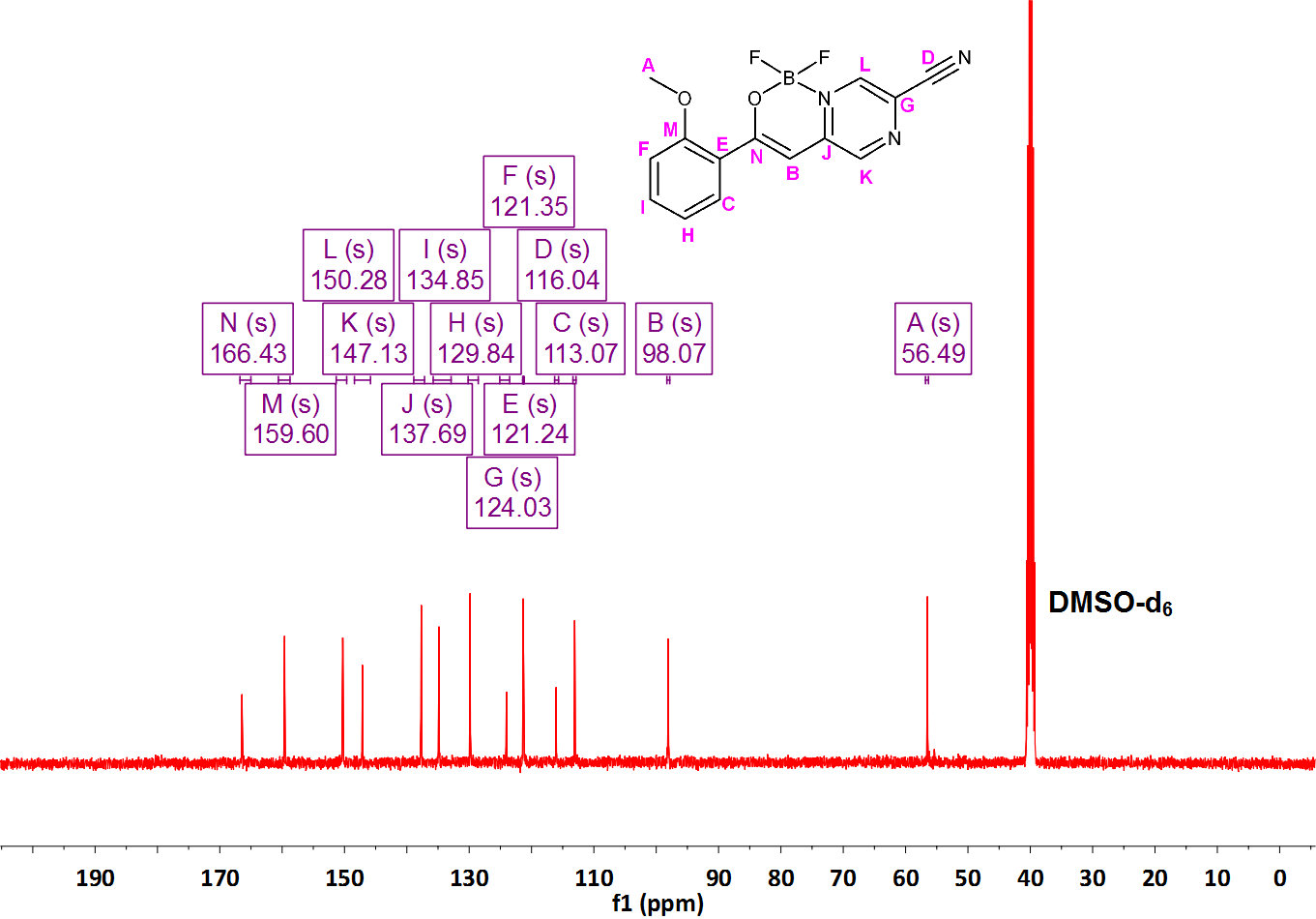
**

**7-cyano-1,1-difluoro-3-(o-tolyl)-1H-pyrazino[1,2-c][1,3,2]oxazaborinin-9-ium-1-uide (4n)**

**
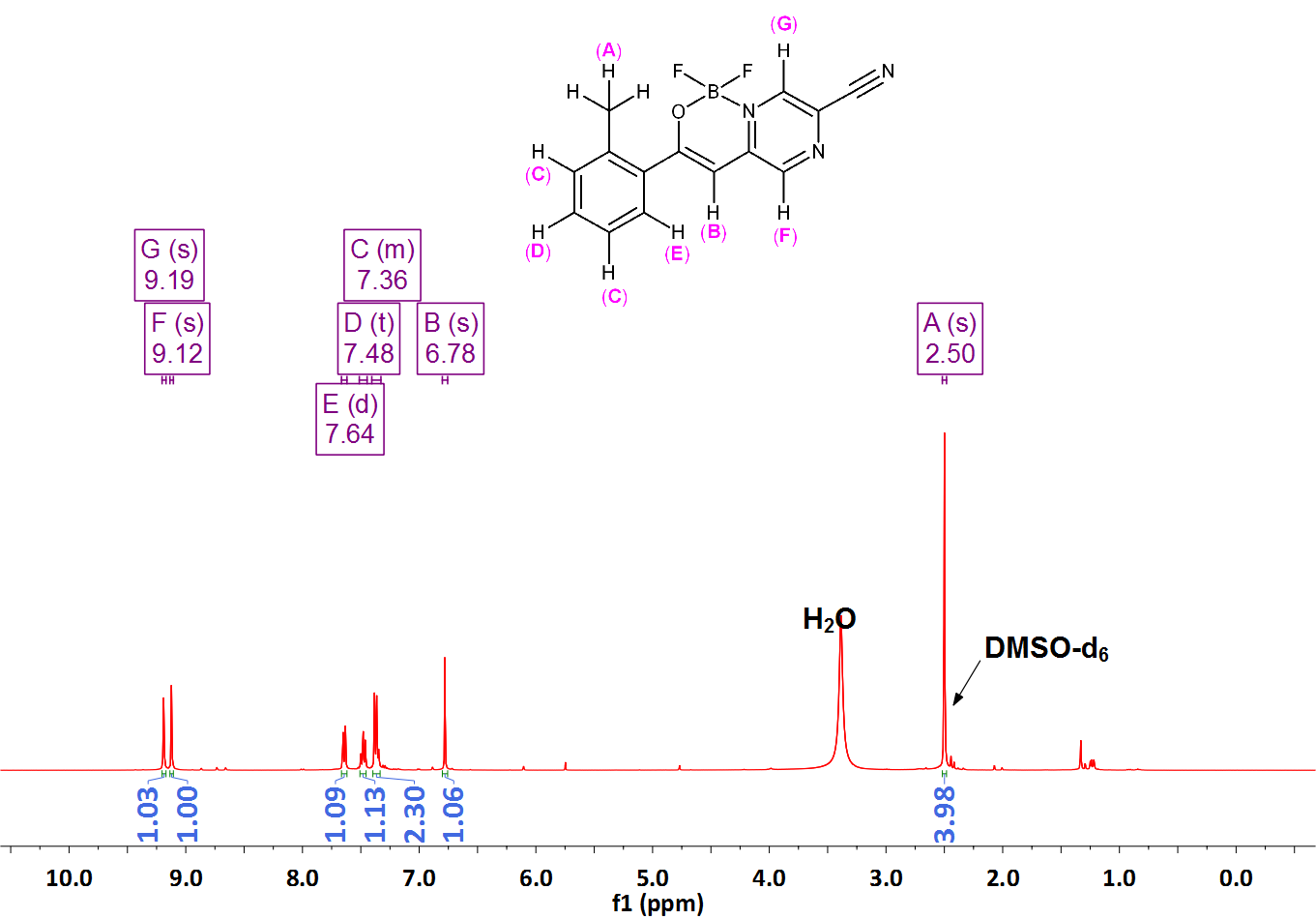
**

**
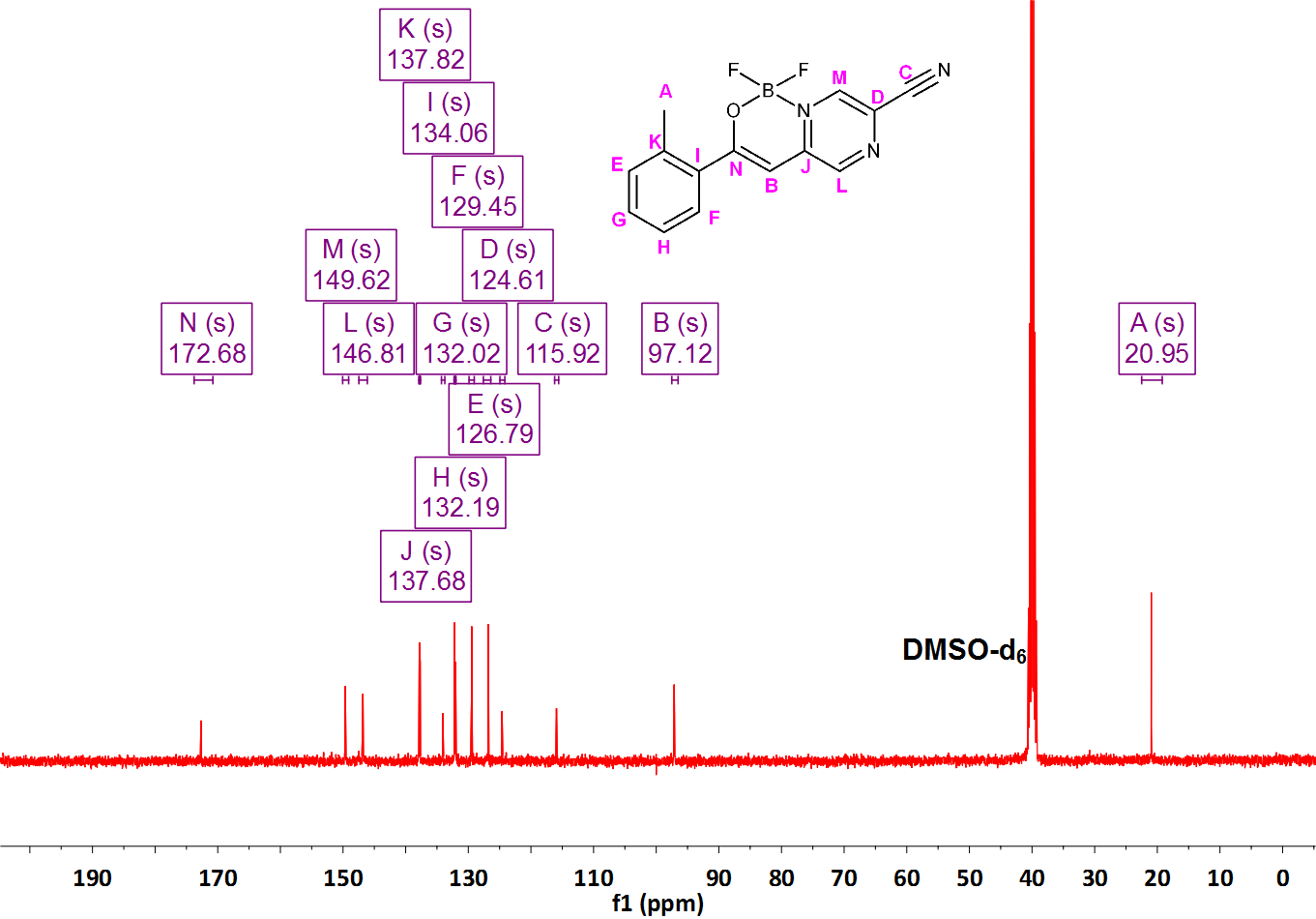
**

**7-cyano-3-(3,5-dimethylphenyl)-1,1-difluoro-1H-pyrazino[1,2-c][1,3,2]oxazaborinin-9-ium-1-uide (4o)**

**
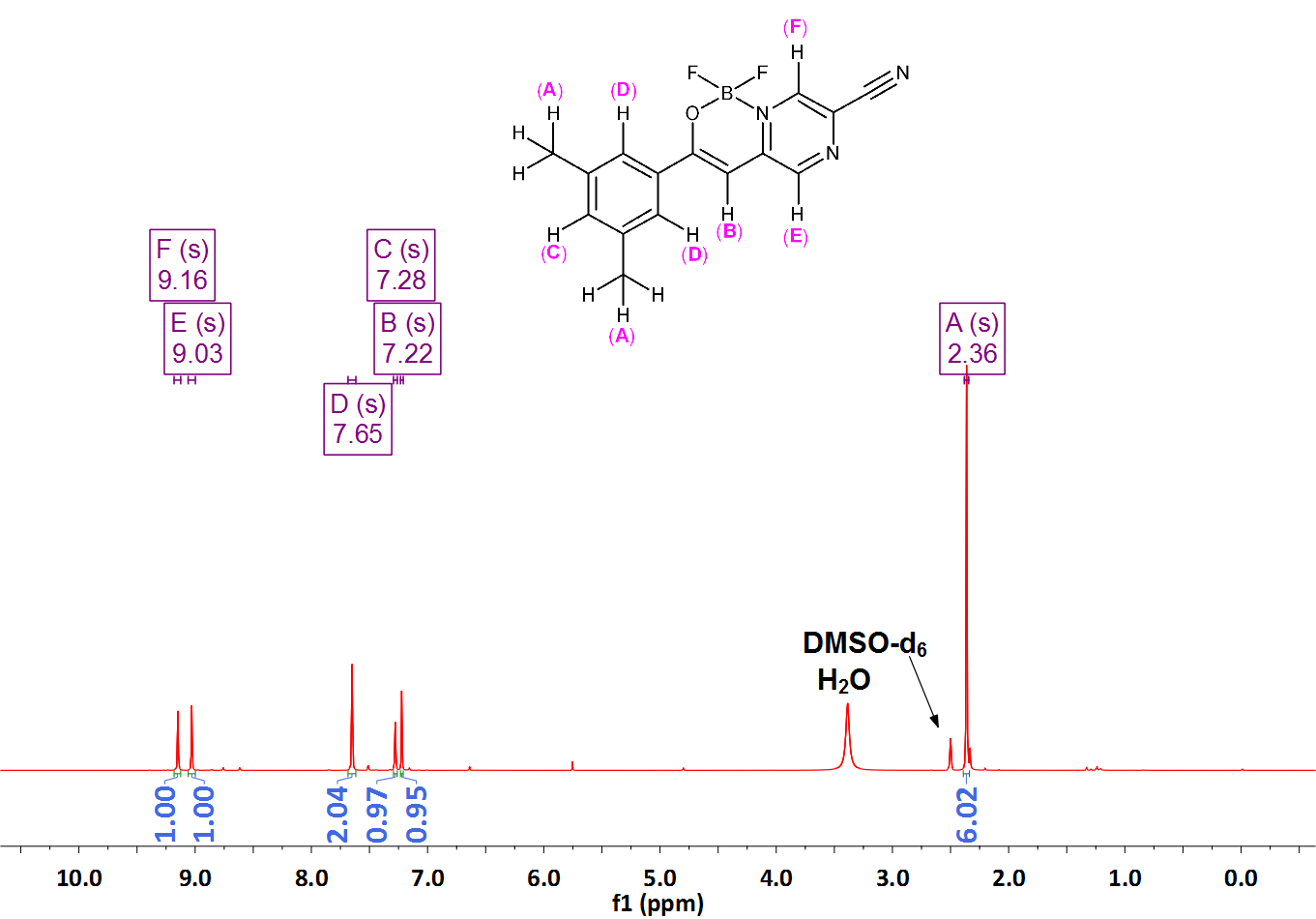
**

**
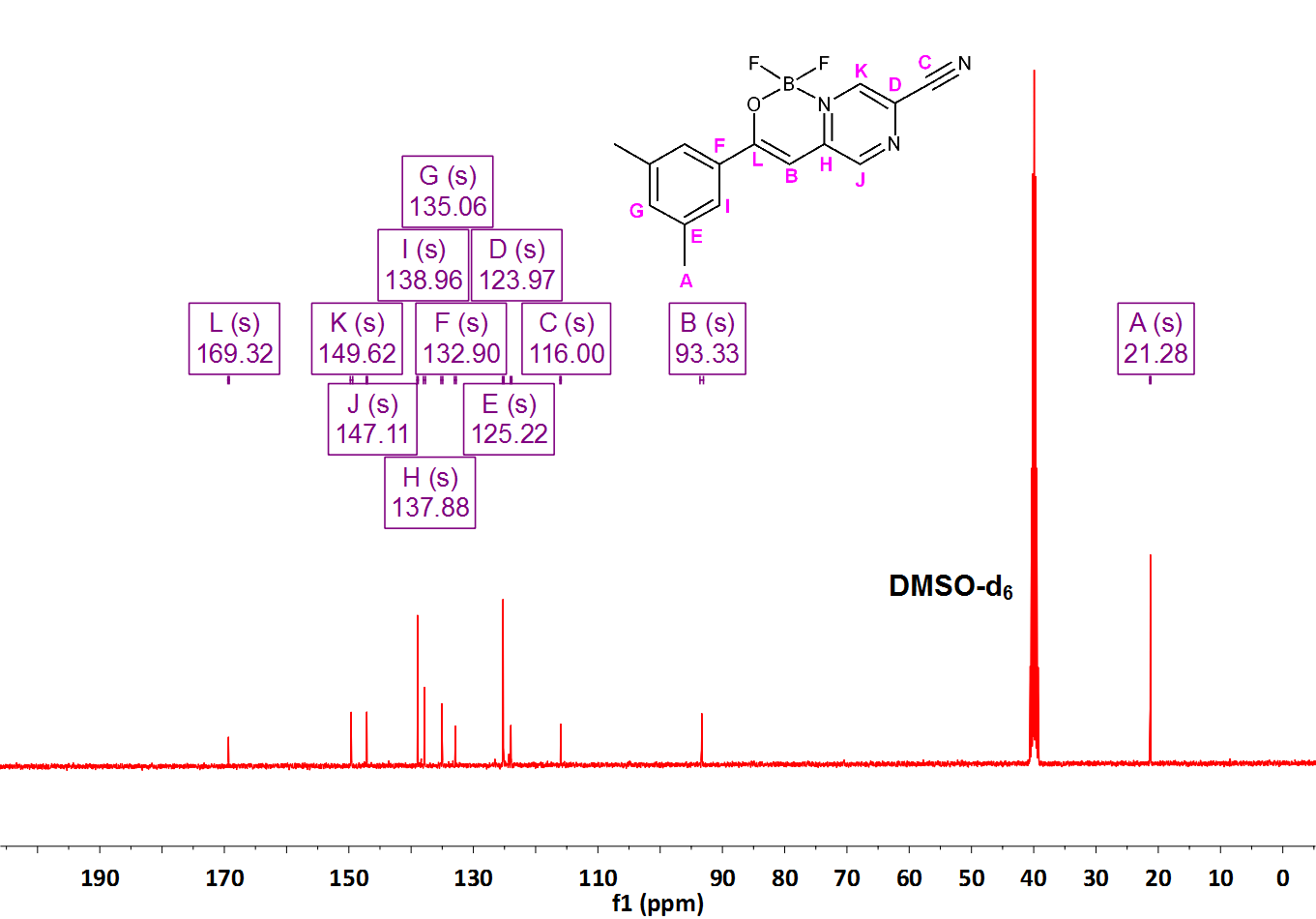
**

**7-cyano-1,1-difluoro-3-mesityl-1H-pyrazino[1,2-c][1,3,2]oxazaborinin-9-ium-1-uide (4p)**

**
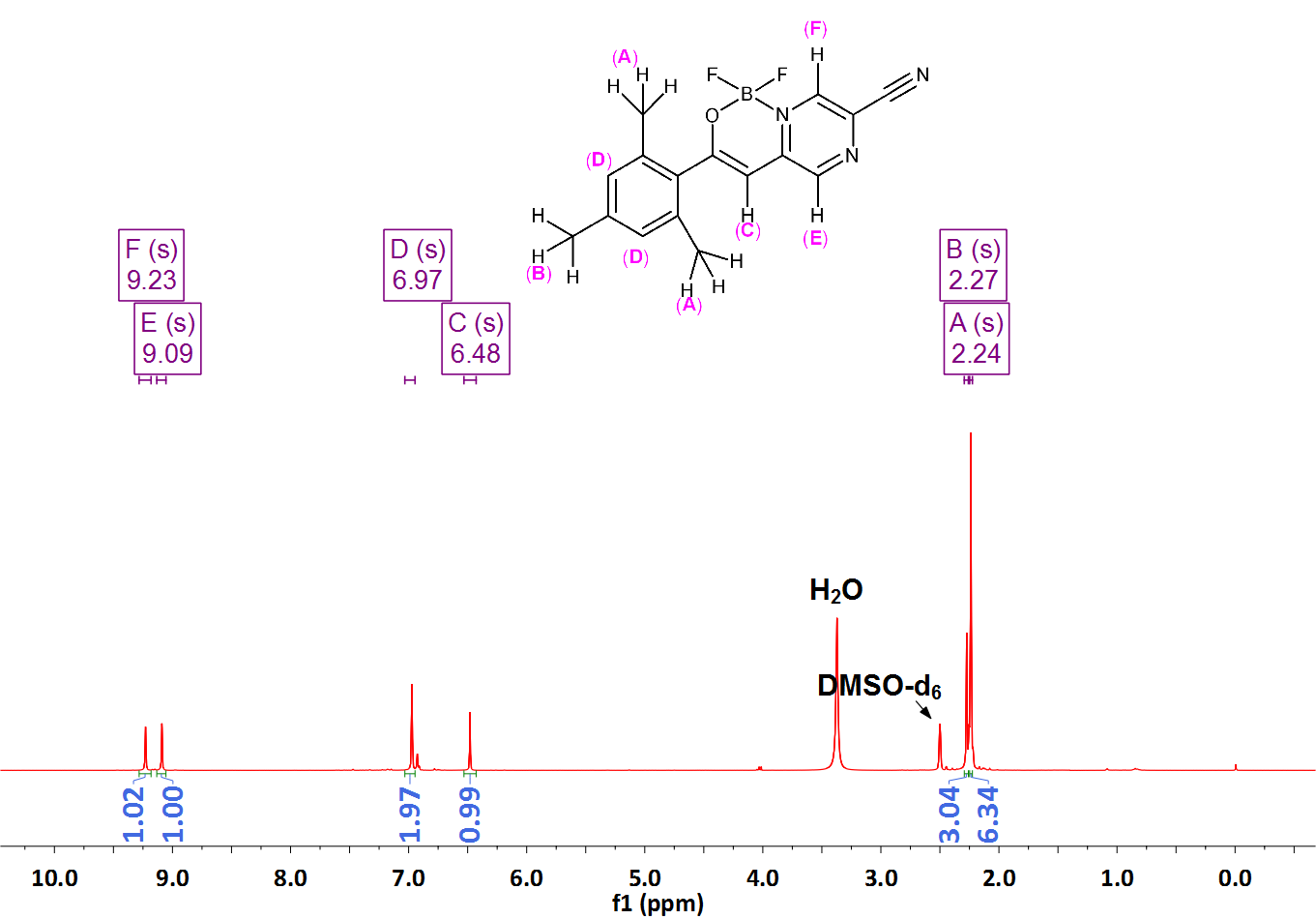
**

**
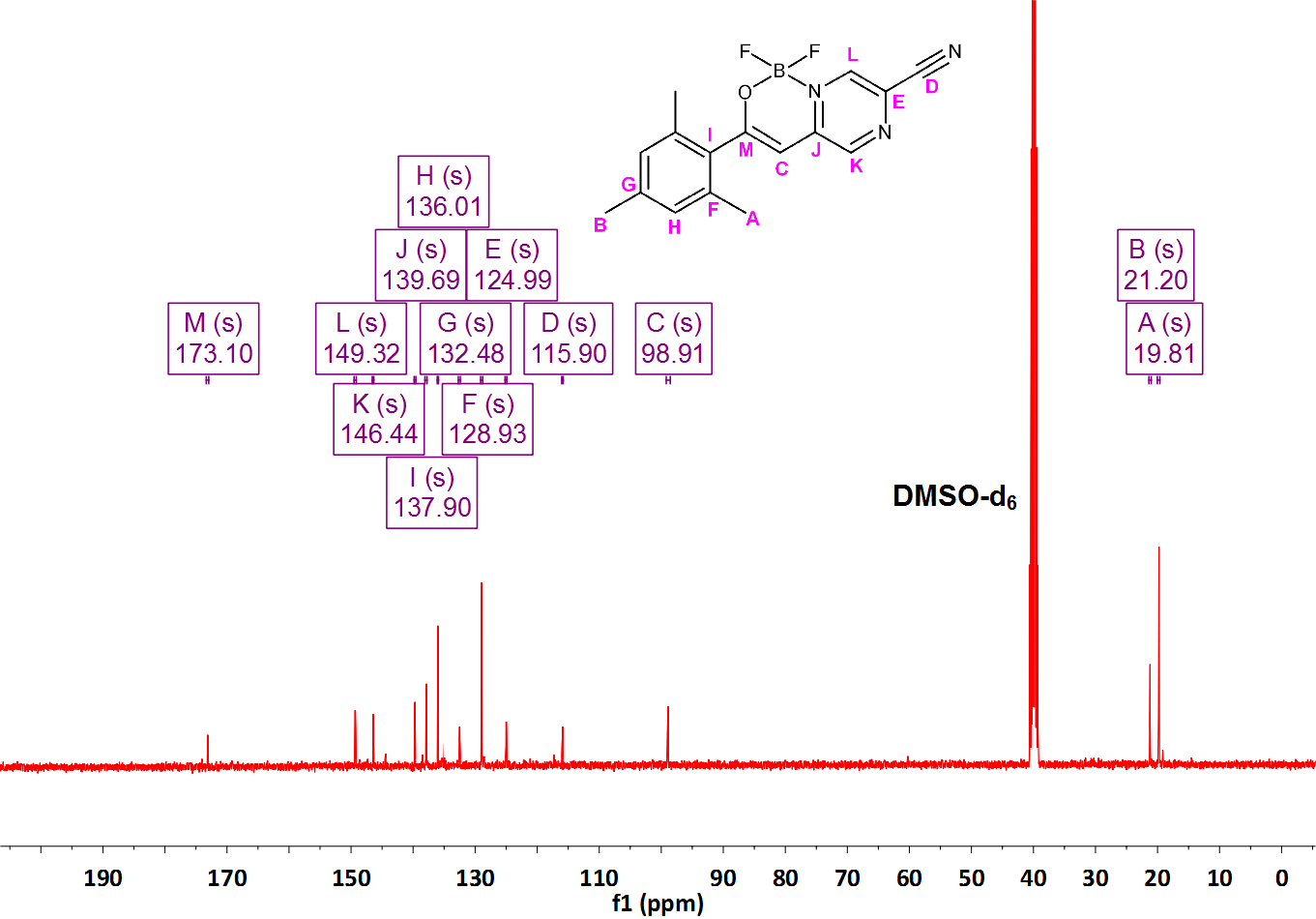
**

**3-(benzo[d][1,3]dioxol-5-yl)-7-cyano-1,1-difluoro-1H-pyrazino[1,2-c][1,3,2]oxazaborinin-9-ium-1-uide (4q)**

**
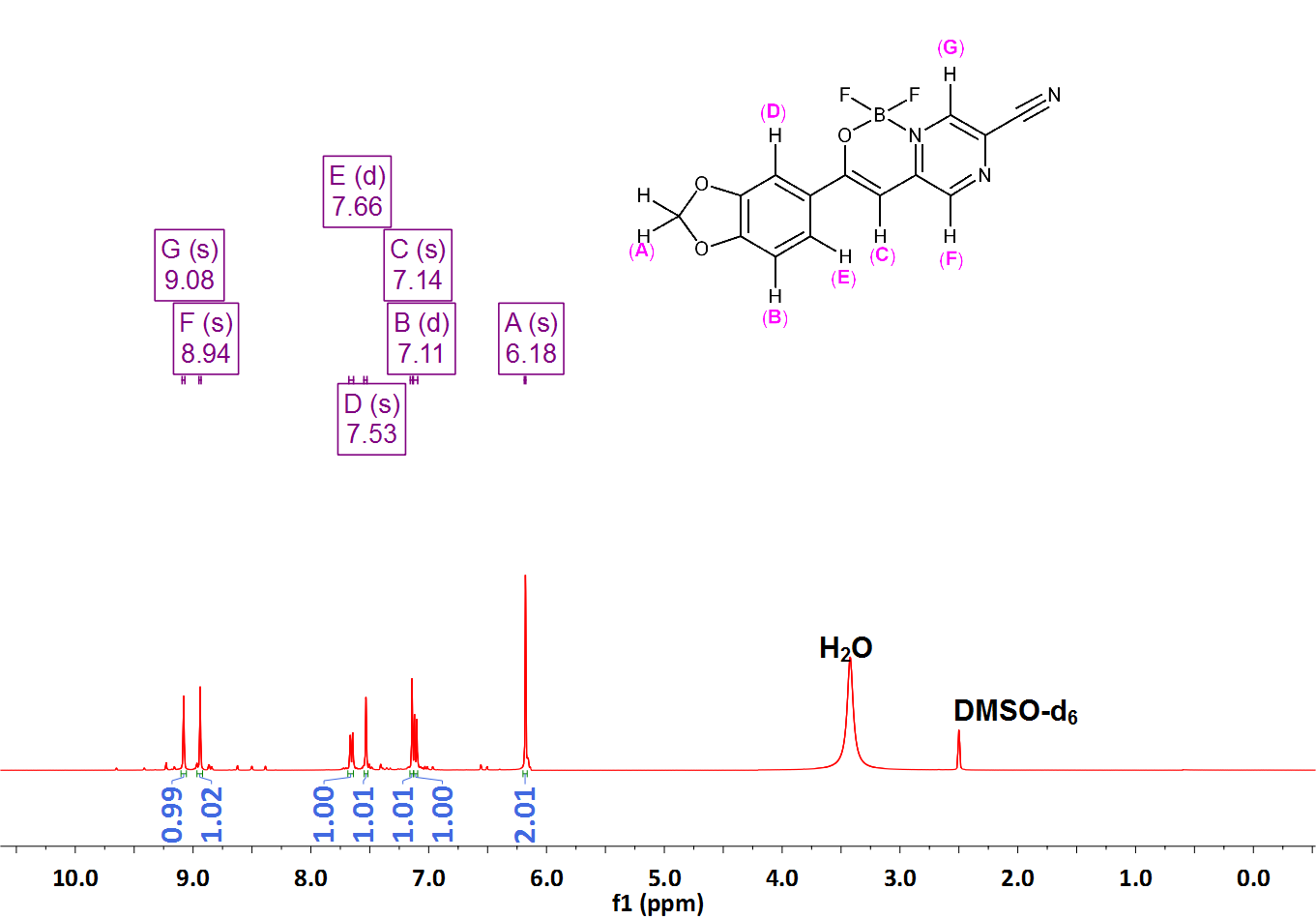
**

**
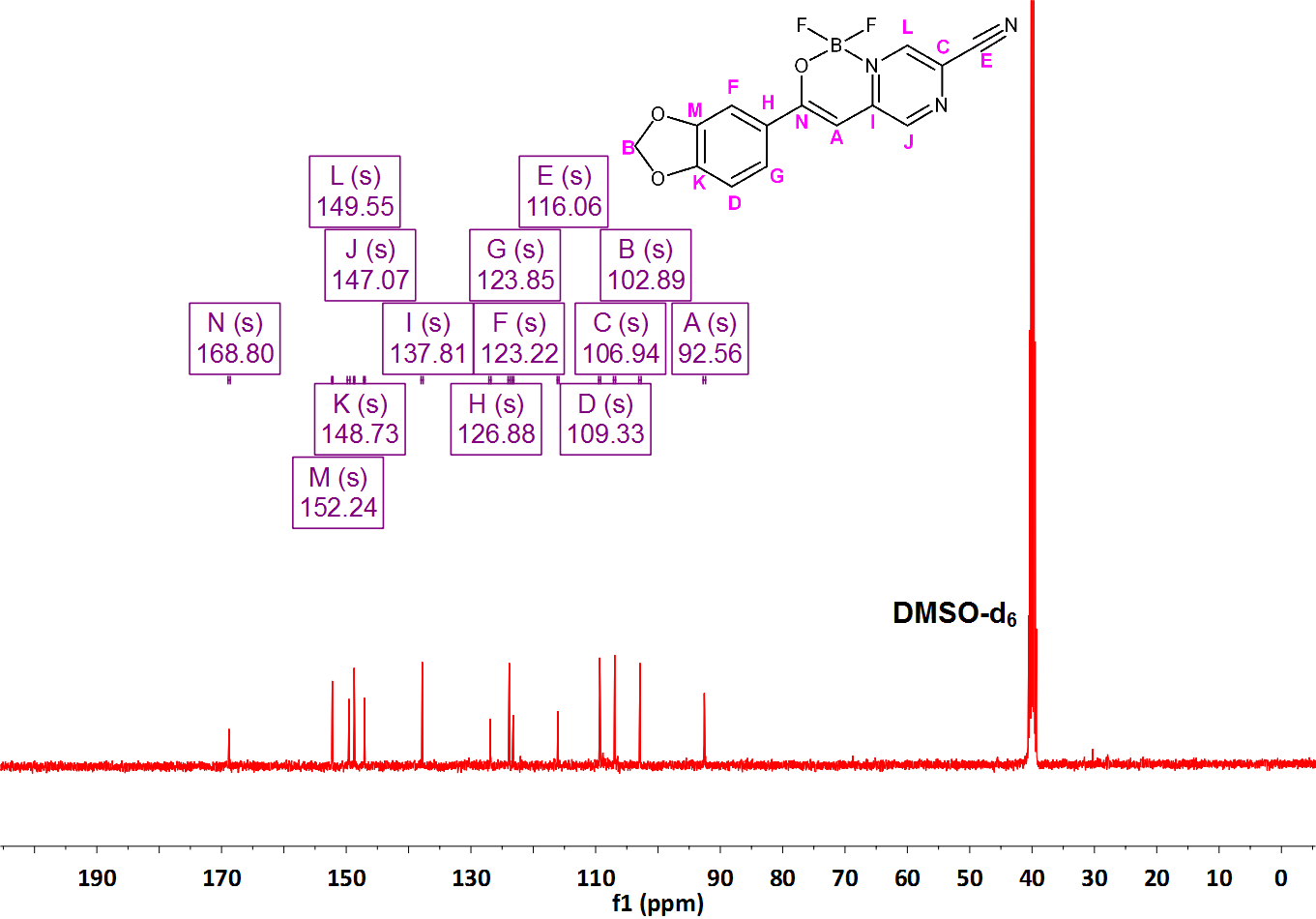
**

**7-cyano-1,1-difluoro-3-(naphthalen-2-yl)-1H-pyrazino[1,2-c][1,3,2]oxazaborinin-9-ium-1-uide (4r)**

**
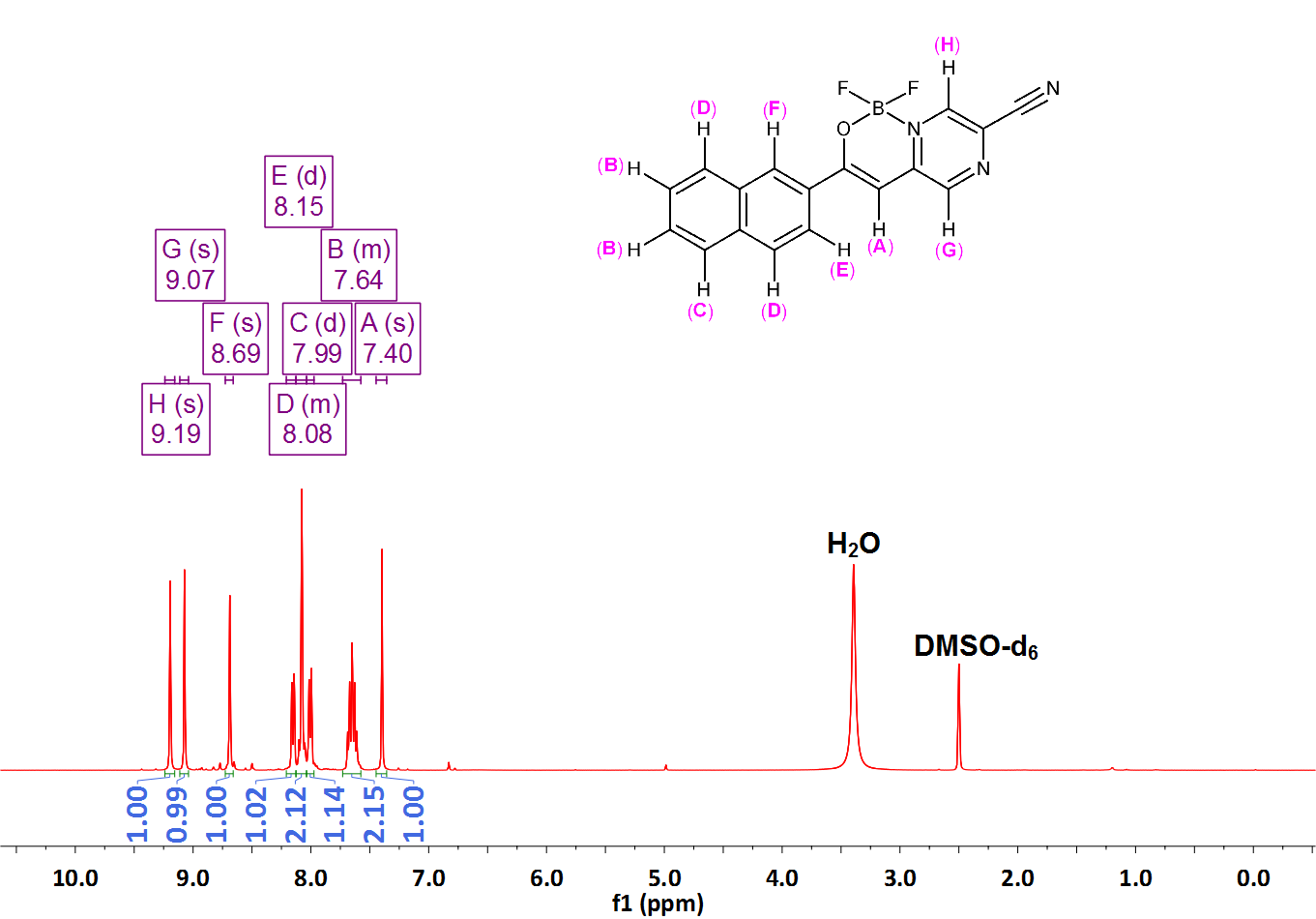
**

**
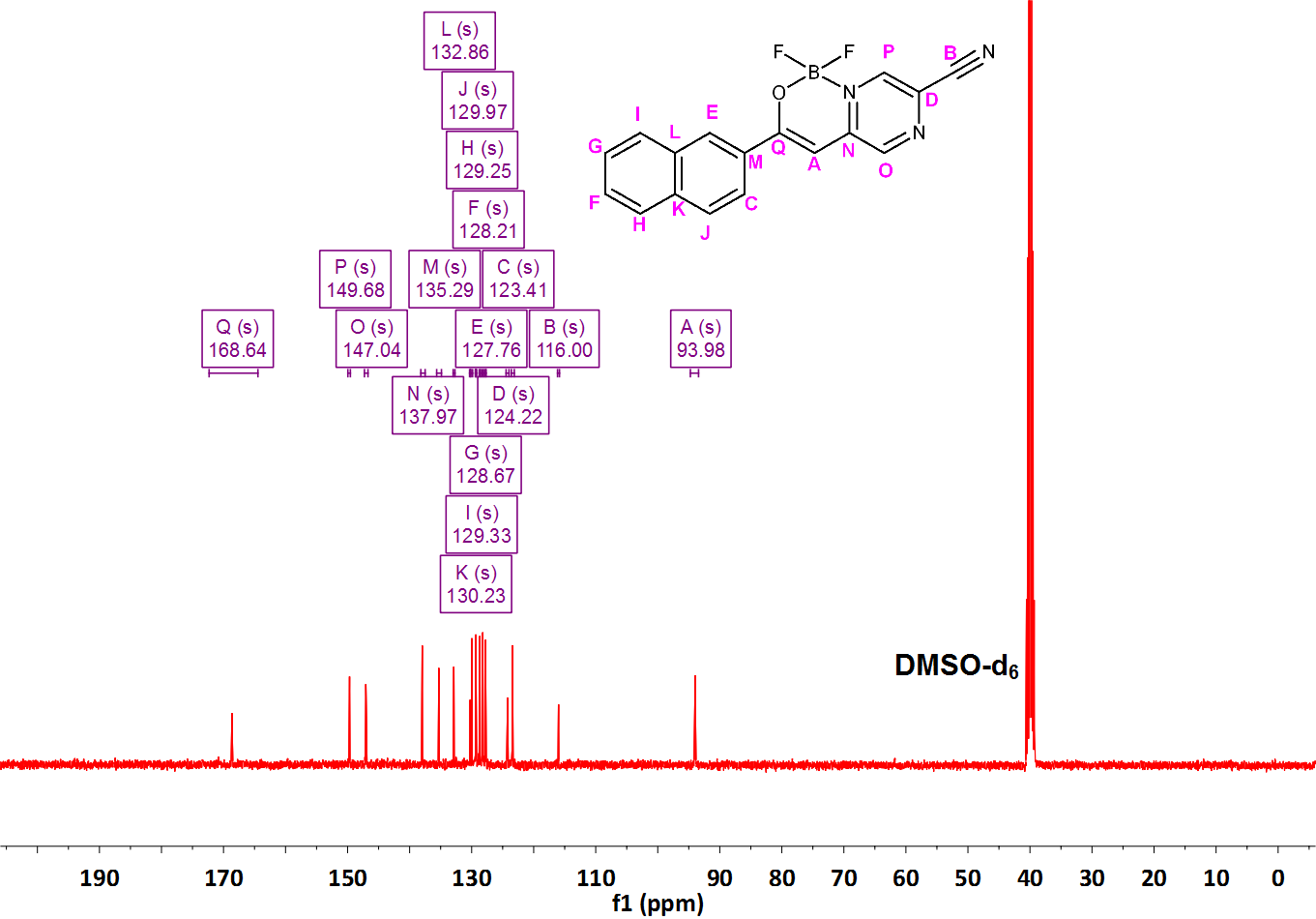
**

**2-cyano-12,12-difluoro-6,12-dihydro-5H-naphtho[2,1-e]pyrazino[1,2-c][1,3,2]oxazaborinin-13-ium-12-uide (4s)**

**
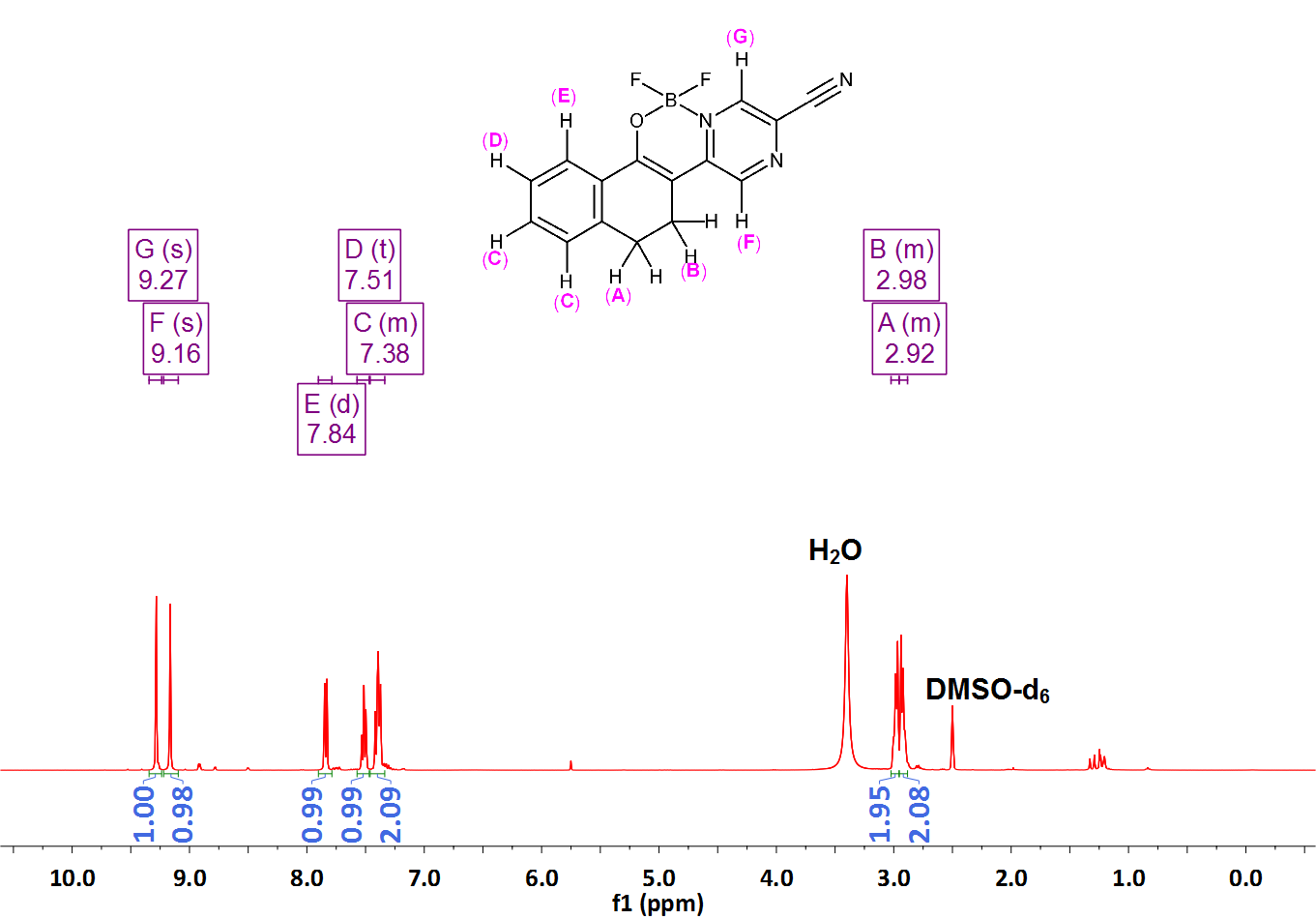
**

**
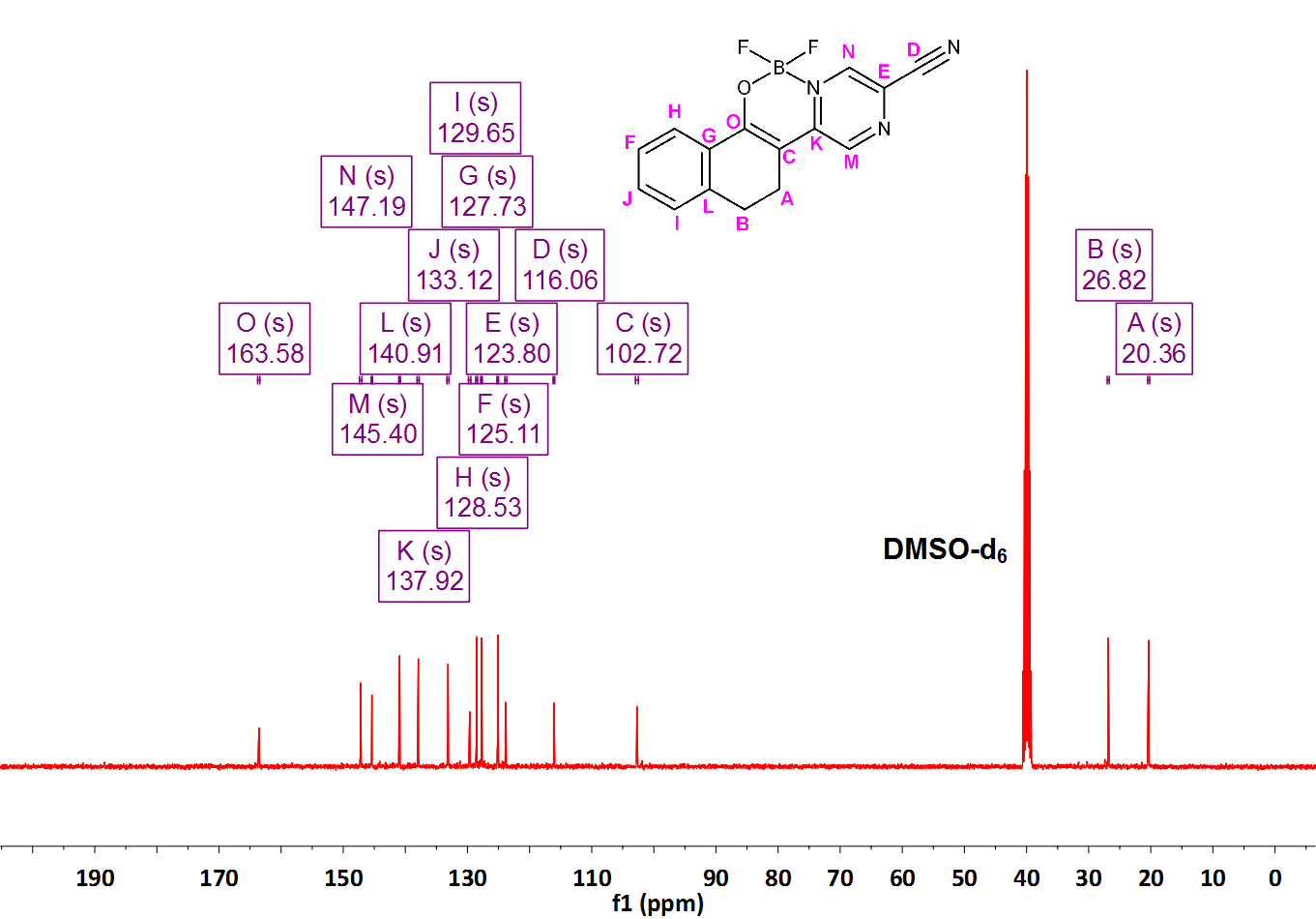
**

**2-cyano-13,13-difluoro-5,6,7,13-tetrahydrobenzo[3,4]cyclohepta[1,2-e]pyrazino[1,2-c][1,3,2]oxazaborinin-14-ium-13-uide (4t)**

**
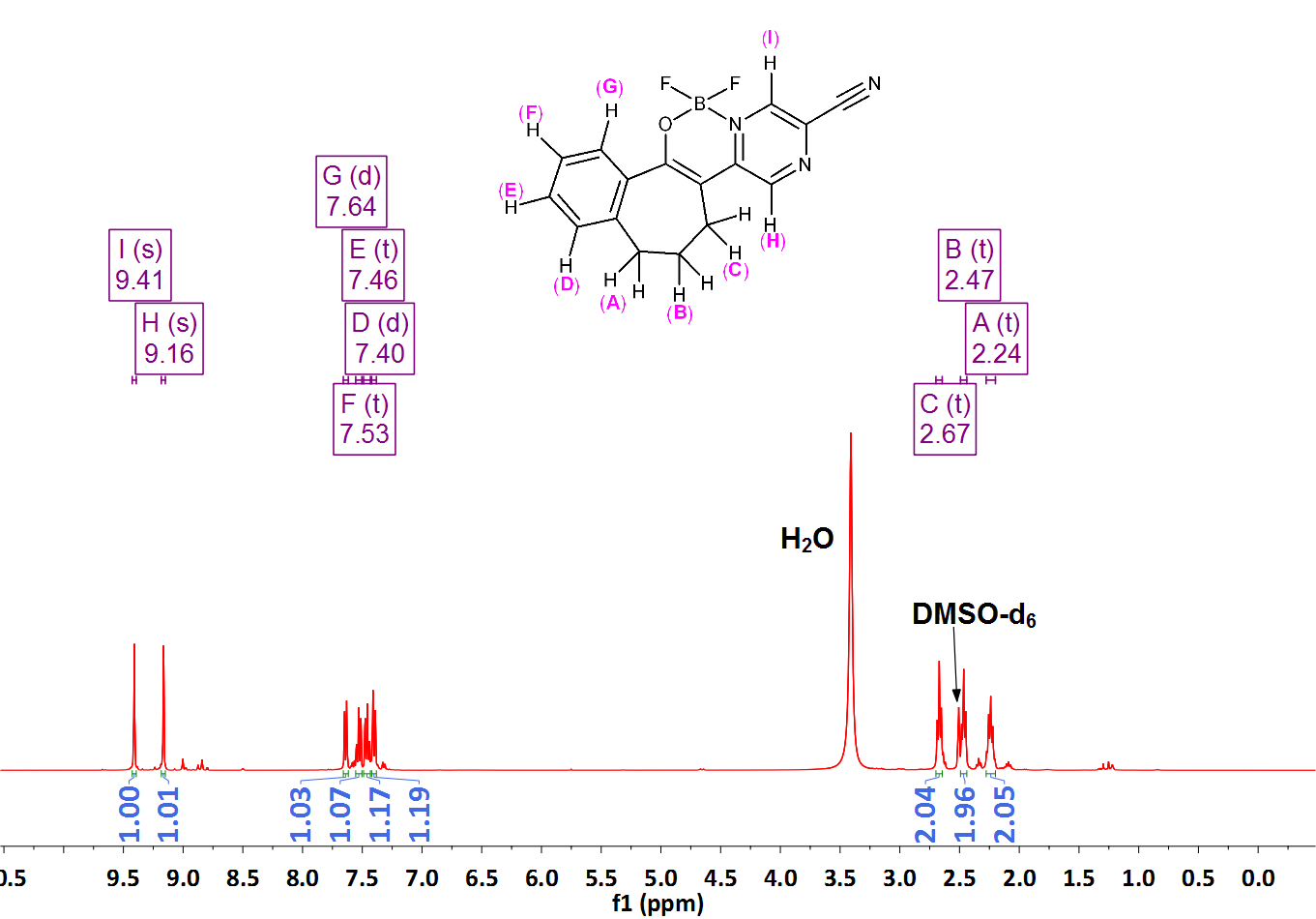
**

**
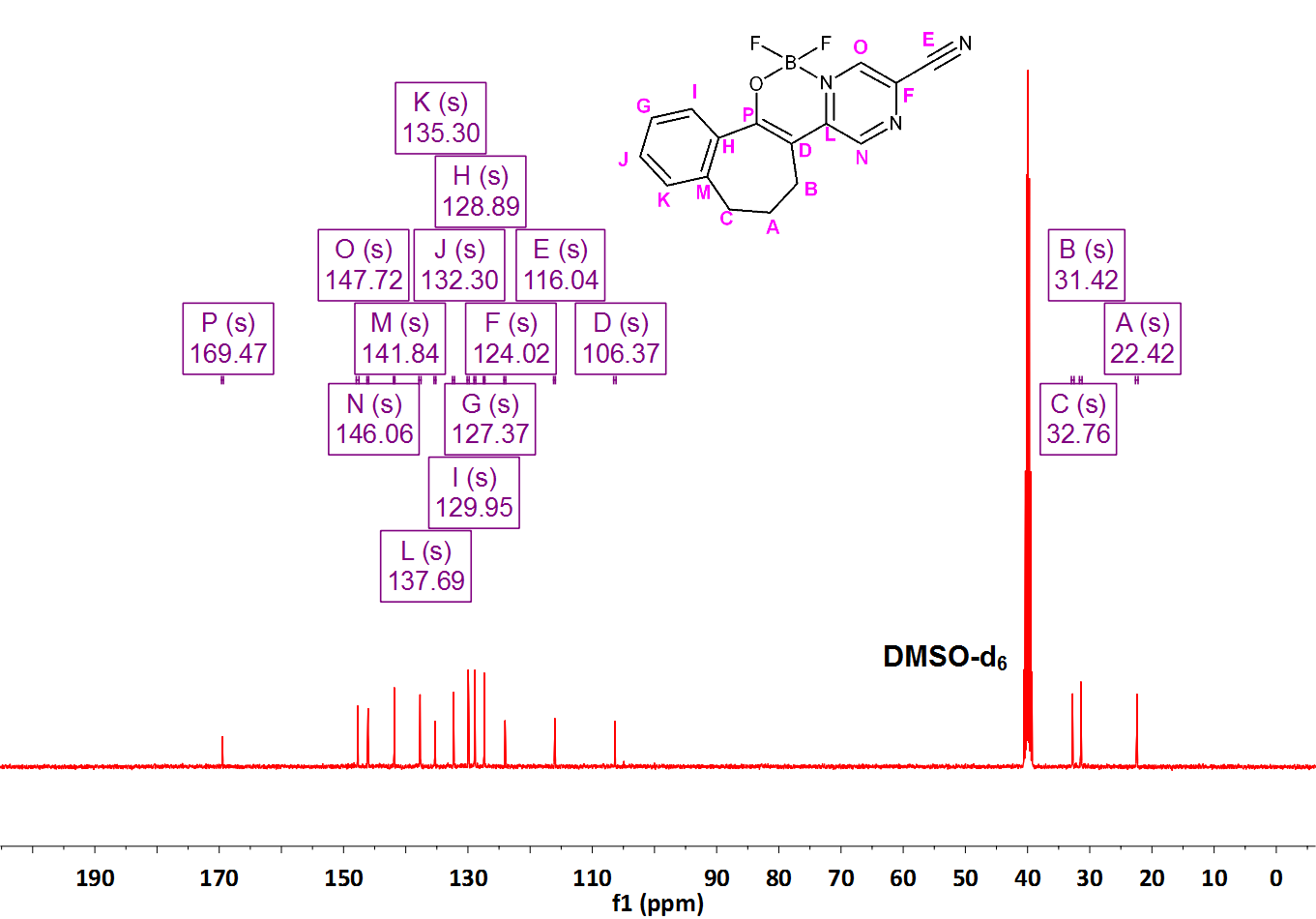
**

**7-cyano-1,1-difluoro-3-(thiophen-2-yl)-1H-pyrazino[1,2-c][1,3,2]oxazaborinin-9-ium-1-uide (4u)**

**
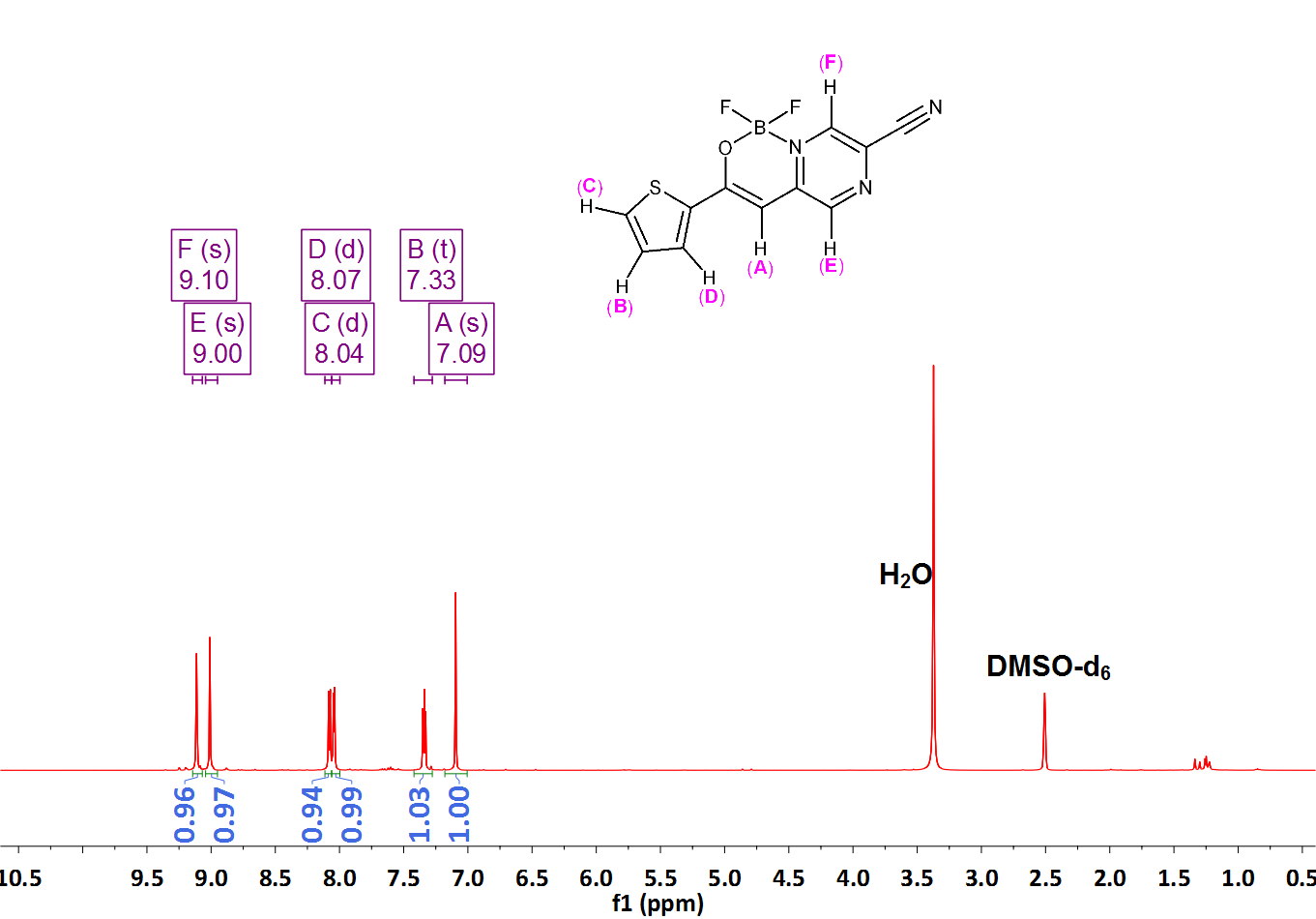
**

**
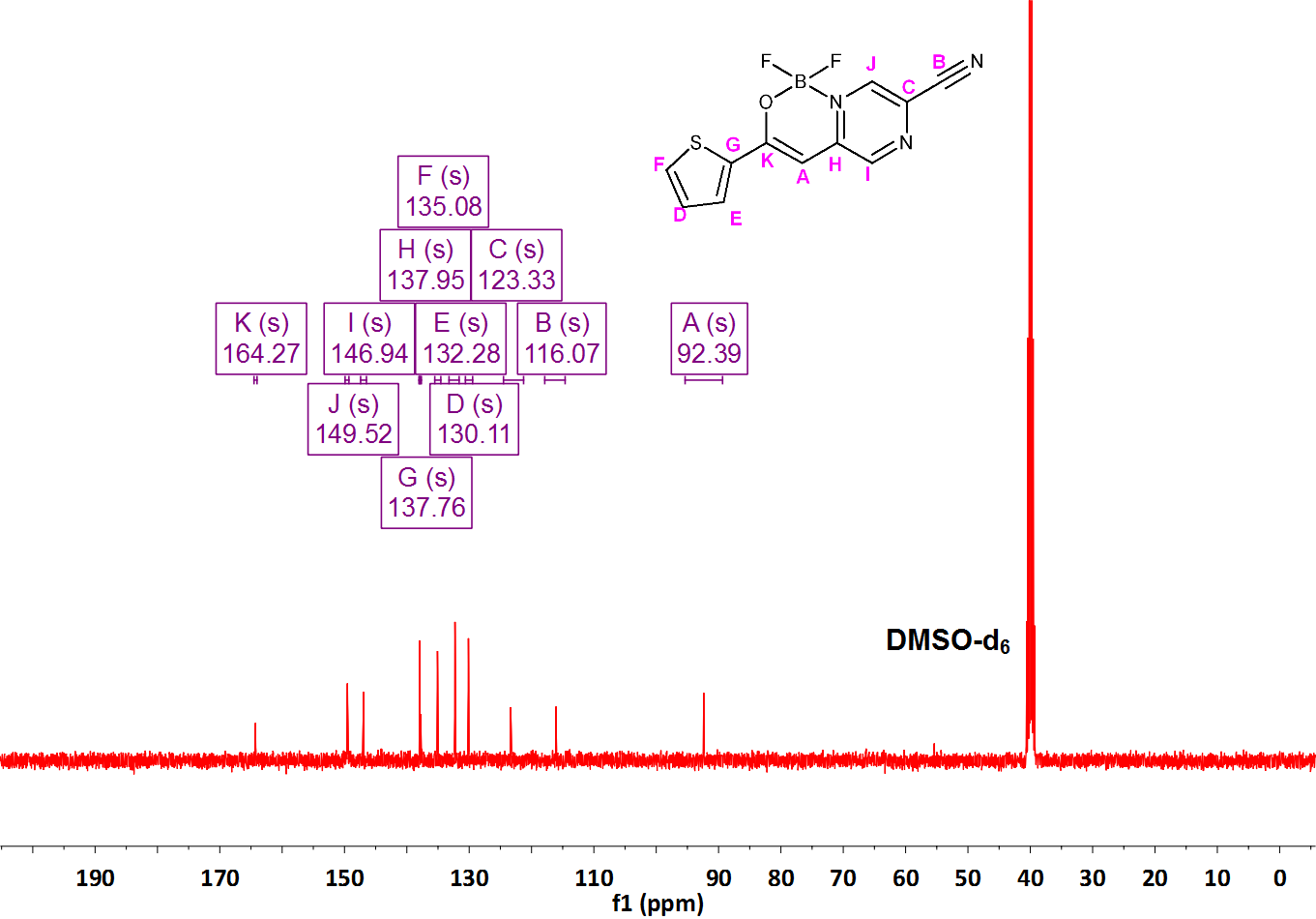
**

**7-cyano-3-(4-(9,9-dimethyl-9H-fluoren-2-yl)phenyl)-1,1-difluoro-1H-pyrazino[1,2-c][1,3,2] oxazaborinin-9-ium-1-uide (4aa)**

**
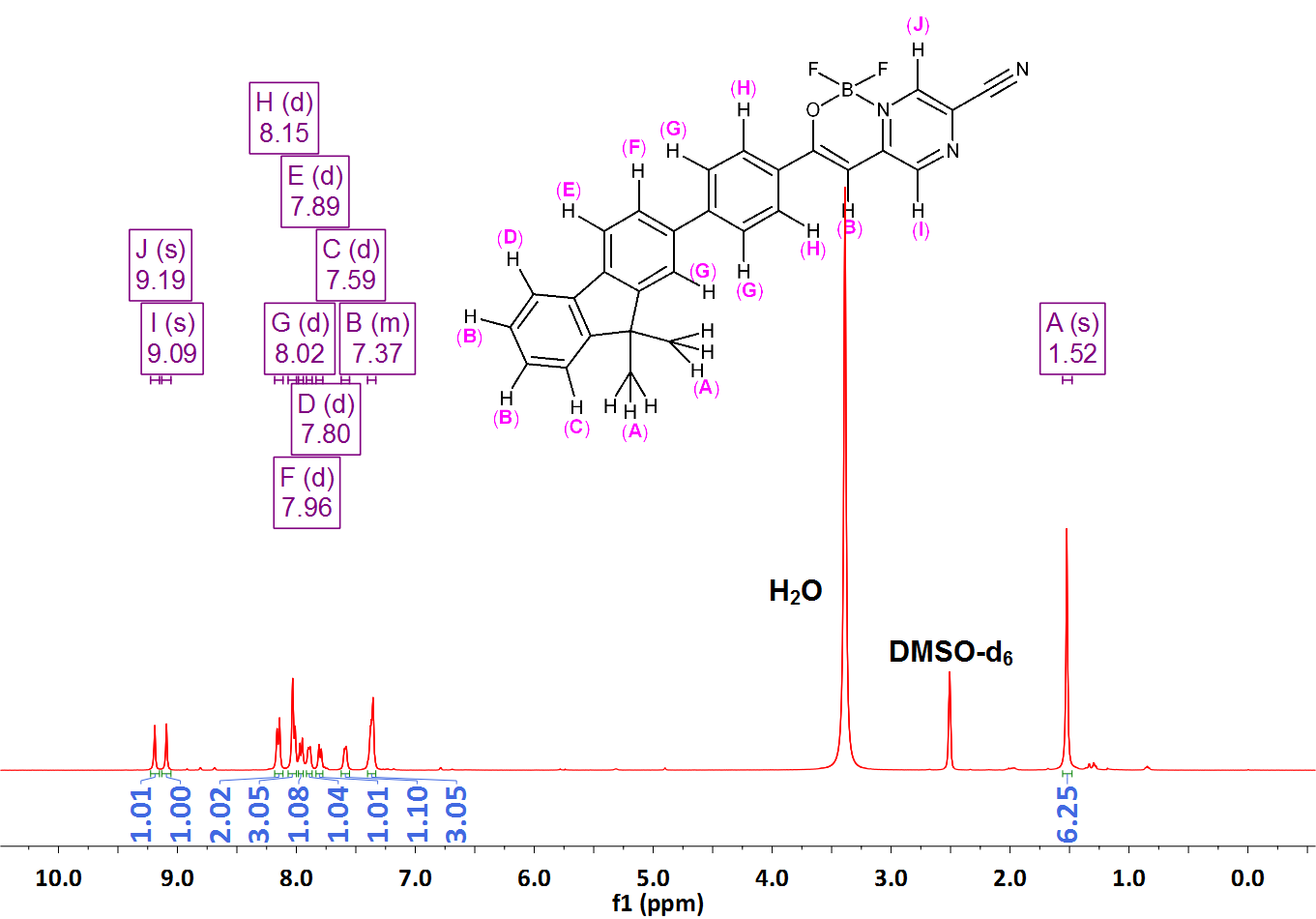
**

**
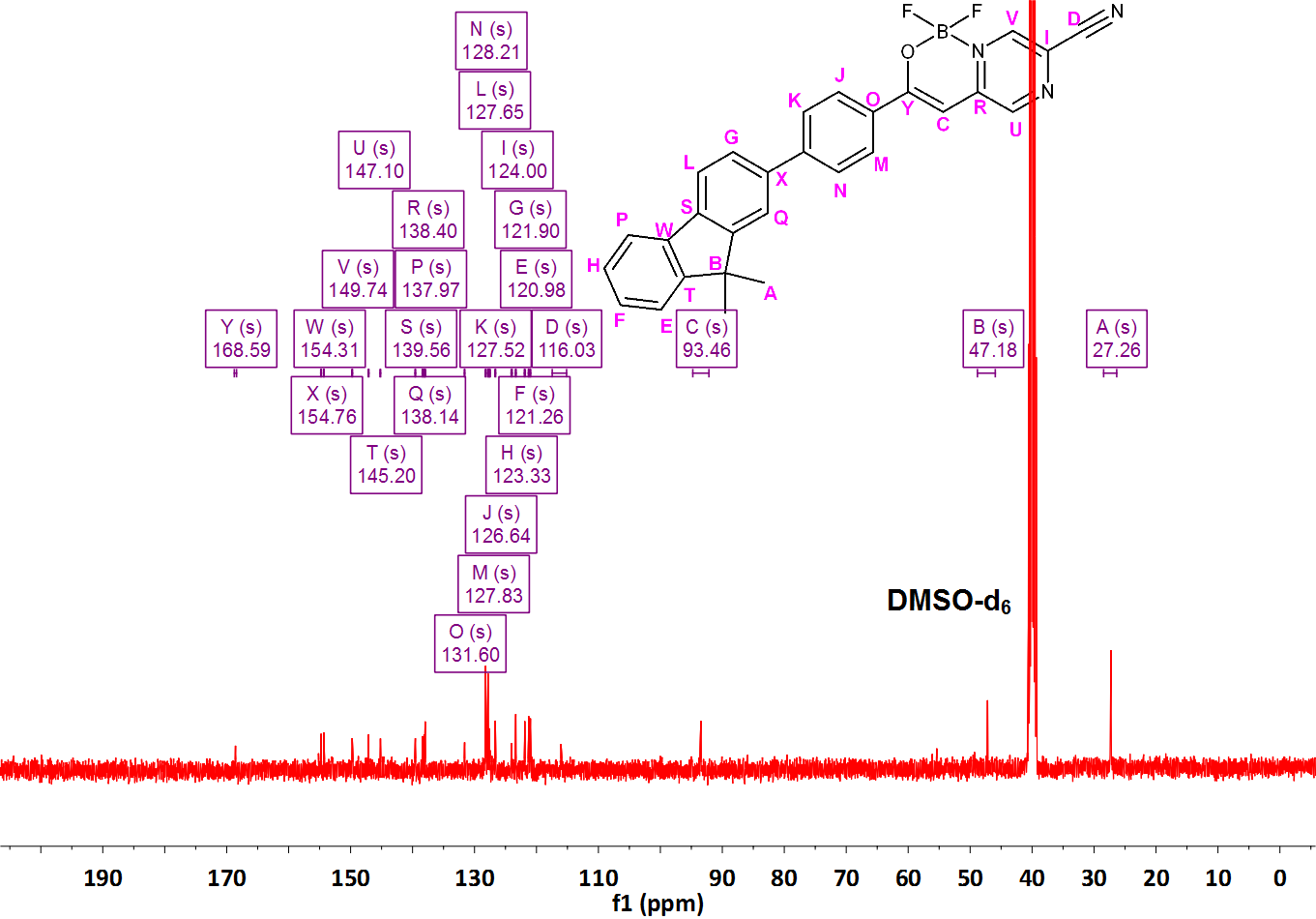
**

**7-cyano-3-(5-(9,9-dimethyl-9H-fluoren-2-yl)thiophen-2-yl)-1,1-difluoro-1H-pyrazino[1,2-c][1,3,2]oxazaborinin-9-ium-1-uide (4ab)**

**
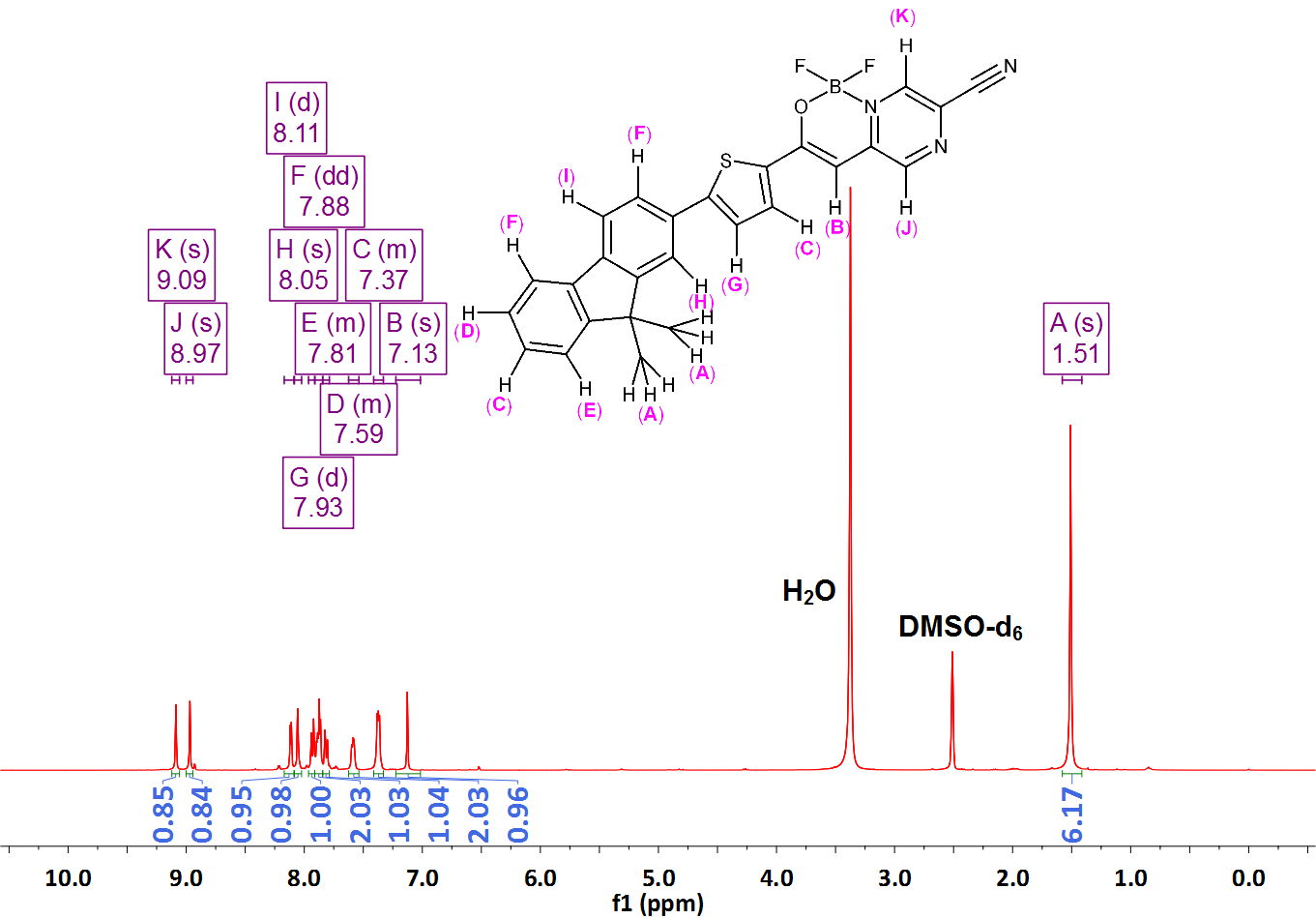
**

**
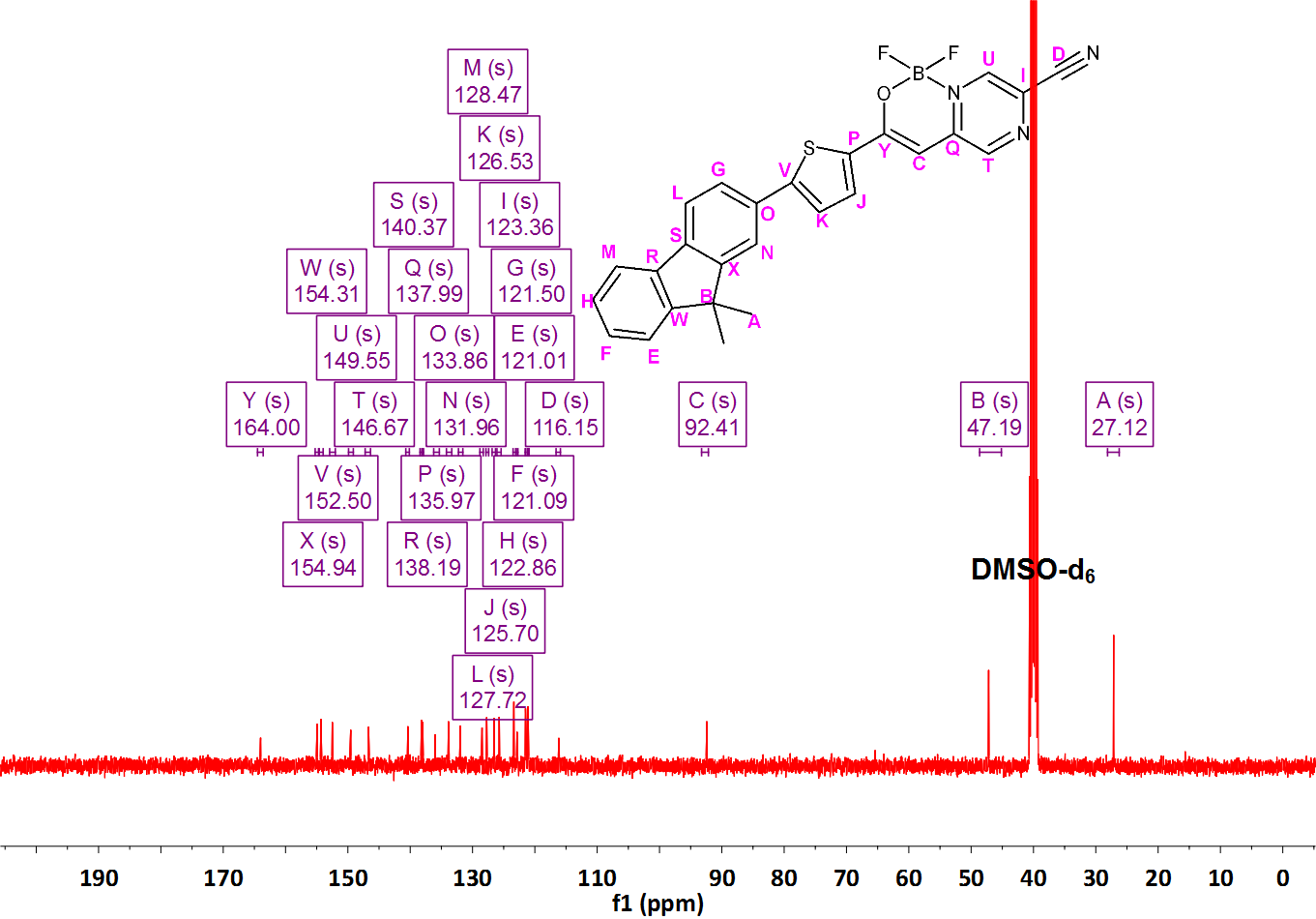
**

**7-cyano-1,1-difluoro-3-(4-(octyloxy)phenyl)-1H-pyrazino[1,2-c][1,3,2]oxazaborinin-9-ium-1-uide(4ac)**

**
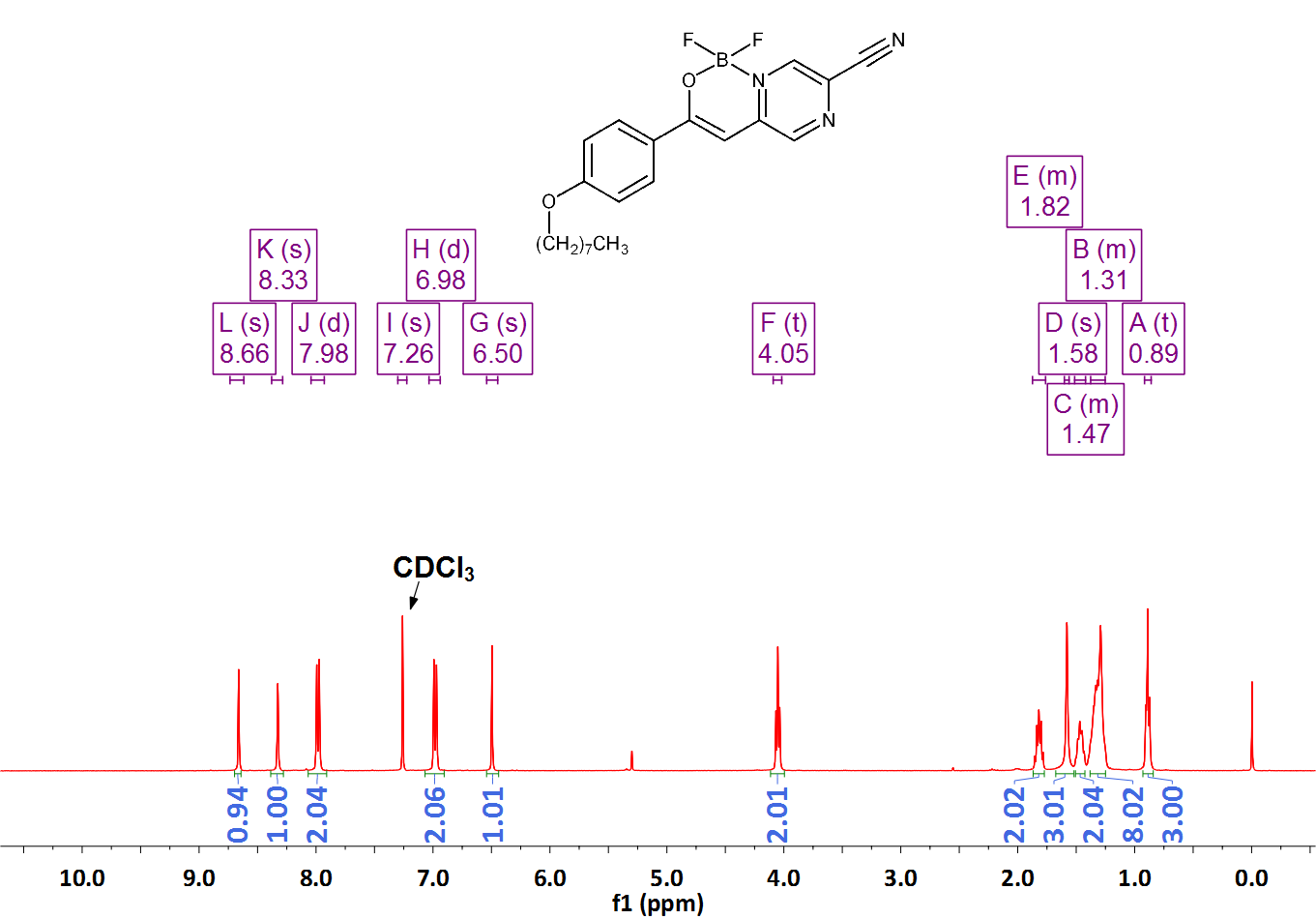
**

**
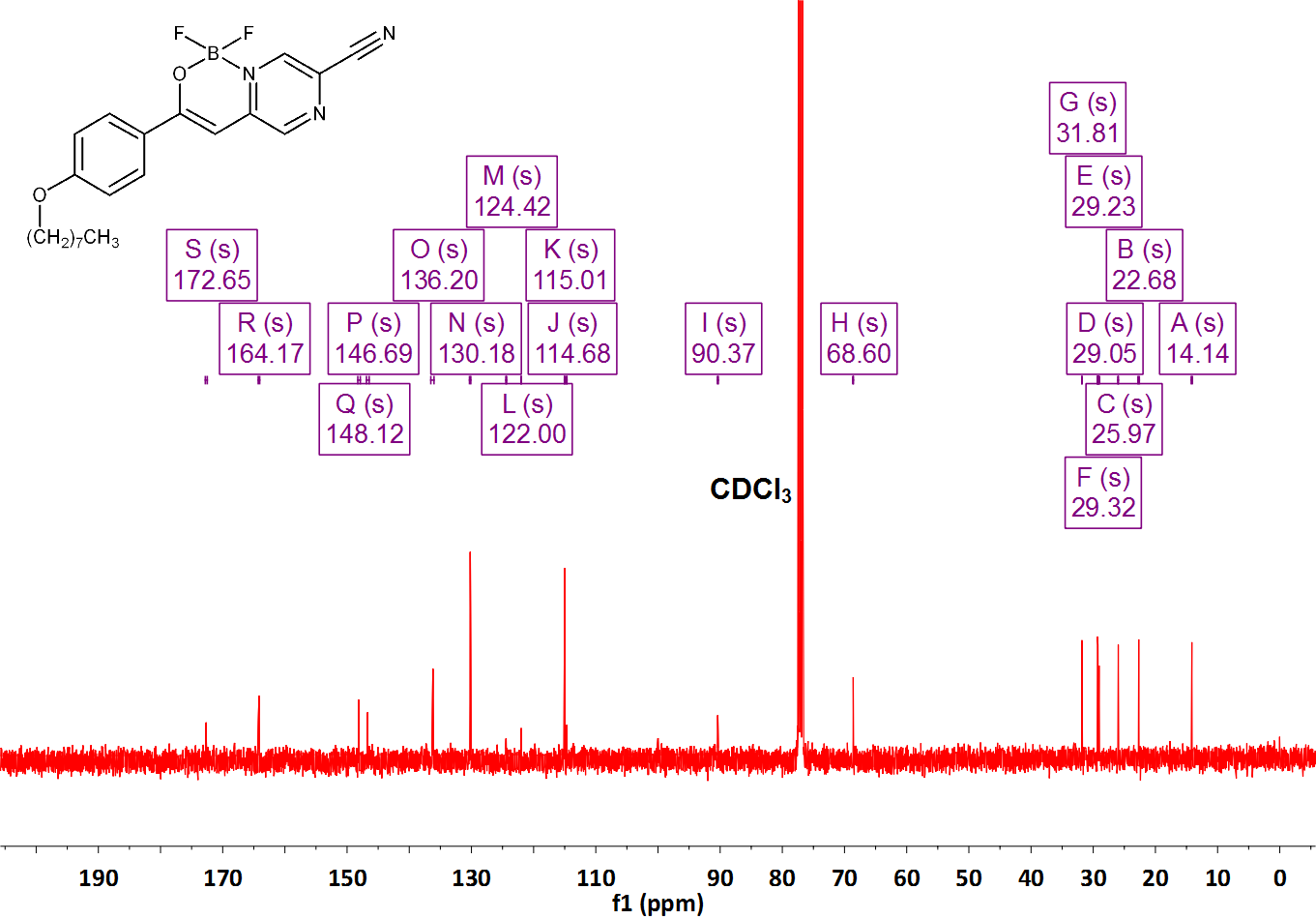
**

**1-(4-(9,9-dimethyl-9H-fluoren-2-yl)phenyl)ethanone (4a_1_)**

**
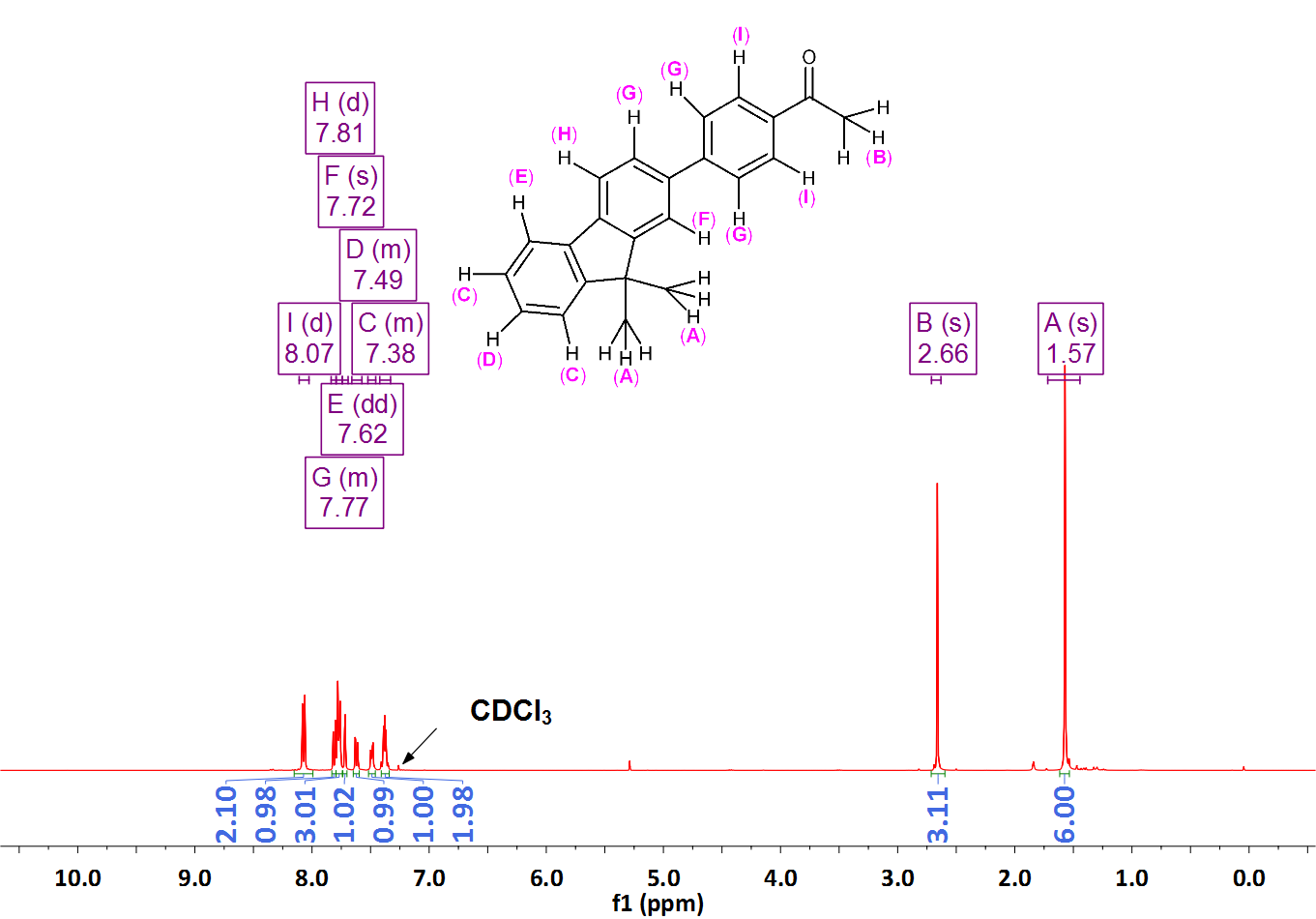
**

**
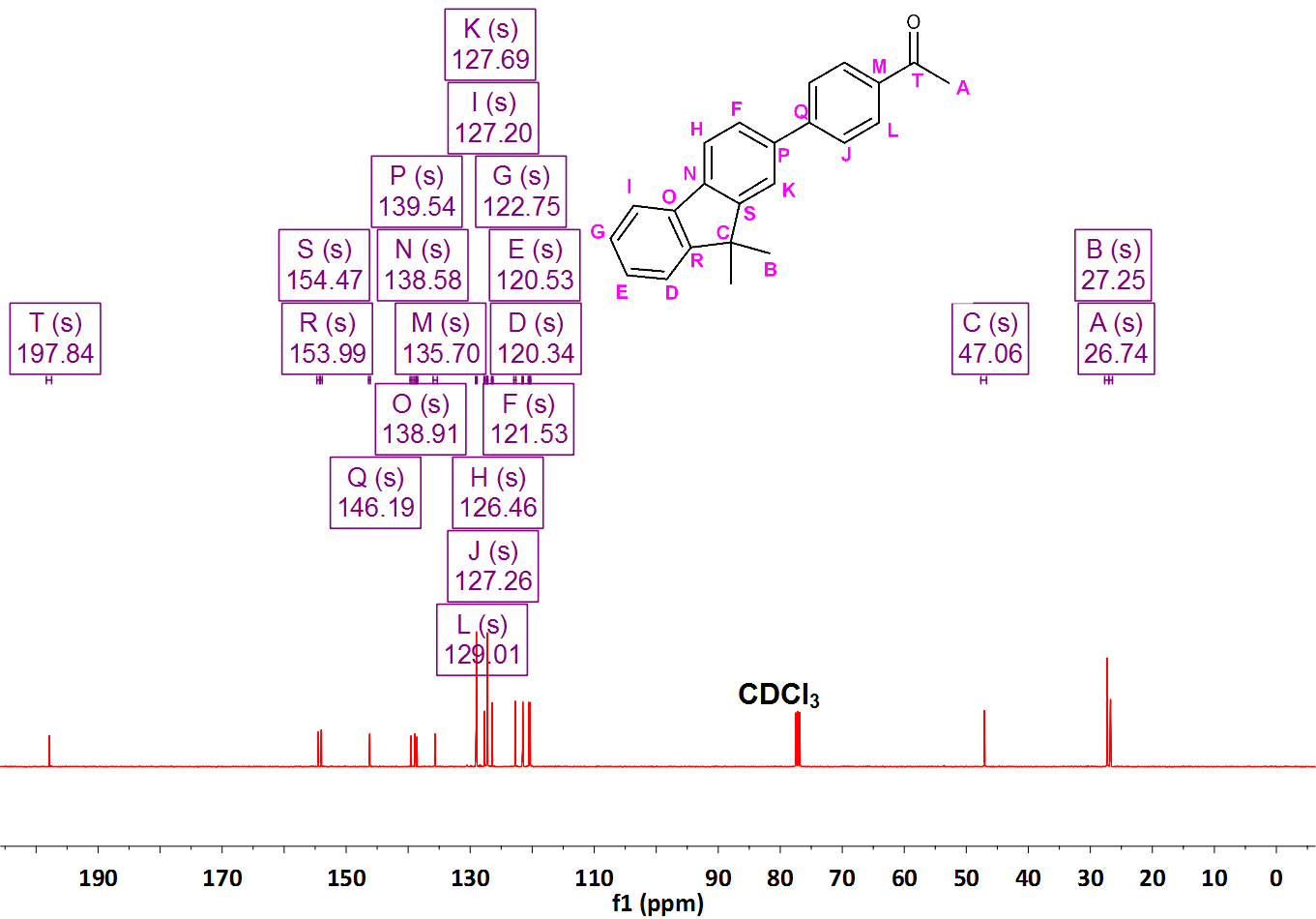
**

**1-(4-(9,9-dimethyl-9H-fluoren-2-yl)phenyl)ethanone (4a_2_)**

**
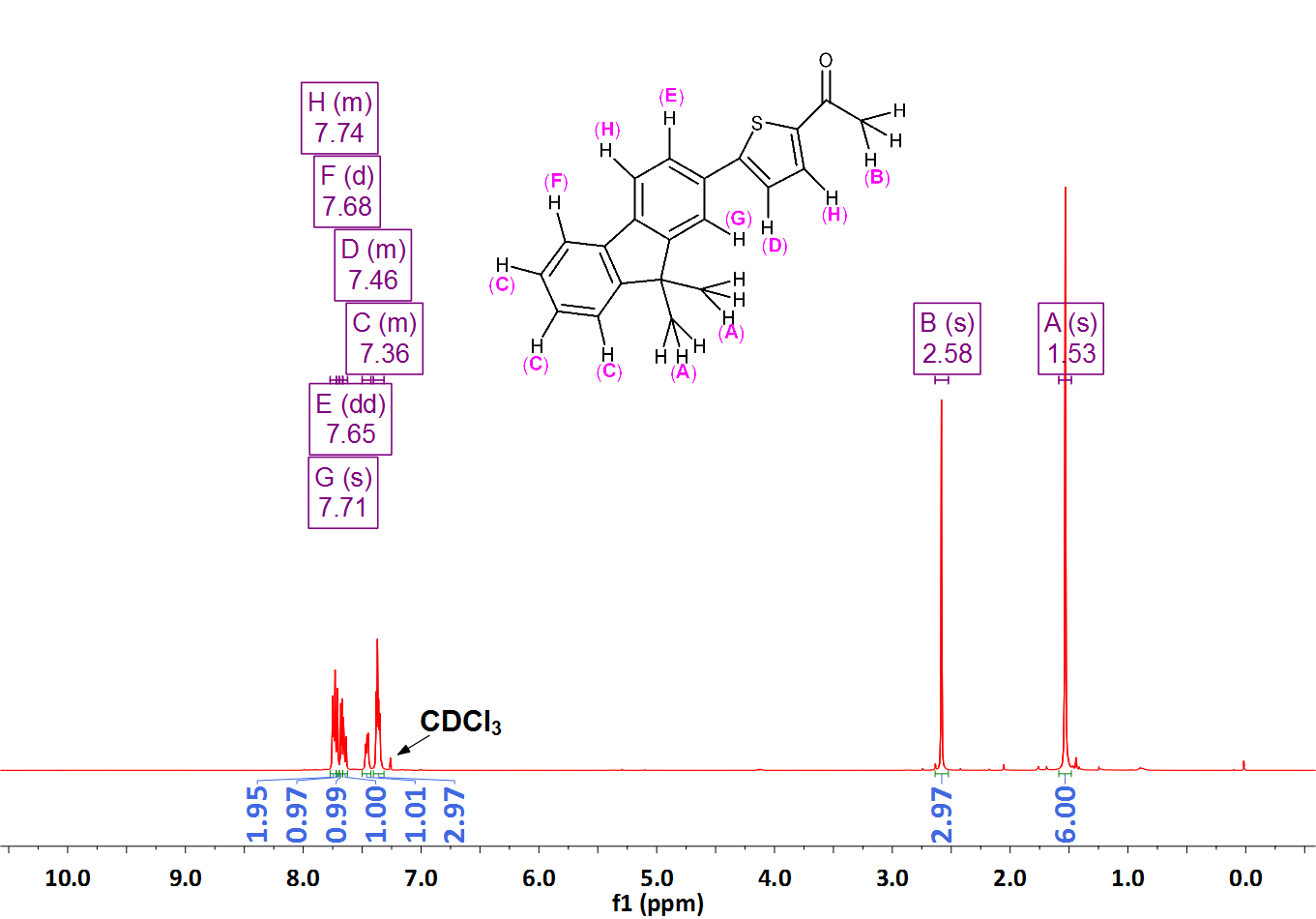
**

**
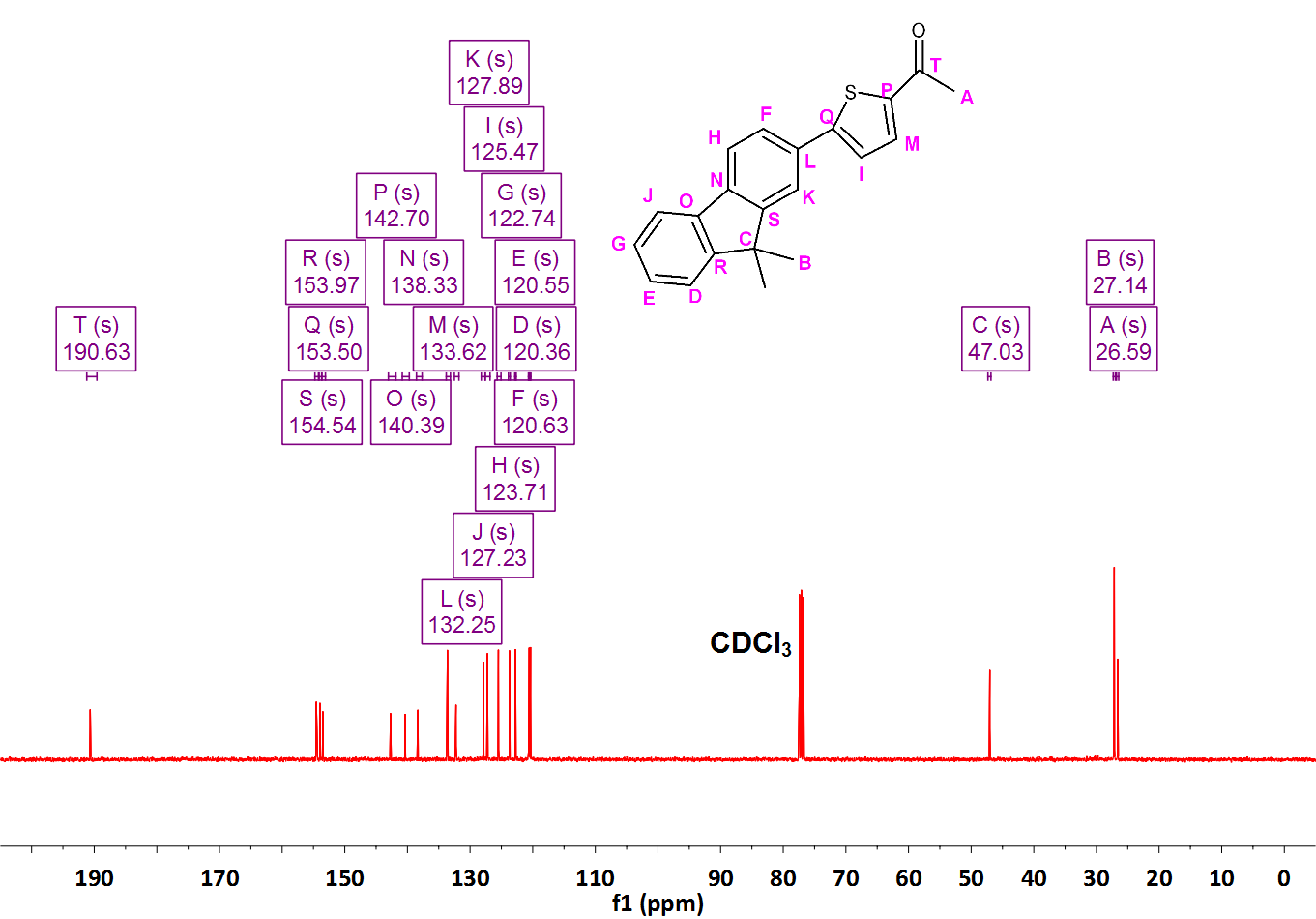
**

**1-(4-(octyloxy)phenyl)ethanone (4a_3_)**

**
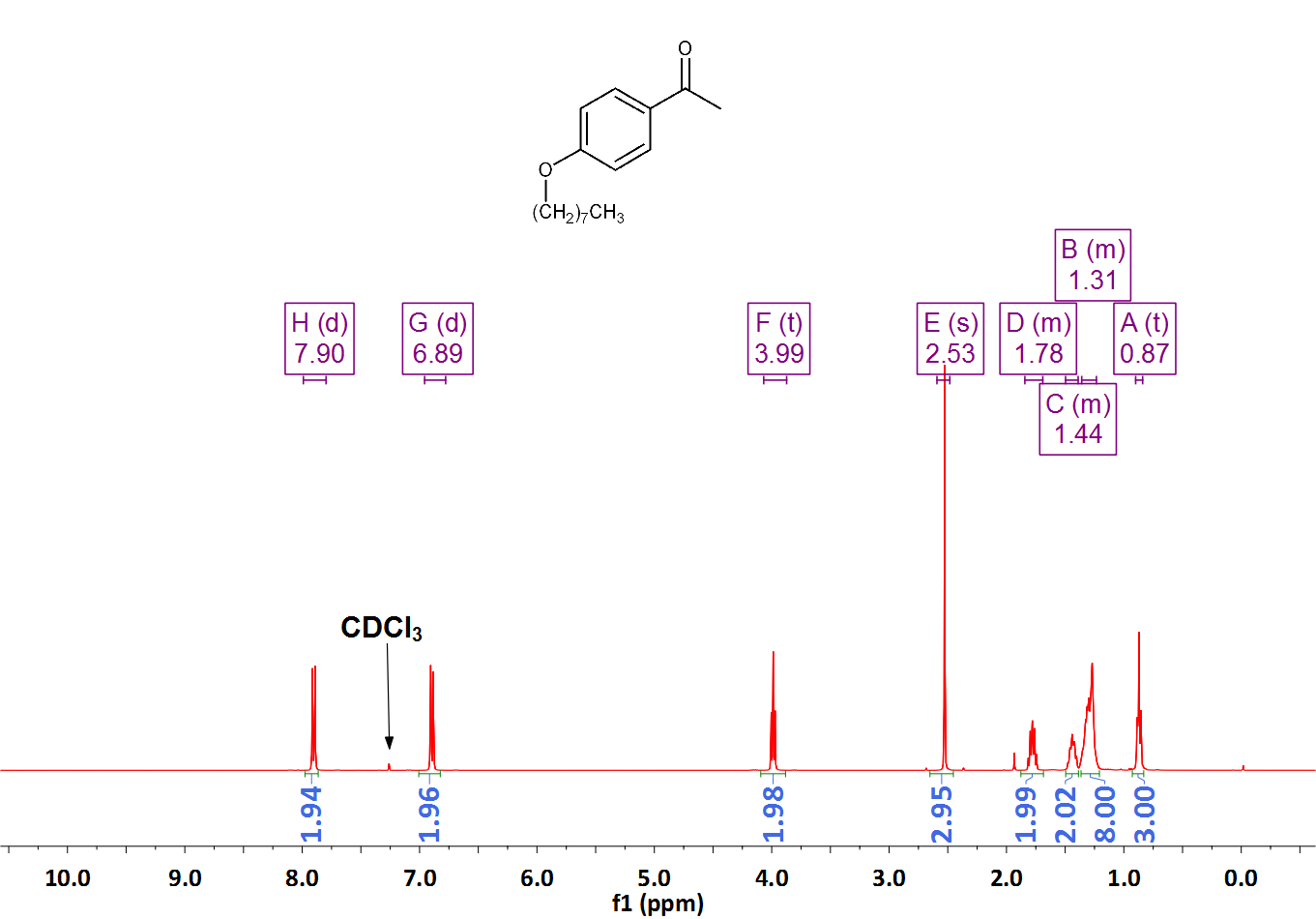
**

**
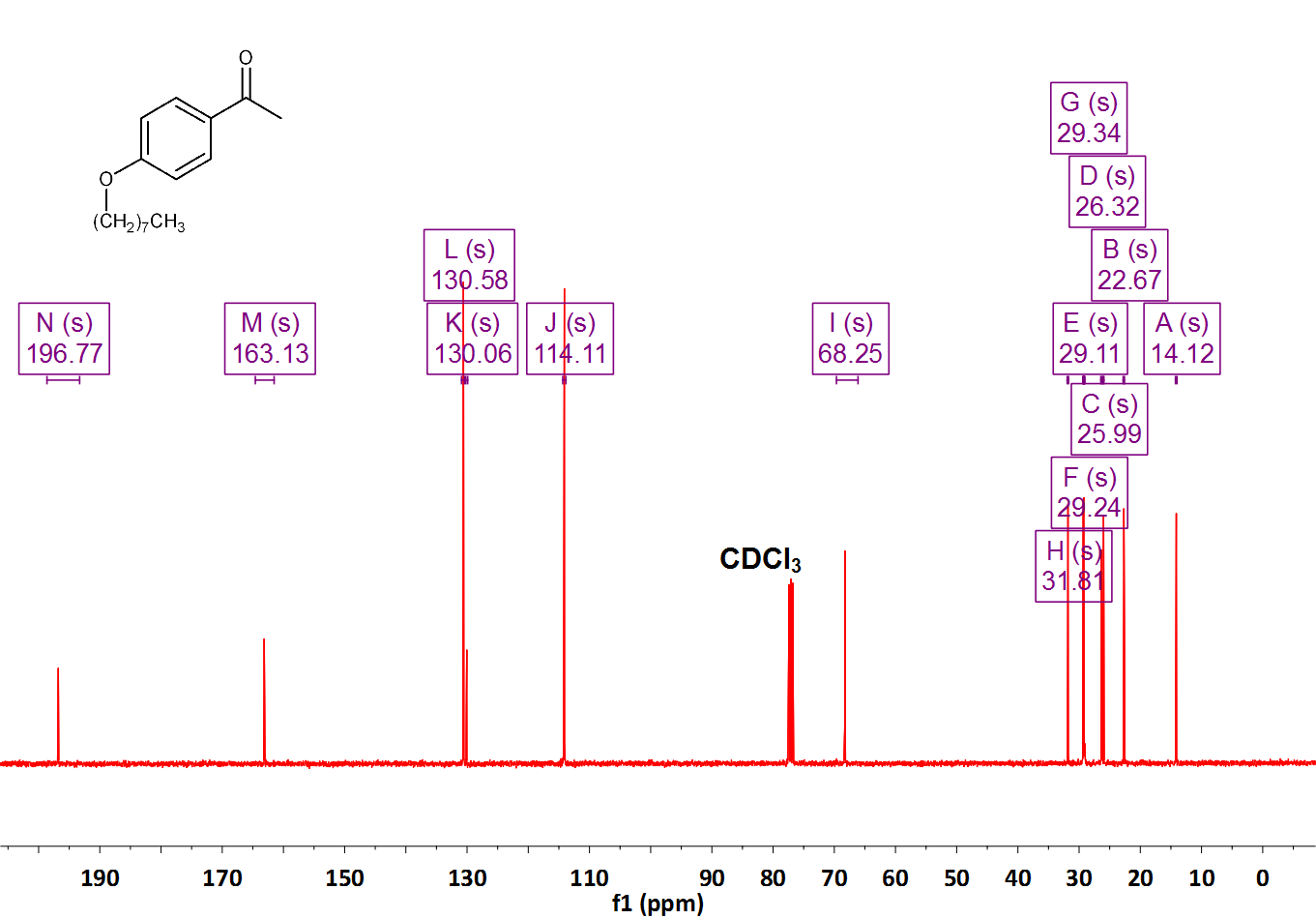
**

**(Z)-3-amino-1-phenyl-3-(pyrazin-2-yl)prop-2-en-1-one (5)**

**
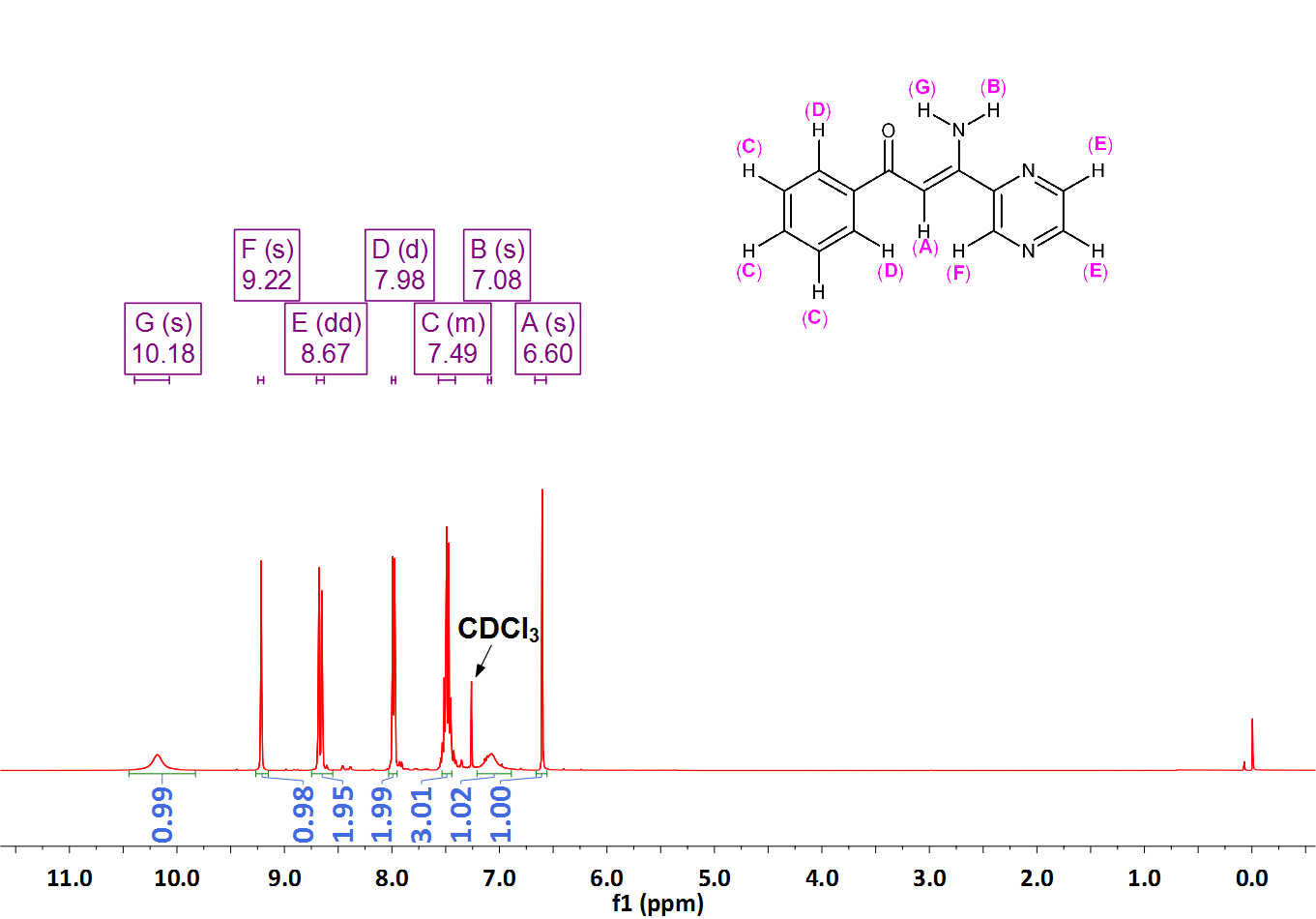
**

**
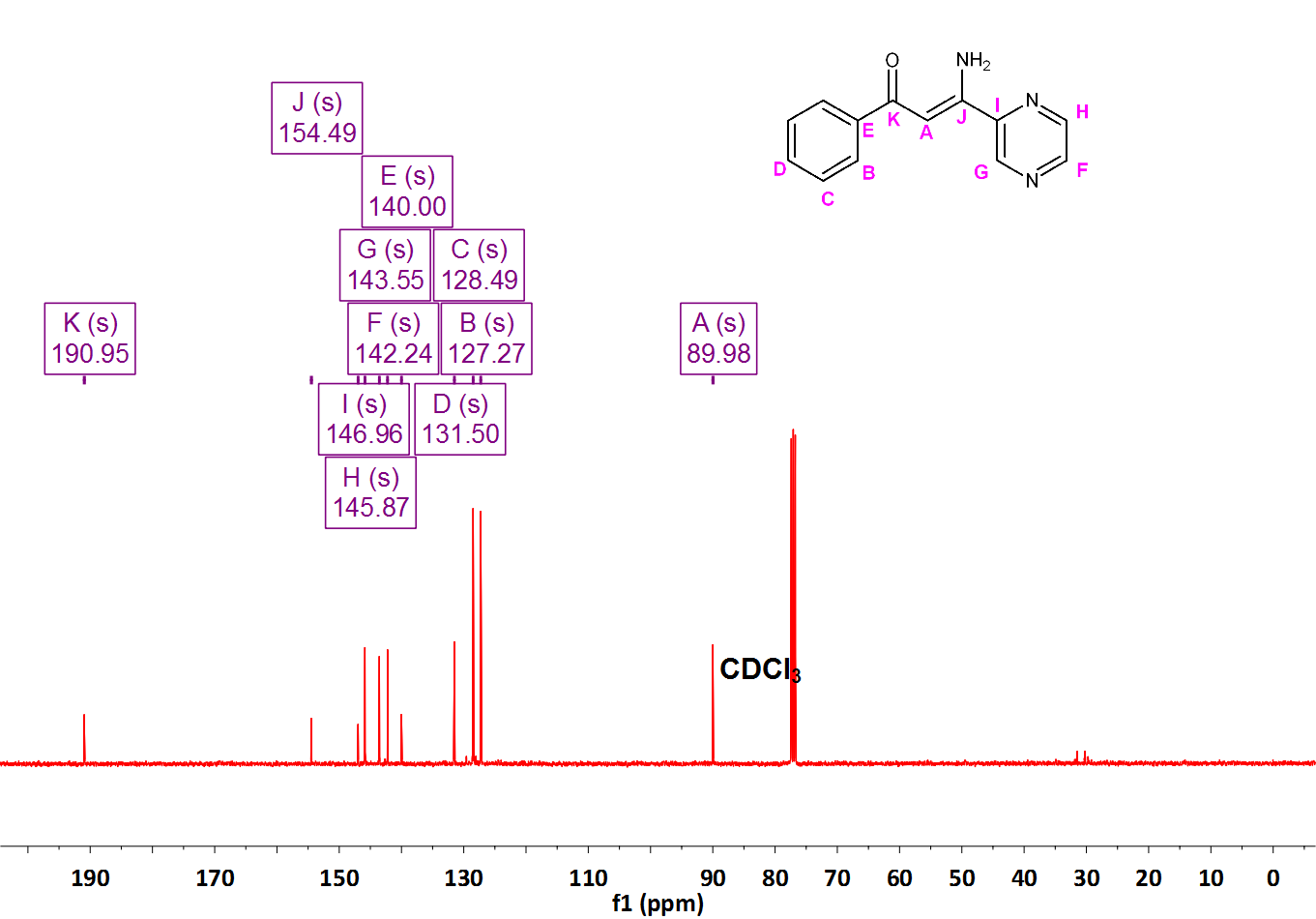
**

**7. IR spectrum of compound 4a**

**
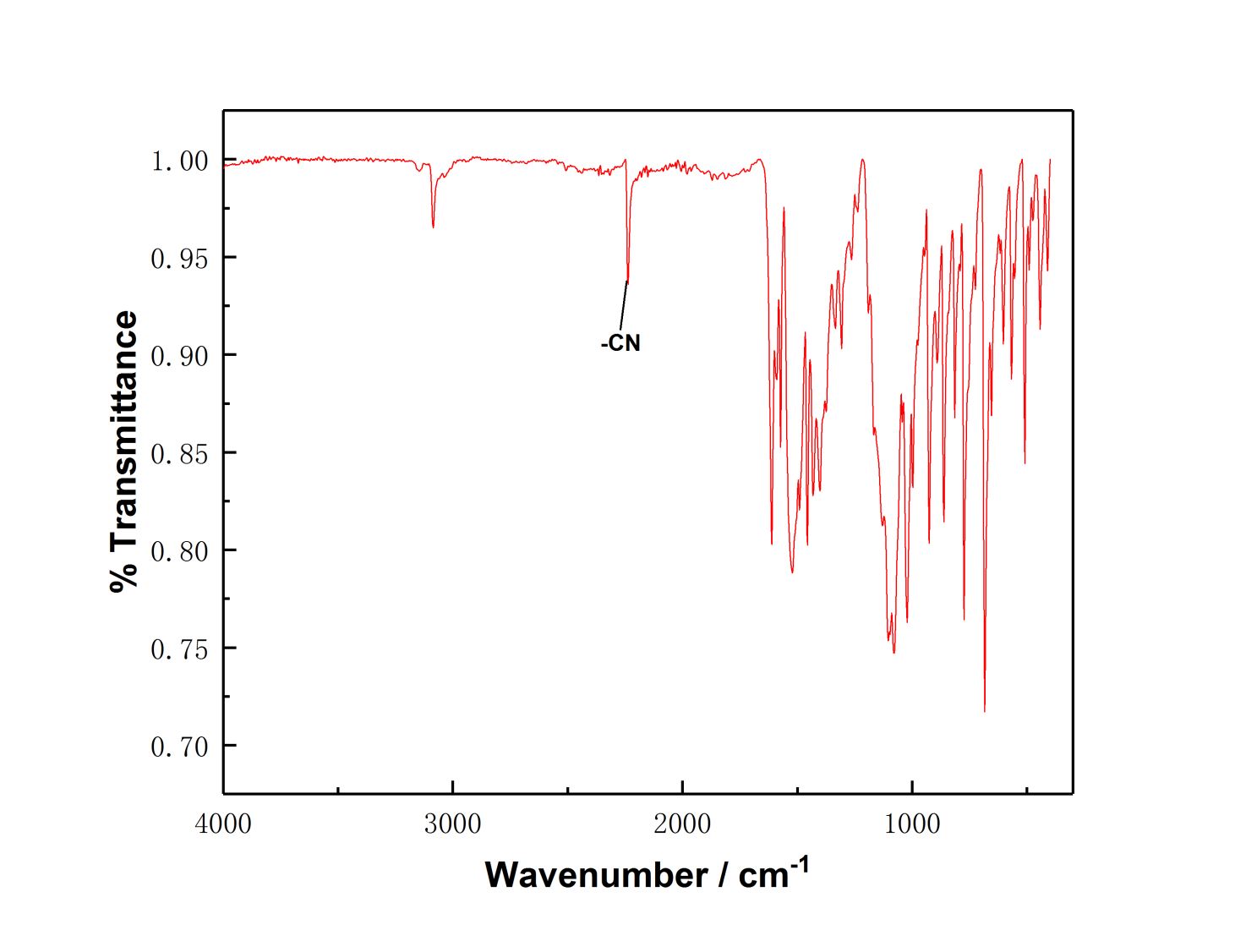
Fig. S2** IR spectrum of compound **4a**.

**8. Excitation-Emission-Matrix spectra of compound 4a**

**Fig. S3** Excitation-Emission-Matrix spectra of compound **4a**.

**9. UV absorption and fluorescence data of compound 4 series molecules**

**9.1 UV absorption and fluorescence emission data of compound 4 series molecules in dichloromethane at a concentration of 2🞨10^-5^ mol∙L^-1^**

Table S2 Spectral data, PLQY and luminescence lifetimes of compounds in dichloromethane

| Comp. | λ_max_[nm] | F_max_[nm] | Stokes shifts[cm^-1^] | Φ_f_,% | τ_s_[ns] |
| --- | --- | --- | --- | --- | --- |
| **4a** | 433 | 472 | 1908 | 75 | 3.5 |
| **4b** | 443 | 507 | 2850 | 79 | 4.3 |
| **4c** | 469 | 542 | 2872 | 70 | 3.5 |
| **4d** | 453 | 483 | 1371 | 76 | 3.9 |
| **4e** | 448 | 483 | 1618 | 76 | 3.5 |
| **4f** | 447 | 481 | 1581 | 76 | 3.5 |
| **4g** | 459 | 497 | 1666 | 70 | 3.3 |
| **4h** | 425 | 476 | 2521 | 59 | 2.1 |
| **4i** | 445 | 474 | 1375 | 72 | 3.7 |
| **4j** | 449 | 475 | 1219 | 63 | 3.2 |
| **4k** | 432 | 473 | 2007 | 60 | 3.2 |
| **4l** | 432 | 490 | 2740 | 82 | 4.0 |
| **4m** | 439 | 492 | 2454 | 87 | 4.2 |
| **4n** | 427 | 477 | 2455 | 83 | 4.2 |
| **4o** | 434 | 479 | 2165 | 78 | 4.1 |
| **4p** | 417 | 485 | 3362 | 81 | 4.2 |
| **4q** | 460 | 535 | 3048 | 47 | 2.8 |
| **4r** | 435 | 497 | 2868 | 53 | 3.1 |
| **4s** | 460 | 524 | 2655 | 62 | 3.5 |
| **4t** | 439 | 519 | 3511 | 63 | 3.5 |
| **4u** | 465 | 497 | 1384 | 62 | 3.1 |
| **4aa** | 462 | 600 | 4980 | 19 | 0.8 |
| **4ab** | 510 | 615 | 3350 | 19 | 0.8 |
| **4ac** | 455 | 510 | 2370 | 80 | 3.9 |

**
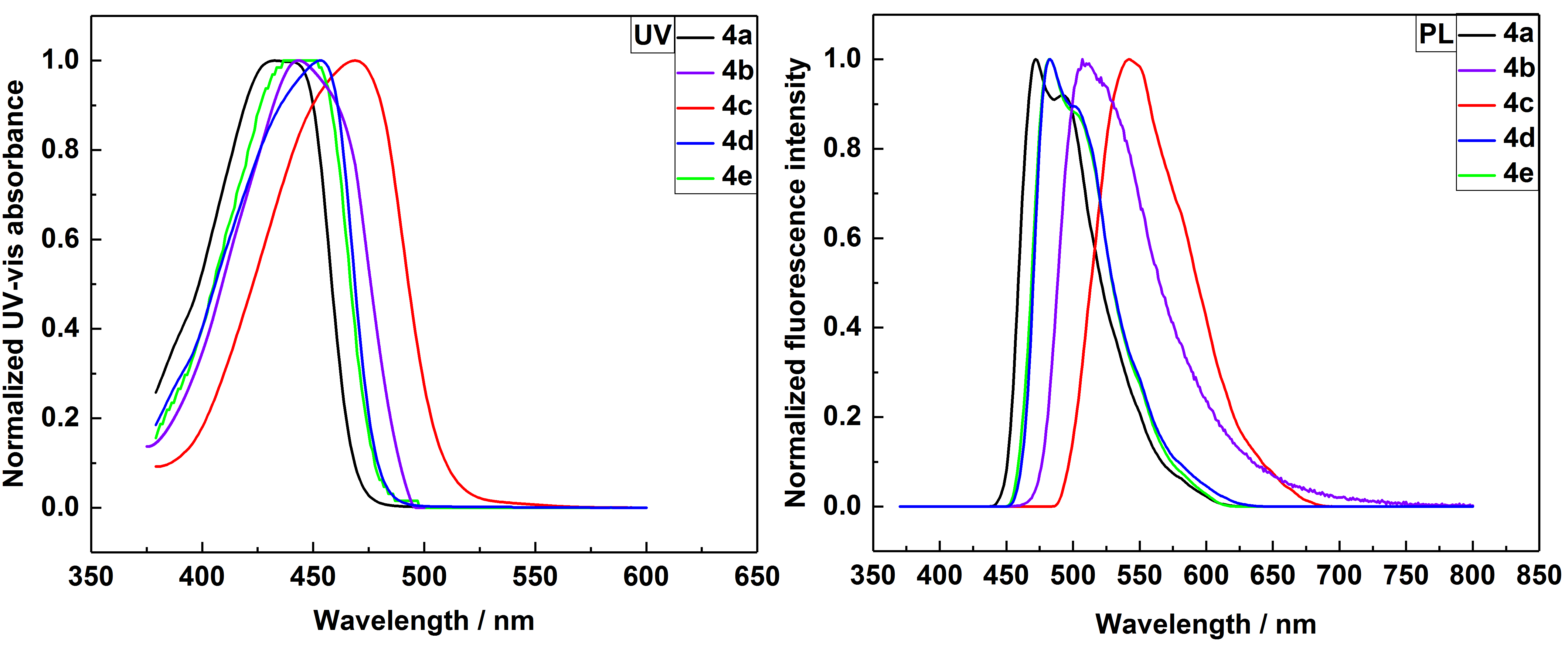
**

**
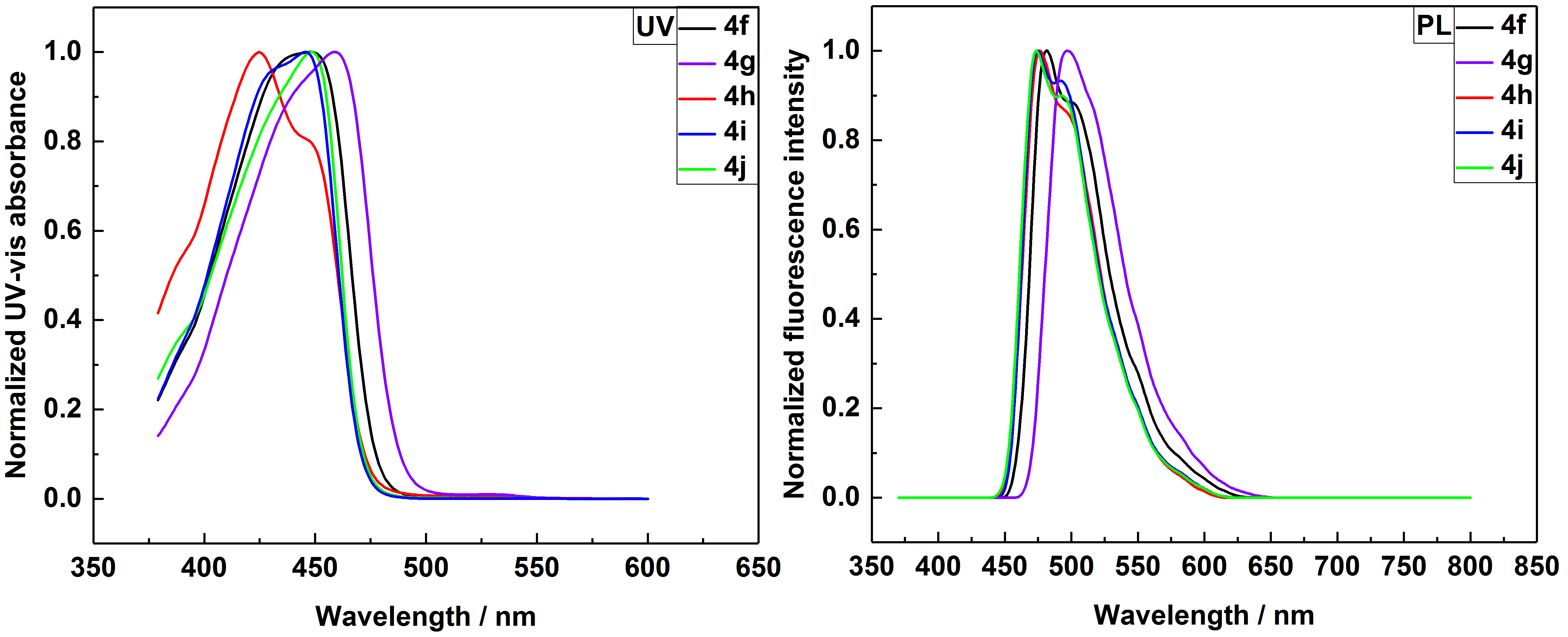
**

**
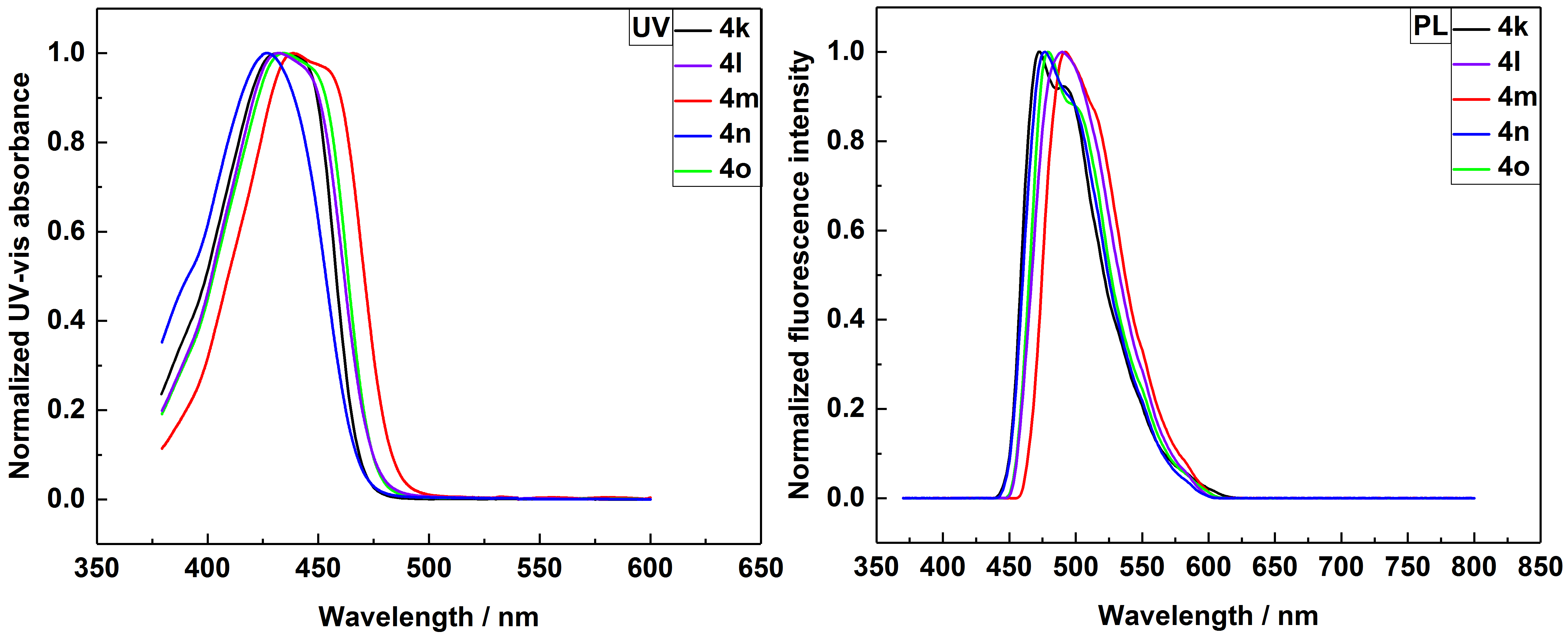
**

**
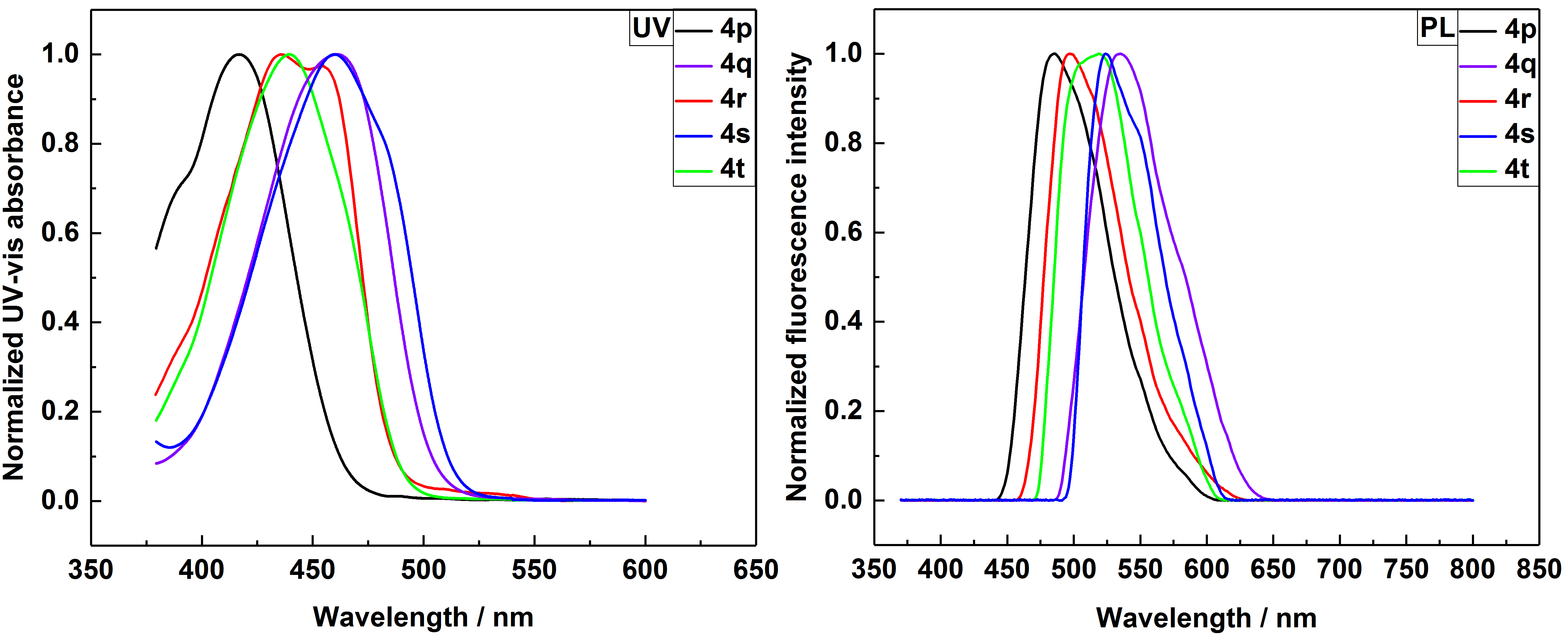
**

**
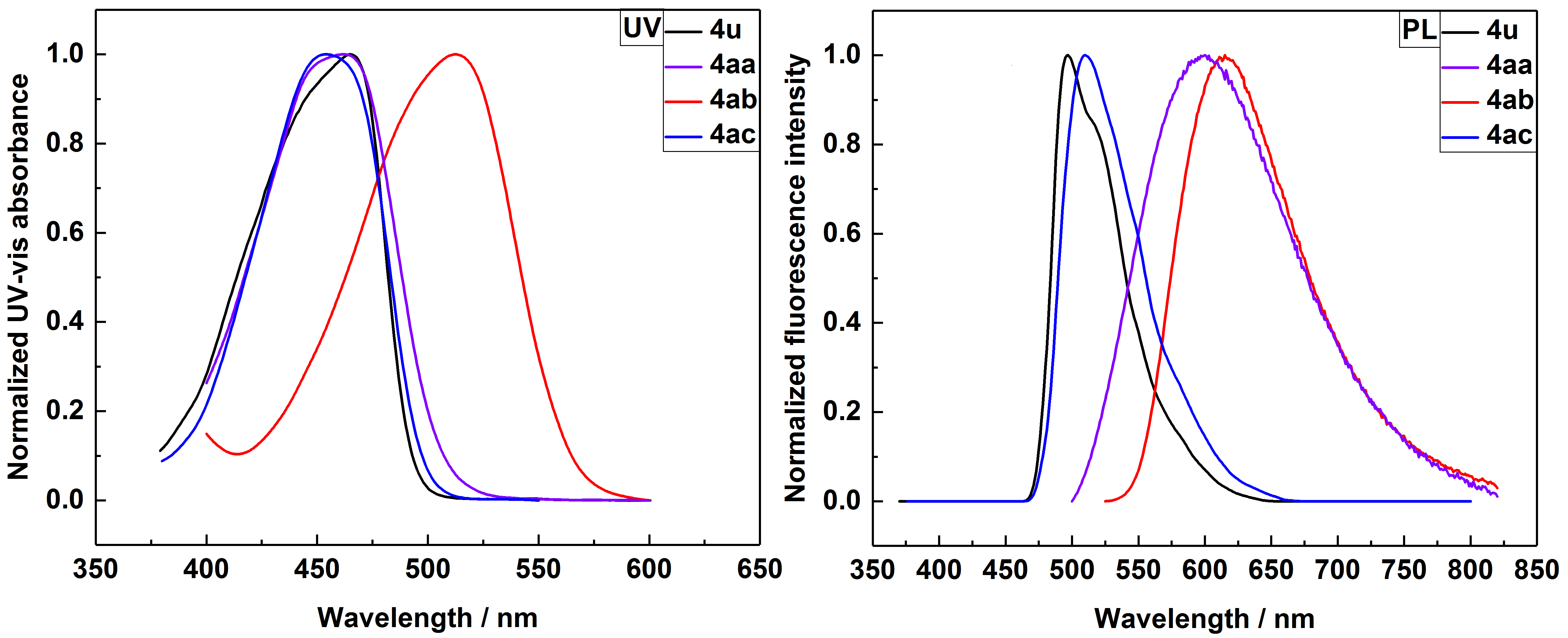
**

**9.2 UV absorption and fluorescence emission data of compound 4 series molecules in solid state**

Table S3 PLQY and luminescence lifetimes of compounds in solid state

| Comp. | Φ_f_,% | τ_s_[ns] | Comp. | Φ_f_,% | τ_s_[ns] |
| --- | --- | --- | --- | --- | --- |
| **4a** | 6 | 2.8 | **4m** | 23 | 4.1 |
| **4b** | 5 | 0.9 | **4n** | 20 | 3.5 |
| **4c** | 3 | 0.5 | **4o** | 2 | 1.9 |
| **4d** | 7 | 1.5 | **4p** | 6 | 1.9 |
| **4e** | 9 | 1.4 | **4q** | 2 | 0.2 |
| **4f** | 11 | 1.5 | **4r** | 3 | 0.4 |
| **4g** | 12 | 1.5 | **4s** | 4 | 0.3 |
| **4h** | 2 | 0.3 | **4t** | 3 | 0.4 |
| **4i** | 3 | 1.7 | **4u** | 3 | 0.2 |
| **4j** | 4 | 1.3 | **4aa** | 2 | 0.1 |
| **4k** | 4 | 1.1 | **4ab** | 1 | 0.2 |
| **4l** | 15 | 3.1 | **4ac** | 10 | 3.8 |

**
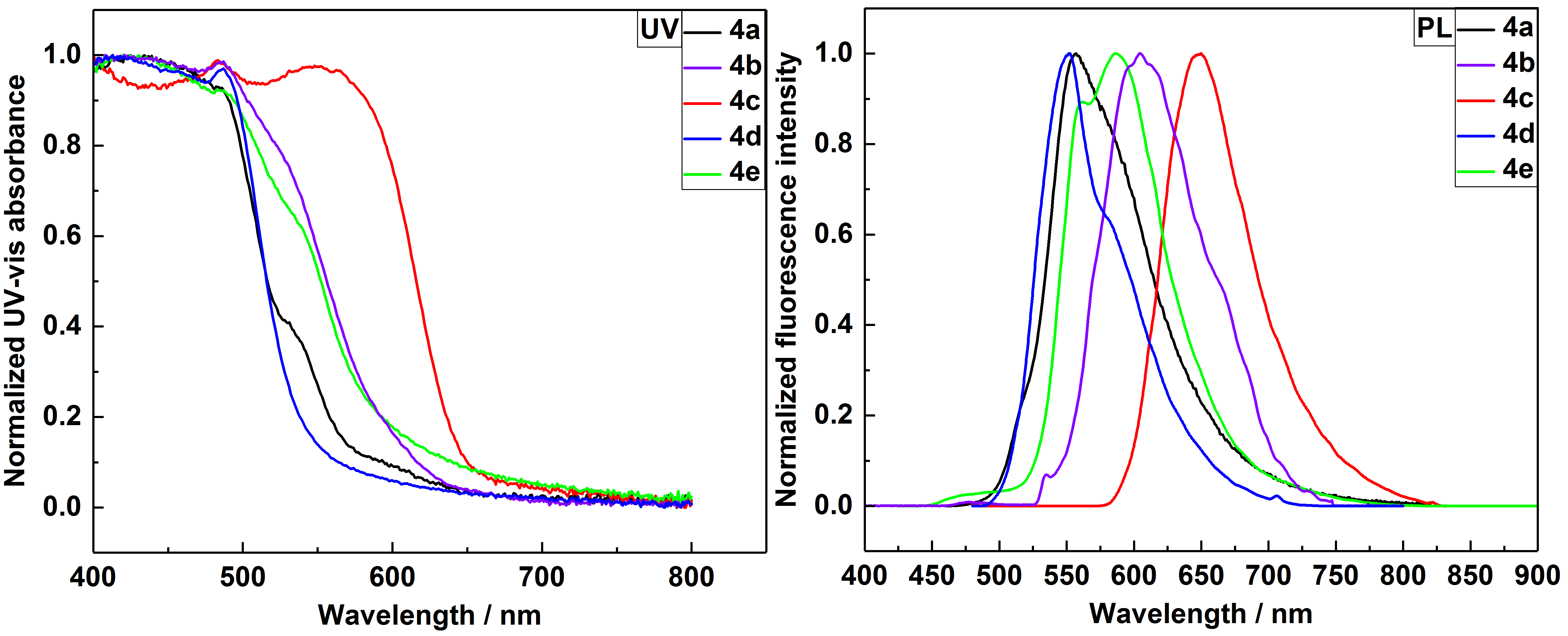
**

**
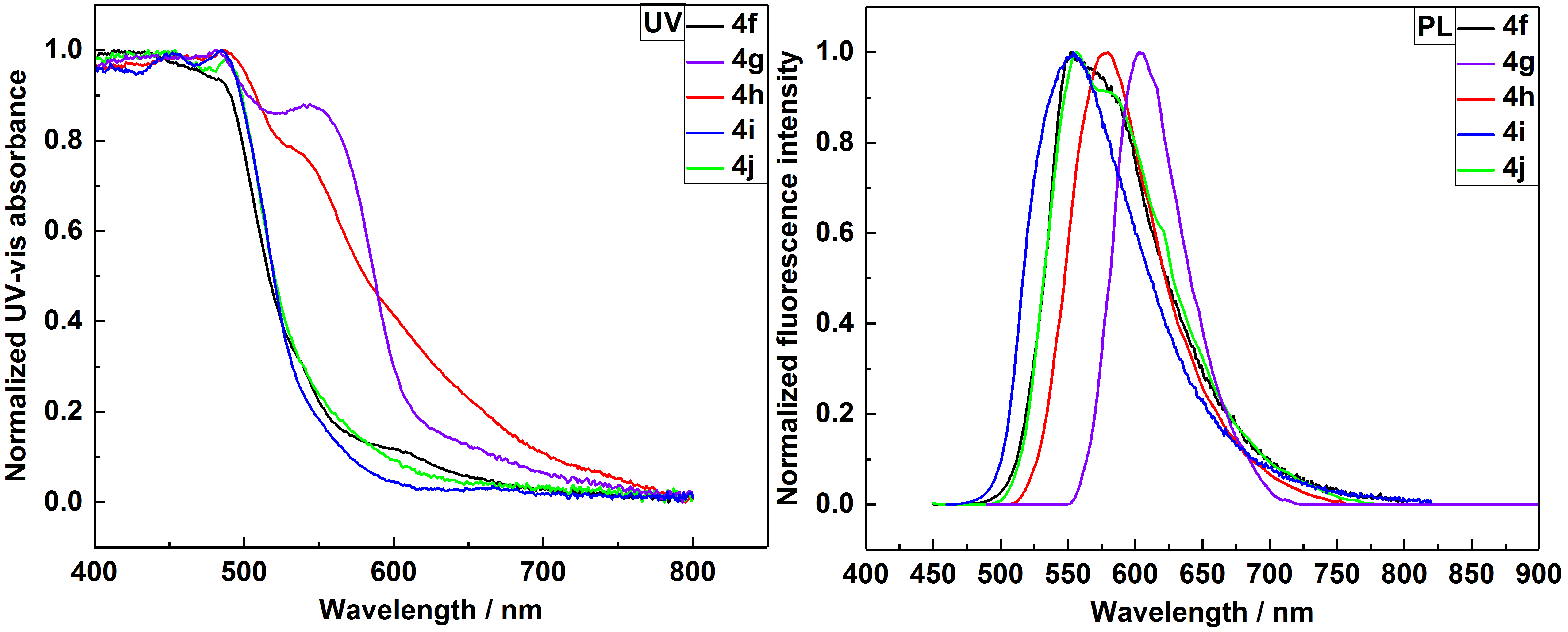
**

**
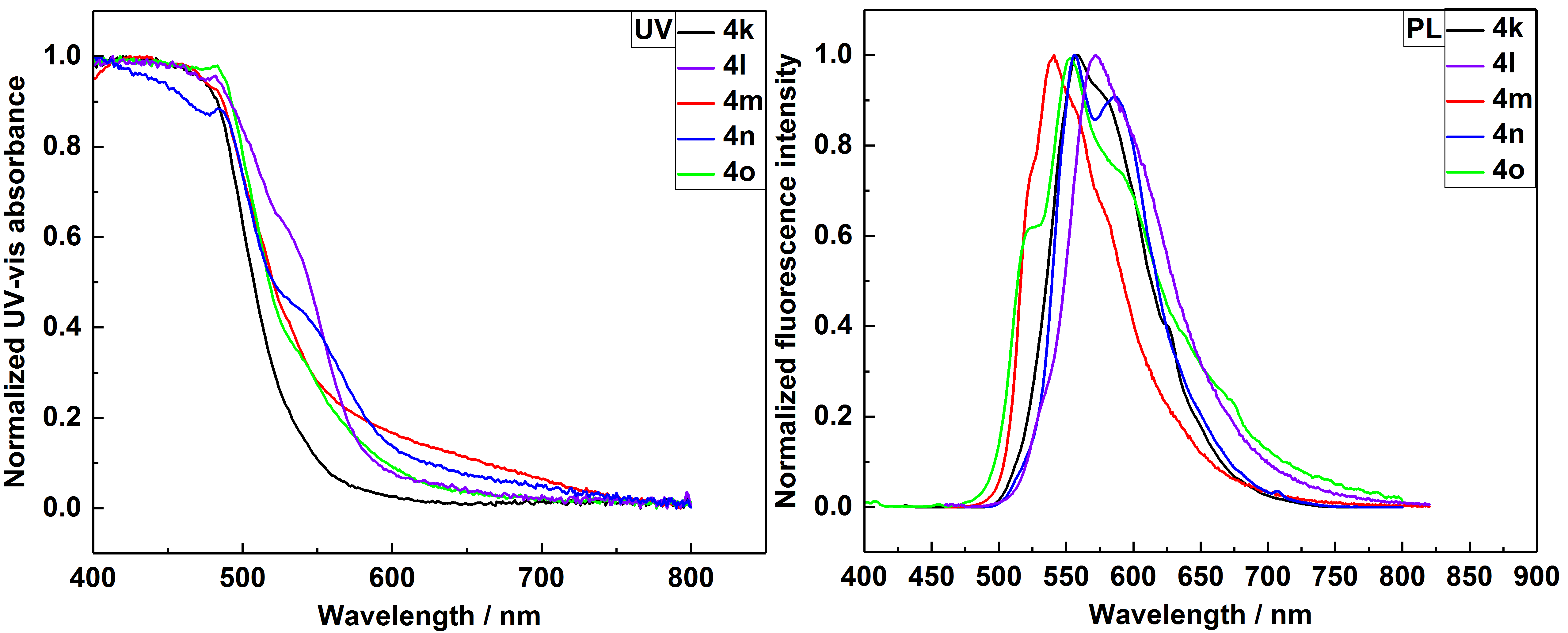
**

**
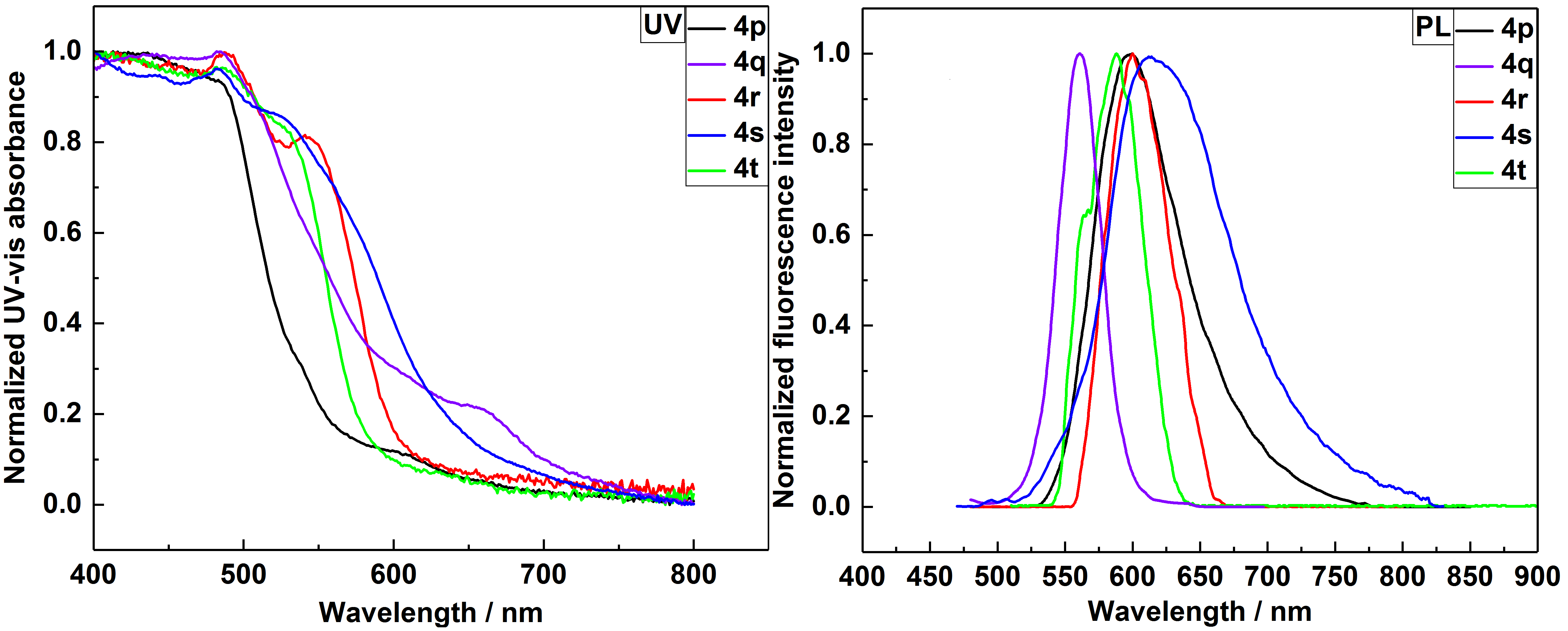
**

**
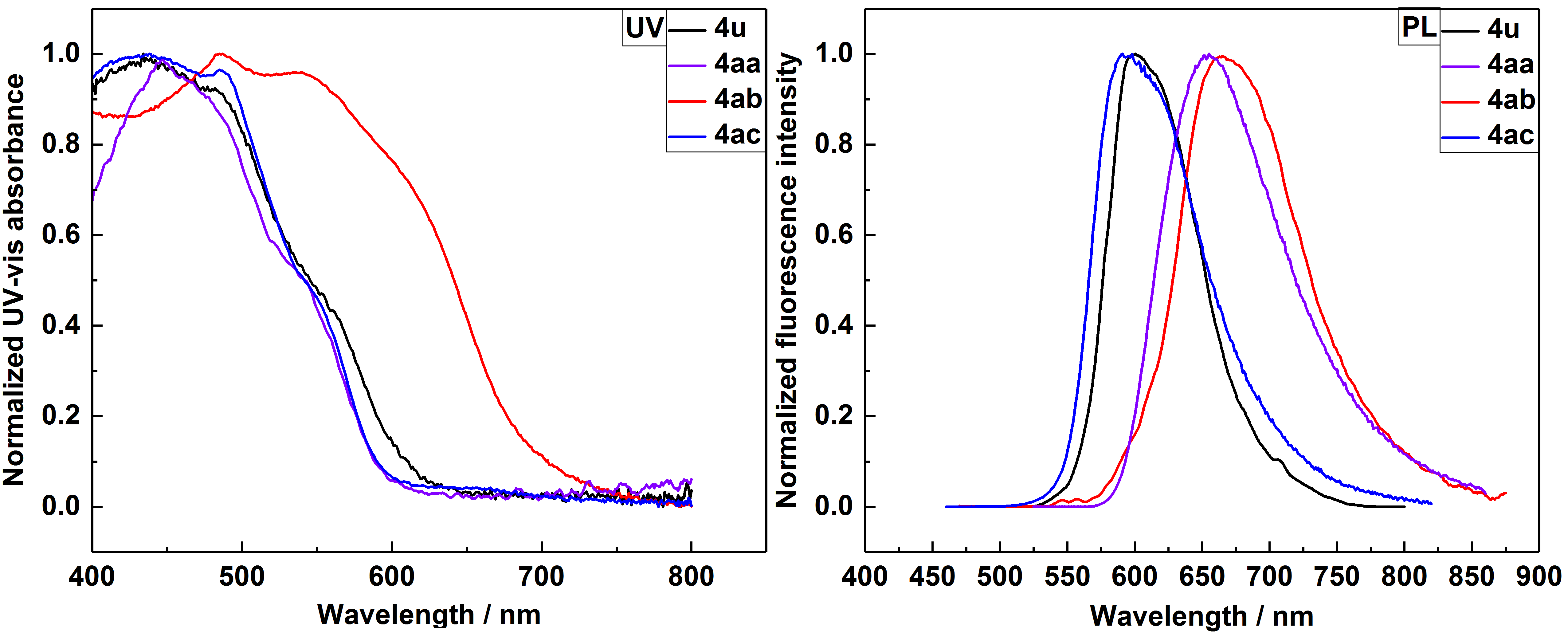
**

**10. References**

1. CrysAlisPro, Oxford Diffraction (Poland), **2010**.

2. Sheldrick, G.M. SHELXS-97, Program for the Solution of Crystal Structure. University of

Göttingen, Germany. **1997**.
